# Genome-wide analysis of salt-responsive and novel microRNAs in *Populus euphratica *by deep sequencing

**DOI:** 10.1186/1471-2156-15-S1-S6

**Published:** 2014-06-20

**Authors:** Jingna Si, Tao Zhou, Wenhao Bo, Fang Xu, Rongling Wu

**Affiliations:** 1 Center for Computational Biology, National Engineering Laboratory for Tree Breeding, College of Biological Sciences and Biotechnology, Beijing Forestry University, Beijing 100083, China

**Keywords:** High-throughput sequencing, microRNA, *Populus euphratica*, Salt stress

## Abstract

**Background:**

*Populus euphratica *is a representative model woody plant species for studying resistance to abiotic stresses such as drought and salt. Salt stress is one of the most common environmental factors that affect plant growth and development. MicroRNAs (miRNAs) are small, noncoding RNAs that have important regulatory functions in plant growth, development, and response to abiotic stress.

**Results:**

To investigate the miRNAs involved in the salt-stress response, we constructed four small cDNA libraries from *P. euphratica *plantlets treated with or without salt (300 mM NaCl, 3 days) in either the root or leaf. Using high-throughput sequencing to identify miRNAs, we found 164 conserved miRNAs belonging to 44 families. Of these, 136 novel miRNAs were from the leaf, and 128 novel miRNAs were from the root. In response to salt stress, 95 miRNAs belonging to 46 conserved miRNAs families changed significantly, with 56 miRNAs upregulated and 39 miRNAs downregulated in the leaf. A comparison of the leaf and root tissues revealed 155 miRNAs belonging to 63 families with significantly altered expression, including 84 upregulated and 71 downregulated miRNAs. Furthermore, 479 target genes in the root and 541 targets of novel miRNAs in the leaf were predicted, and functional information was annotated using the Gene Ontology and Kyoto Encyclopedia of Genes and Genomes databases.

**Conclusions:**

This study provides a novel visual field for understanding the regulatory roles of miRNAs in response to salt stress in *Populus*.

## Background

MicroRNAs (miRNAs) are a class of endogenous noncoding single-stranded RNAs of about 21-23 nucleotides (nt) in length, which participate in the posttranscriptional regulation of flora and fauna gene expression [1,2]. The miRNAs were first discovered in *Caenorhabditis elegans *in 1993 [3]. To date, 24,521 miRNAs have been identified in animals [4], plants [4], and viruses [5] (MiRBase Release20: June 2013) [6]. Most miRNAs exist as single copies, multiple copies, or gene clusters in the genome. The identification and analysis of plant miRNAs have focused on several model species including *Arabidopsis thaliana *and *Oryza sativa*. However, only four miRNA families have been identified in *Populus euphratica *in the miRBase Release20. Recent findings showed that miRNAs play important roles in response to various abiotic stresses in plants, including high salinity [7,8], drought [9-12], low temperatures [7,13], oxidative stress [14], hypoxic stress [15,16], UV-B radiation [17], and mechanical stress [17,18].

Salt stress is one of the major blocks in agricultural and forestry growth and production in modern times. To resist high-salinity stress and sustain their growth, plants have evolved multiple gene regulatory profiles to regulate water and ion balance and maintain normal photosynthesis. These regulatory genes are involved in a series of physiological, biochemical, and cellular processes essential for energy metabolism, photosynthesis, signal transduction, transcription, and protein biosynthesis and decay. In recent years, several studies have reported on the transcriptional regulation of specific miRNAs and genes in response to the salt-stress environment [19-21]. Using the microarray method, Liu et al. discovered 10 miRNAs in *Arabidopsis *that showed differential expression under salt-treatment conditions [7]. In addition, miR393 was strongly upregulated when treated with 300 mM NaCl [22]. In rice, miR169g was upregulated during high-salinity stress, and the transgenic plants that overexpressed miR393 were more sensitive to salt treatment than control plants [23,24]. In microarray studies focused on forestry species, several miRNAs such as miR395, miR398, and miR399 in *Populus tremula *were upregulated under salt stress. Notably, however, miR398 was downregulated in salt-treated *Arabidopsis *[25]. MiR168, miR1444, and miR1446 expression levels were greatly altered under salt conditions in *P. euphratica *[26]. In *Populus trichocarpa*, the expression of a large number of miRNAs was influenced by many environmental factors including salt stress [27]. Despite these advances, the regulatory mechanisms of miRNAs in plant growth and development remain undefined, and more in-depth studies on miRNA expression in response to salt stress in plants are required, especially for *P. euphratica*, a tree species known for its strong resistance to salinity. In addition, little research has focused on the systemic identification of salt-responsive miRNAs in *P. euphratica *at the genome level using high-throughput sequencing.

The poplar species *P. euphratica *grows almost exclusively in the desert. A great majority of *P. euphratica *are grown in China, and 90% of these are distributed in the Tarim River Basin in Xinjiang Province [28]. *P. euphratica *has a high tolerance for salinity, drought, cold, and wind, which makes it one of the only tree species in the Taklimakan Desert [29]. Thus, *P. euphratica *is widely accepted as an ideal model species for studying the abiotic stress resistance of woody plants [30]. Studies on *P. euphratica *miRNAs in response to salt stress may expand the understanding of the mechanism of gene function and regulation in resistance to stress [31]. In this study, the high-throughput sequencing method, which has been used widely for miRNA research [10-13,32,33], was used to identify conserved and novel miRNAs of *P. euphratica *in the roots and leaves. We analyzed the expression levels of these miRNAs in the different tissues under salt treatment and in controls, and investigated the potential roles of their target genes.

## Methods

### Plant materials and stress treatment

The experimental materials were poplar cutting clones from 2-year-old robust *P. euphratica *plantlets from Korla, Xinjiang Province. Briefly, seedlings were grown 10 cm apart above ground in a greenhouse for 6 months; thereafter, the seedlings were amputated and planted in 2-L plastic pots. After the section buds reached 10-20 cm, healthy sprouts were selected, cut to approximately 10 cm stem lengths, soaked in 0.01% ABT1 solution for 30 min, and then inserted into a mixture of vermiculite, perlite, and peat in a 1:1:1 matrix to cultivate and maintain adequate soil moisture. Seedlings were grown in a greenhouse for 1 year, and 10 clones were selected for the experiments.

For the salt treatment, 10 *P. euphratica *plantlets were grown in 2-L plastic containers in a greenhouse at Beijing Forestry University. In the salt-treated group (*n *= 5) plants were watered using a 300 mM NaCl solution to saturate the soil two times per week. The control group (*n *= 5) was irrigated using pure water twice weekly. After 3 weeks, the leaves and roots from the salt-treated and control groups were selected, frozen immediately in liquid nitrogen, and stored at -80°C until RNA extraction.

### RNA extraction followed by construction and sequencing of small RNA libraries

Total RNA was extracted three times from the salt-treated and control plantlets using a modified cetyltrimethylammonium bromide (CTAB) procedure [34]. Four sRNA libraries were constructed [11,35] using RNA extracted from the salt-treated leaves (3dSL), salt-treated roots (3dSR), untreated leaves (control, 3dCKL), and untreated roots (control, 3dCKR). After detecting the RNA integrity with 2% agarose gel electrophoresis, the four libraries were sequenced by Solexa sequencing (Illumina, USA) at the Beijing Genomics Institute (BGI), Shenzhen, China.

### Bioinformatic analysis of miRNAs and target prediction of miRNAs

The original image data obtained from the Solexa sequencer became the raw reads in the base-calling procedure. Next, the contaminant reads were removed such as the sequences with and without insert fragments, low-quality reads, polyA tails, and sequences shorter than 18 nt or longer than 30 nt. The remaining clean reads were used to analyze small RNA sequence types, sequence number, and sequence length distribution, then mapped to the *Populus *genome (http://www.phytozome.net/poplar) using SOAP [36]. The sequences with a perfect match were analyzed further. We excluded the noncoding RNAs identified as rRNAs, scRNAs, snoRNAs, snRNAs, and tRNAs that were annotated by comparisons with the NCBI GenBank (http://www.ncbi.nih.gov/GenBank/) [37] and Rfam10.1 (http://rfam.sanger.ac.uk/) databases. Small RNA tags corresponding to mRNA exons and introns in the *Populus *genome by Overlap were also excluded from further analysis. The remaining unannotated small RNAs were annotated by aligning to miRBase18.0 (http://www.mirbase.org/index.shtml) [38], allowing two mismatches at most. To identify the characteristic secondary structures, the conserved miRNAs weres screened using the program RNAfold (http://www.tbi.univie.ac.at/~ivo/RNA/ViennaRNA-1.8.1.tar.gz) [39]. The remaining reads were used to predict new miRNAs using the prediction software Mireap (http://sourceforge.net/projects/mireap). New criteria that considered the whole developmental progress of miRNAs were used for distinguishing candidate novel miRNAs [40]. The target genes of new miRNAs were predicted according to previous studies [41-43], allowing at most four mismatches in the miRNA/target gene duplex. The predicted target genes were used to annotate the functions and pathways using the Gene Ontology (GO, http://www.geneontology.org/) and Kyoto Encyclopedia of Genes and Genomes (KEGG, http://www.genome.jp/kegg/) [44] databases.

### Differential expression analysis of miRNAs under salt stress

To investigate the expression difference of these samples, we modified the frequency of each miRNA in the different libraries to 1 million (normalized expression = miRNA expression count*1,000,000/total number of clean reads). To reduce error, miRNAs were removed that showed normalized expression of less than 1. The fold-change between the treatment and control samples was calculated as follows:

$$Fold-change={\text{log}}_{2}^{treatment/control}$$

The *P*-value represented the significant degree of miRNA expression differences between samples, with the smaller *P*-value signifying a greater significant difference. It was calculated as follows:

$$p\left(x|y\right)=\left(\frac{N_{2}}{N_{1}}\right)\frac{\left(x+y\right)!}{x!y!{\left(1+\frac{N_{2}}{N_{1}}\right)}^{\left(x+y+1\right)}}$$

$$C\left(y\leq y_{\text{min}}|x\right)=\overset{y\leq y_{\text{min}}}{\underset{y=0}{\sum }}p\left(y|x\right)$$

$$D\left(y\geq y_{\text{max}}|x\right)=\overset{\propto }{\underset{y\geq y_{\text{max}}}{\sum }}p\left(y|x\right)$$

In the formulation, N1 and N2 mean the total number of sample1 and sample2. Meanwhile, × and y represent the expression level of known miRNAs respectively. The smaller P value stands for the greater difference of miRNAs expression between two samples, which was in accord with the statistical law.

## Results

### Analysis of small RNAs in salt-treated plants

To identify salt-responsive miRNAs in *P. euphratica *in leaves and roots, four sRNA libraries were constructed from *P. euphratica *plantlets treated with NaCl solutions or pure water. In total, 16.3 million raw reads were produced in the salt-treated and the control leaves (3dSL and 3dCKL, respectively), 17.7 million raw reads were produced in salt-treated roots (3dSR), and 16.2 million reads were produced in control roots (3dCKR).

**Figure 1:**
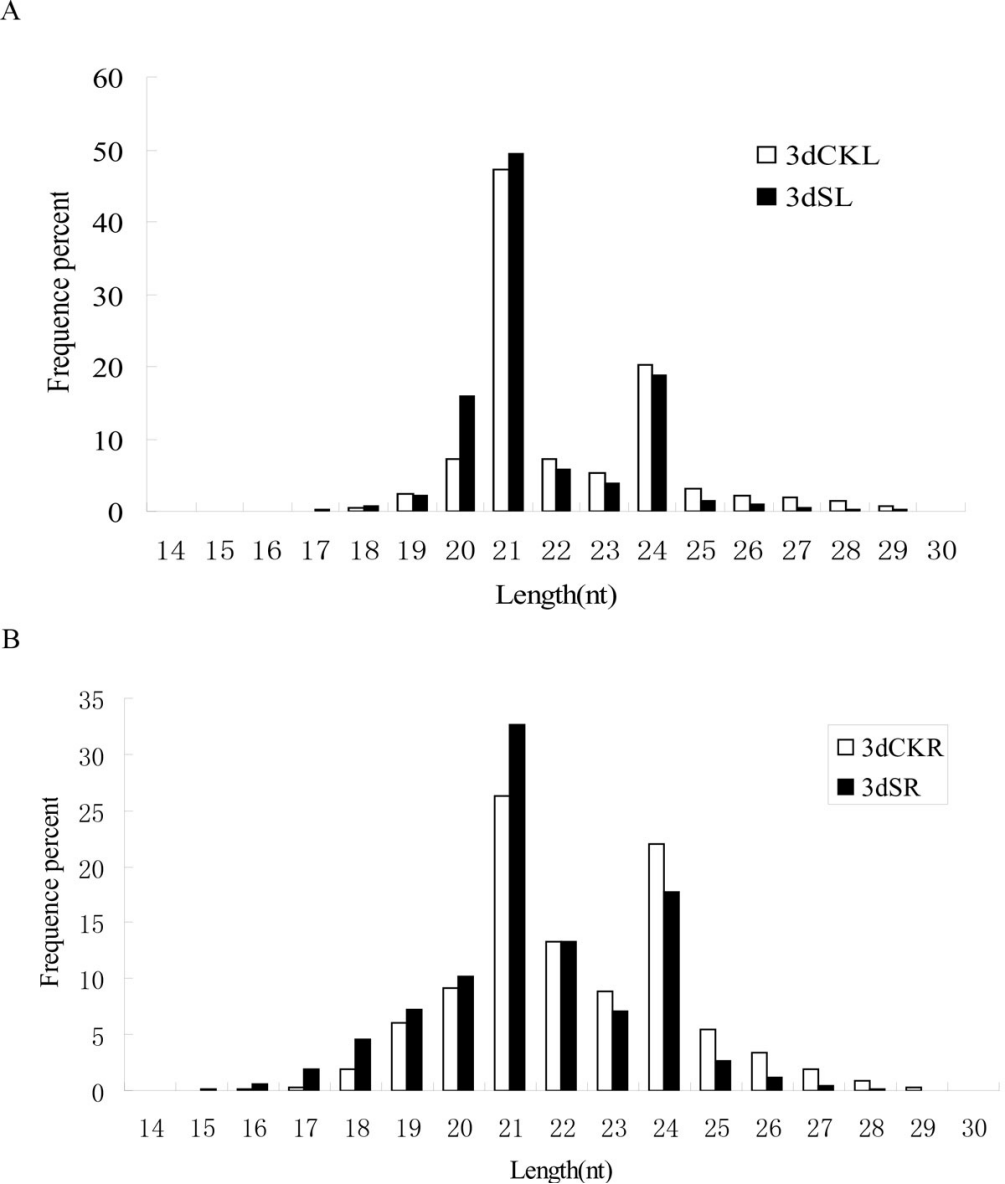
**Size distribution of small RNA reads in different libraries**. (A) Control (3dCKL) and salt stress (3dSL) from *Populus euphratica *leaf libraries. (B) Control (3dCKR) and salt stress (3dSR) from *P. euphratica *root libraries.

After removing redundant reads and those that had low quality, 3′ adapter null, insert null, 5′ adapter contaminants, and polyA tails, more than 95% of the reads were obtained as clean reads with lengths ranging from 18 to 30 nt. At least 70% of the reads had lengths of 20-24 nt (Figure 1). The most abundant reads were 21 nt in length, followed by 24-nt reads (Figure 1), a finding that was consistent with previous results [11,45,46]. This phenomenon was observed not only in leaves, but also in roots. Notably, 49.31% of the reads were 21 nt in 3dSL, and 32.58% were 21 nt in 3dSR, suggesting that a significantly greater number of 21-nt reads in leaves participated in the salt-response process. In addition, the number of 21-nt reads in salt-treated libraries was slightly more than those from the control libraries, suggesting that more 21-nt reads were induced in response to salt stress. Furthermore, greater numbers of 24-nt reads were produced under salt conditions.

**Figure 2:**
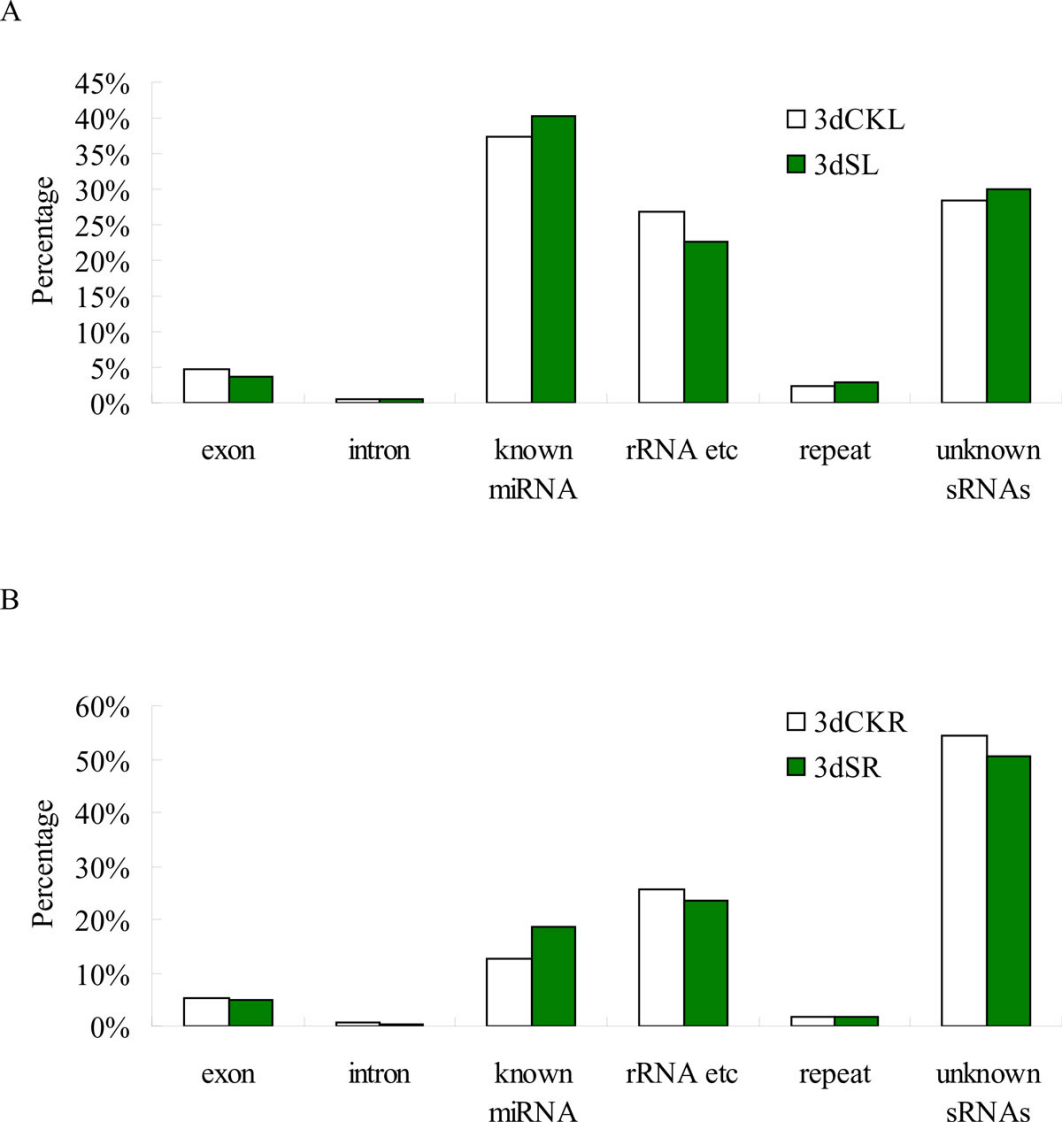
**Distribution of different sRNA annotation categories in total reads of control and salt-stress libraries**. (A) Control (3dCKL) and salt stress (3dSL) from *Populus euphratica *leaf libraries. (B) Control (3dCKR) and salt stress (3dSR) from *P. euphratica *root libraries.

The sRNAs were mapped to the *Populus *genome (http://www.phytozome.net/poplar), miRBase 20.0 version (http://microrna.sanger.ac.uk/sequences), GenBank (http://www.ncbi.nih.gov/genbank/), and Rfam (http://rfam.sanger.ac.uk/) databases. The sequenced sRNAs were annotated and classified into six categories: exons (sense, antisense), introns (sense, antisense), known miRNAs (identified miRNAs in miRBase 20.0), rRNAs (i.e., rRNA, tRNA, snRNA, scRNA, siRNA, and snoRNA) repeat-associated RNAs, and unknown sRNAs (Figure 2). The results showed that the proportion of known miRNAs increased from 37.24% to 40.30% in response to salt stress in leaves and increased from 12.57% to 18.70% under salt stress in roots (Figure 2). This result implies that miRNAs in leaves are more abundant than those in roots, and that these miRNAs had an important function under salt stress. However, the percentage of unknown sRNAs reads increased slightly from 28.36% (3dCKL) to 30.11% (3dSL) and decreased from 54.42% (3dCKR) to 50.67% (3dSR), indicating that large numbers of unknown salt-responsive sRNAs were unidentified (Figure 2). The repeats, exons, and introns revealed little change under the different conditions.

The common and specific sequences were analyzed in different libraries. In total, 3,923,163 unique sRNAs and 32,010,578 total sRNAs were identified in the two leaf libraries (Table 1). Although 13.68% of the sequences were coincident in the two libraries, these sRNAs covered 87.19% of the total sRNAs. In comparison, 6,861,893 unique sRNAs and 32,900,213 total sRNAs were found in the two roots libraries (Table 2). Although 12.40% of the sequences were coincident in both root libraries, these sRNAs covered 77.72% of all sRNAs. The specific percentage in both the salt-treated libraries for unique sRNA reads was about 3% higher than that in the control libraries.

**Table 1:** Summary of common and specific sequences between 3dSL and 3dCKL libraries

Class	Unique sRNA	Percentage	Total sRNA	Percentage
Total sRNAs	3923163	100.00	32010578	100.00
3dSL & 3dCKL	536735	13.68	27911344	87.19
3dSL-specific	1754216	44.71	2092797	6.54
3dCKL-specific	1632212	41.60	2006437	6.27

**Table 2:** Summary of common and specific sequences between 3dSR and 3dCKR libraries

Class	Unique sRNA	Percentage	Total sRNA	Percentage
Total sRNAs	6861893	100.00	32900213	100.00
3dCKR & 3dSR	850624	12.40	25568498	77.72
3dCKR-specific	2903793	42.32	3534735	10.74
3dSR-specific	3107476	45.29	3796980	11.54

### Identification of conserved miRNAs in *P. euphratica*

**Figure 3:**
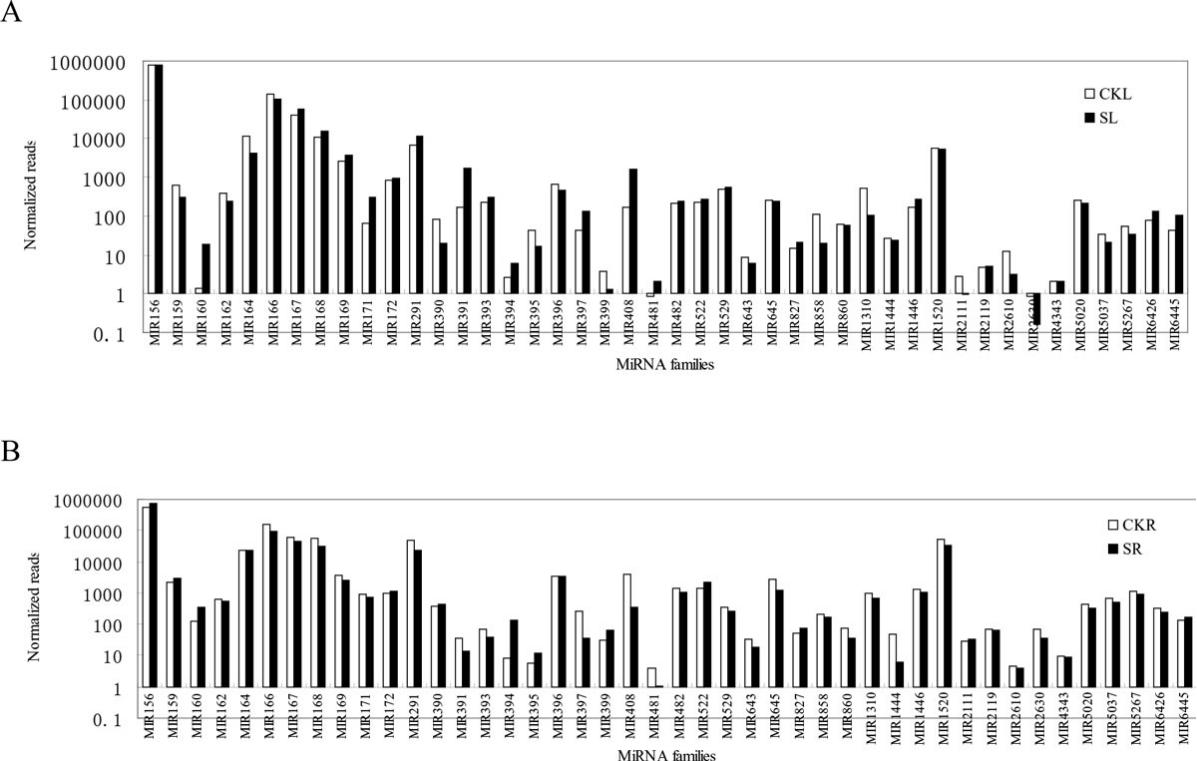
**Differential expression of conserved miRNA families in *Populus euphratica***. (A) Libraries constructed from salt-treated and control leaf tissue. (B) Libraries constructed from salt-treated and control root tissue.

To identify conserved miRNAs in *P. euphratica*, sequenced reads were searched by alignment against the known miRNA database in miRBase 20.0, allowing two mismatches; 10,202, 10,672, 10,471, and 11,037 sequences were identified in the 3dSL, 3dSR, 3dCKL, and 3dCKR libraries, respectively. The sequences with low expression levels (<5 reads) were removed, and the remaining reads were screened to find the characteristic hairpin structures in the *Populus *genome. Finally, we obtained 164 conserved miRNAs belonging to 44 families. Moreover, 59 miRNA* sequences were identified; these complementary sequence were considered direct evidence for the actual miRNAs [40]. In both the leaf and root libraries, the family of peu-miR156 was the most abundant, comprising about 60-70% of the total conserved miRNA reads. Moreover, the amount of peu-miR156 in leaves remained almost the same regardless of salt- or control-treatment; however, peu-miR156 in roots increased from 57.4% to 72.4% under the salt treatment (Figure 3). This result shows that a greater number of peu-miR156 members respond to salt stress in roots than in leaves. Peu-miR166 and peu-miR167 are the second-most enriched miRNA family (approximately 10% in these libraries) (Figure 3) and showed no obvious changes in salt stress. Meanwhile, some miRNA families were moderately represented in *P. euphratica*, such as peu-miR167, peu-miR169, peu-miR172, peu-miR827, peu-miR2119, and peu-miR5020. Among the conserved miRNAs families, the expressions of peu-miR393, peu-miR645, peu-miR860, and peu-miR1444 decreased significantly under salt stress in roots, but not in leaves. Peu-miR408 significantly increased in leaves (fold-change = 3.3) and decreased in roots (fold-change = -3.60). For peu-miR160 and peu-miR391, tenfold more reads were identified in 3dSL than in 3dCKL, and peu-miR394 was identified 17 times more in 3dSR than in 3dCKR.

### Identification of novel miRNAs in *P. euphratica*

According to the new annotation criteria of novel miRNAs [40], the detection of miRNA*s is robust proof for the existence of their miRNAs. In this study, we found 136 miRNA sequences in the leaf and 128 miRNAs in the root (Additional files 1 and 2). These sequences have hairpin structures and miRNA*s. The average precursor length was 160 nt in the leaf and 151 nt in the root. The minimal folding free energy varied from -198.7 to -19.1 kcal/mol, with an average of -54.36 kcal/mol in the leaf. In comparison, the minimal folding free energy varied from -136.2 to -19.42 kcal/mol, with an average of -53.01 kcal/mol in the root. In both the leaf and root, most new miRNAs were 21 nt long and had guanine (G) as their first nucleotide. More than half of the new miRNAs were located in the 5′ arm of the stem-loop. The novel miRNAs had different expression levels; some had high expression levels, such as Peu-sM31 in leaves and Peu-sM49 in roots, with normalized reads close to 1,000,000. To identify possible homologs with existing miRNAs, we searched these novel miRNAs against plant miRNAs in the miRBase database, and found 20 homologs in *P. trichocarpa *and 15 homologs in *A. thaliana *and *Glycine max*. Several novel miRNAs were identified in only one of the libraries, probably because the sequencing depth provided insufficient coverage of all the miRNAs; alternatively, the expression of some miRNAs might have been turned on or turned off suddenly by salt stress.

### Identification of salt-responsive miRNAs

**Figure 4:**
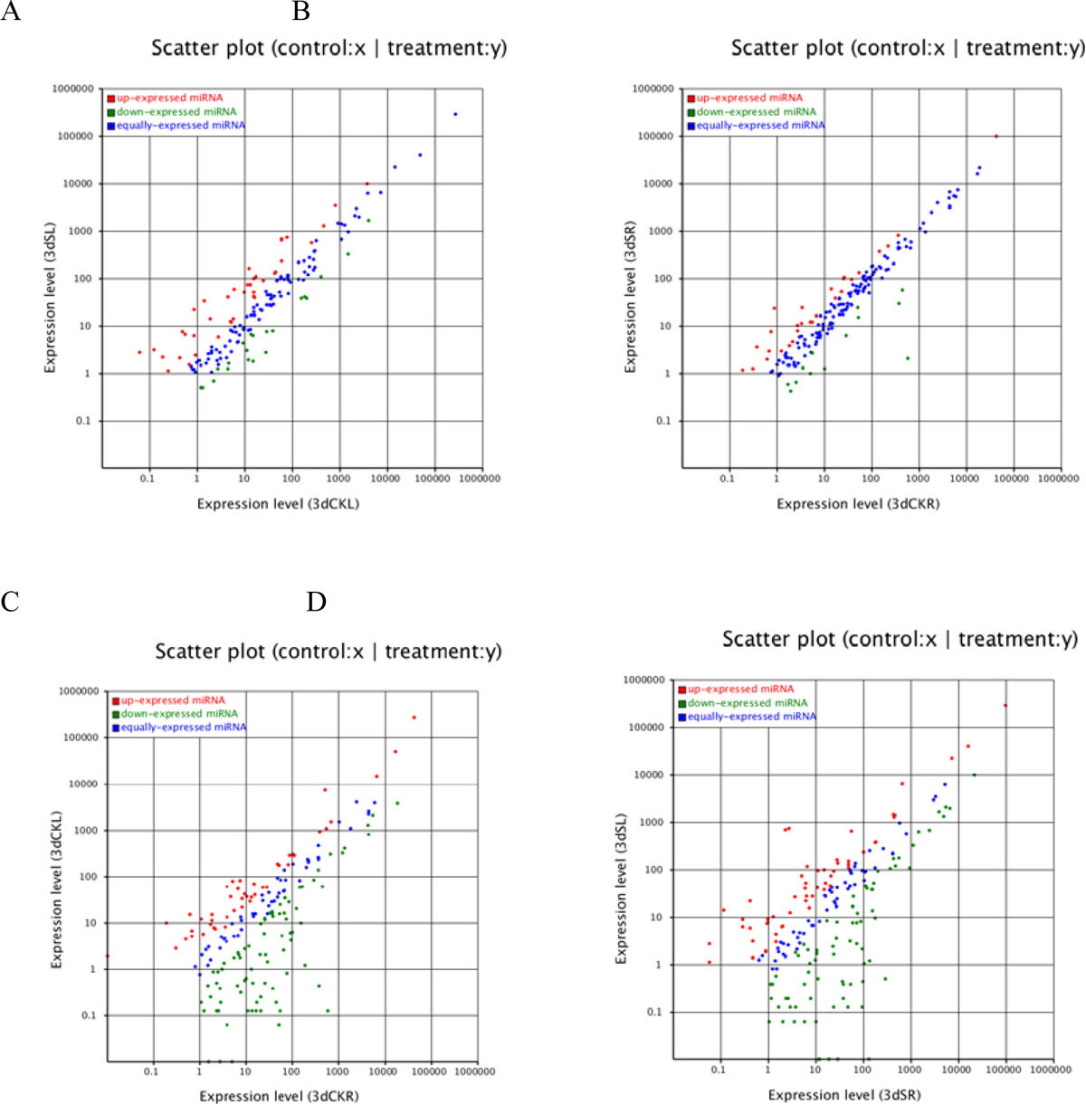
**Expression differences in conserved miRNAs between libraries constructed from salt-treated and untreated tissues**. (A) The salt-treated leaf (3dSL) and control leaf (3dCKL) libraries. (B) The salt-treated root (3dSR) and control root (3dSR) libraries. (C) The control root (3dCKR) and control leaf (3dCKL) libraries. (D) The salt-treated leaf (3dSL) and salt-treated root (3dSR) libraries. Each point in the figure represents a miRNA; the *x*- and *y*-axes represent the miRNA expression levels in the two samples; red represents miRNAs with ratios >2; blue represents miRNAs with ratios ≥½ and ≤2; green represents miRNAs with ratios <½; ratios are the normalized expression in the salt-treated sample:normalized expression in the control sample.

To identify the salt-responsive miRNAs among the deep sequencing reads, we performed differential expression analysis of miRNAs between two libraries. miRNAs with very low expression levels (normalized reads <1) were removed. miRNAs that showed a fold-change greater than 0.5 or less than -0.5 with *P*-values less than 0.05 were considered to be downregulated or upregulated, respectively. There are totally 157 miRNAs detected from 3dSL and 3d CKL libraries. Between the 3dSL and 3dCKL libraries, we identified 95 differentially regulated miRNAs belonging to 46 miRNAs families, including 39 downregulated and 56 upregulated miRNAs in response to salt stress (Additional file 3 and Figure 4A). The greatest change between the two libraries was in miR6424, which showed a change greater than fivefold. In addition, expression differences between the 3dSL and 3dSR libraries revealed 155 miRNAs from the totally 188 miRNAs belonging to 63 families, of which 84 were upregulated and 71 were downregulated from leaf to root tissue (Additional file 4 and Figure 4B). These significant changes in miRNAs expression between different libraries revealed that miRNAs are sensitive and responsive to salt stress; furthermore, a greater number of differentially expressed miRNAs play important roles in *Populus *root than leaf tissues.

### Prediction of target genes of novel miRNAs in *P. euphratica*

The target gene predictions were carried out as described previously [41,42] with default parameters. The functions of target genes were annotated using the GO and KEGG [44]. In root samples, 479 targets were identified for the novel miRNAs, with an average of six targets per miRNA (range: 1-43; Additional file 5). In comparison, 541 targets for novel miRNAs were identified in leaves, with an average of six targets per miRNA (range: 1-67; Additional file 6). The GO and KEGG databases predicted a wide distribution of target functions such as plant development, cell apoptosis, hormone regulation, cell defense, disease resistance, and the electron transfer chain. For example, according to the GO database, the most frequent gene targets were related to protein tyrosine phosphatase activity (GO term: 0004725). In contrast, the KEGG database inferred that most targets focused on various pathways including those involved in plant-pathogen interactions, the cell cycle, cytosolic DNA-sensing pathway, RNA polymerase, and Huntington's disease. The diverse functions of these target participants revealed that the novel miRNAs and their target genes play important roles in the developmental and regulatory processes in *P. euphratica*.

## Discussion

More than 954 million hectares of saline land areas exist worldwide, with about 10% of them distributed in China. Salinity stress has become a serious threat to plant growth and development that can not be ignored [47]. Many miRNAs and genes involved in the high-salinity stress response in plants have been identified [19,48]; however, little research has focused on the salt-responsive miRNAs in woody plants on a genome-wide scale. In this study, we constructed four small RNA cDNA libraries from the root or leaf tissues in *P. euphratica *plantlets treated with or without salt (300 mM NaCl, 3 days). By high-throughput sequencing, we identified 164 conserved miRNAs belonging to 44 families (136 novel miRNAs in leaf and 128 novel miRNAs in root), and provided a genome-wide expression profile of miRNAs in response to salt-stress conditions. Furthermore, we identified 479 target genes in root and 541 targets in leaf of novel miRNAs and annotated them by mapping to the GO and KEGG databases. These results strengthen the understanding of the roles of salt-responsive miRNAs and may provide an important reference for improving *Populus *resistance to salt stress.

**Figure 5:**
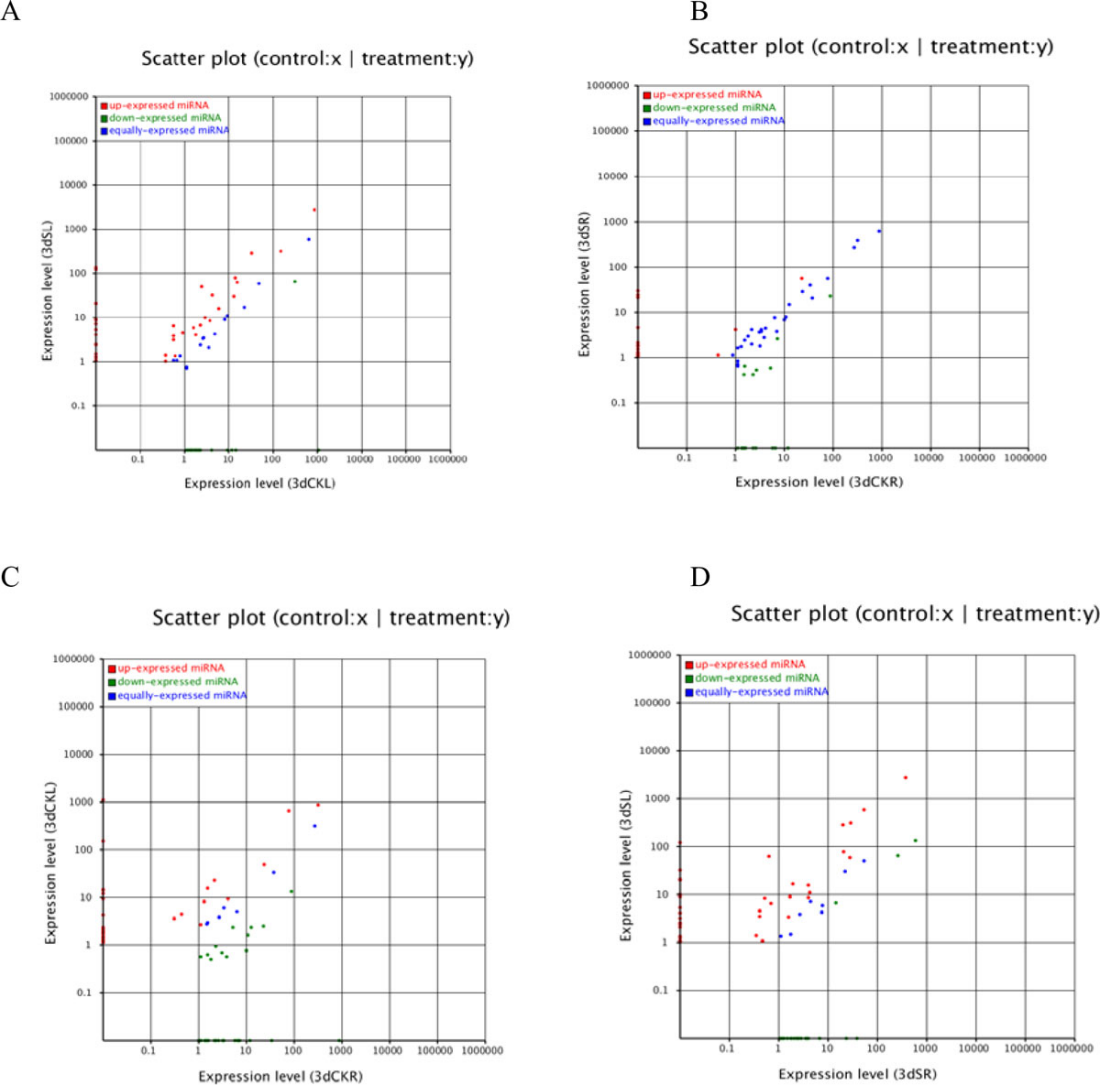
**Expression differences of novel miRNAs between libraries constructed from salt-treated and untreated tissues**. (A) The salt-treated leaf (3dSL) and control leaf (3dCKL) libraries. (B) The salt-treated root (3dSR) and control root (3dSR) libraries. (C) The control root (3dCKR) and control leaf (3dCKL) libraries. (D) The salt-treated leaf (3dSL) and salt-treated root (3dSR) libraries. Each point in the figure represents a miRNA; the *x*- and *y*-axes represent the miRNA expression levels in the two samples; red represents miRNAs with ratios >2; blue represents miRNAs with ratios ≥½ and ≤2; green represents miRNAs with ratios <½; ratios are the normalized expression in the salt-treated sample:normalized expression in the control sample.

In response to salt stress, 95 miRNAs belonging to 46 conserved miRNAs families were identified, containing 56 upregulated miRNAs and 39 downregulated miRNAs in the leaf. In a comparison of leaf and root tissues, we identified 155 miRNAs belonging to 63 families with significantly altered expression, including 84 upregulated and 71 downregulated miRNAs. An analysis of the changed expression of conserved miRNAs showed 21 miRNAs in the leaf (Additional file 3 and Figure 4A) and 14 miRNAs (Additional file 4 and Figure 4B) in the root that were significantly downregulated in response to salt stress. At the same time, 39 upregulated miRNAs were found in the leaf (Additional file 3 and Figure 4A) and 28 upregulated miRNAs (Additional file 4 and Figure 4B) were found in root tissues. In contrast, 60 miRNAs were downregulated and 54 were upregulated (Additional file 7 and Figure 4C) in the 3dCKR sample compared to the 3dCKL sample; furthermore, 77 were downregulated and 56 were upregulated (Additional file 8 and Figure 4D) in the 3dSR sample compared to the 3dSL sample. The expression of several miRNAs changed in response to salt stress; however, a greater difference in miRNA expression was detected when the root and leaf tissues were compared. A similar phenomenon was observed in the differential expression of the novel miRNAs: 16 upregulated and 38 downregulated predicted miRNAs were identified between the 3dCKL and 3dSL samples (Additional file 9 and Figure 5A), and 18 upregulated and 16 downregulated novel miRNAs were identified between the 3dCKR and 3dSR samples (Additional file 10 and Figure 5B). In contrast, 32 upregulated and 30 downregulated predicted miRNAs were found between the 3dCKL and 3dCKR samples (Additional file 11 and Figure 5C), and 23 upregulated and 41 downregulated novel miRNAs were found between the 3dSR and 3dSL samples (Additional file 12 and Figure 5D). The phenomenon suggested that the miRNA expression changes occurred not only in response to abiotic stress, but also in different tissues.

Peu-miR394a, which targets the gene encoding F-box proteins, was significantly upregulated in response to salt stress both in the leaf and root tissue; the same tendency was observed in ath-miR394 under high-salinity stress in *Arabidopsis *[7,22]. Another related miRNA, peu-miR393a, was upregulated in the leaf, but with weak expression changes in the root. The diverse expression trends observed for these two miRNAs in different species may indicate that F-box proteins play varied roles under salt-stress conditions in different regulated pathways [49-51]. Figure 3 shows that peu-miR160 expression was significantly upregulated in both tissues, in contrast to the findings in *Populus tomentosa*. MiR160 target genes encoded B3 DNA-binding domain proteins and auxin response factor (ARF). ARF affects various facets of plant growth and development and response to environmental changes [52-54]. Plant auxins act as signals for cell division, elongation, or differentiation and play important roles in lateral root formation, apical dominance, and tropisms [55]. In *Vigna*, the expression of vun-miR160a was clearly upregulated under salt conditions [56]; however, in maize, the expression of miR160a and miR160b was induced by 5 h of salt treatment but reduced by 24 h of salt treatment. The differentially altered expression in different species indicated that peu-miR160 might play a complex role in salt-stress resistance by affecting the auxin signaling pathways.

MiR168, miR169, and miR1444 were downregulated by salt shock in the root tissues. However, miR168 and miR169 were induced by salt in the leaf tissues, which was described in a previous study [26]. The induction of miR169 by salt stress was also reported in rice [23]. In *P. tomentosa*, the expression trends of miR168, miR169, and miR1444 were all restrained by salt stress [57]. MiR168 controls the Argonaute 1 (*AGO1*) gene and acts as a miRNA pathway regulator [27]. The altered expression of miR168 in salt-treated plants suggests that *AGO1 *may play important roles in response to salt stress. The CCAAT-binding transcription factors in *Arabidopsis *[58] and rice [23] are encoded by the target gene of miR169, which has been reported to play an important regulatory role in the response to salt stress. Polyphenol oxidase (*ppo*) genes, the targets of miR1444a, were found to be involved in the resistance of abiotic stress in plants. Studies have reported that Ptc-miR1444 might be involved in stress resistance in *P. trichocarpa *through the cleavage of *ppo *genes and disease resistance protein genes [27].

Peu-miR398 was significantly upregulated in the leaf under salt stress (Additional file 3) and downregulated in the root in response to salt. The expression level of miR398b was restrained after 9-12 h of salt treatment in *Populus cathayana*, whereas increased miR398 expression was observed in *P. tremula *[8]. The target gene of miR398b encodes copper/zinc superoxide dismutase (SODC), which showed an opposite expression pattern to miR398b [59]. Peu-miR395 expression decreased during salt stress, whereas miR395 was continuously induced under salt stress in *P. tremula *and maize [48]. MiR395 target genes encode adenosine phosphosulfate (APS) and Kelch motif proteins. APS and pyrophosphate anion (P_2_O_7_^4-^) form ATP [60]. MiR396 greatly impacts plant leaf growth and development by repressing growth-regulating factor (GRF) transcription factors [61]. The zma-miR396 family, which targets genes encoding cytochrome oxidase subunit I, were found to be downregulated under salt conditions in maize [48]. In *P. cathayana*, miR396f was downregulated under salt stress, and the target genes encoding GRF were induced [59]. MiR396 in *Arabidopsis *was significantly upregulated in response to abiotic stresses including salt treatment [7]. The expression of the newly found peu-miR396b was upregulated in response to salt stress in the leaf with almost no expression changes in the root. This result is in accord with previous research[61]. Peu-miR396b was predicted to target several genes including those encoding protein tyrosine kinases, zinc finger proteins, and various other protein kinases. In rice and *Arabidopsis*, miR396c was induced by salt stress [62]. These findings suggest that the upregulation of miR396 increased the expression of its targeted genes to help plants adapt to saline environments.

In the identification of salt-responsive miRNAs, miRNAs with differential expression were identified with a nominal threshold of p-value<0.05 and fold_change>1 or <-1. No multiple testing adjustments were applied, such as FDR. The False Discovery Rate (FDR) of a set of predictions is the expected percent of false ones in the set of predictions. If the algorithm returns 100 genes with a false discovery rate of 0.3 then we should expect 70 of them to be correct. In the work, because of the small size of the libraries, we only used fold_change to filter the false positives with p-value<0.05. In the future work, we should increase the sample size, and use popular multiple testing adjustments for reasons of credibility.

## Conclusions

We constructed four small RNA cDNAs libraries from the root or leaf of salt-treated and pure water-treated *P. euphratica *plantlets. Genome-wide high-throughput sequencing was employed to identify and analyze salt-induced miRNAs in woody plants. We identified most of the known miRNAs in *Populus *and several conserved miRNAs not found previously in other *Populus *species [45,63]. Several novel miRNAs and miRNAs* were identified, and the presence of Dicer-like (DCL)-processed precursors was revealed, which are characteristics of bona fide miRNAs [40]. Furthermore, the targets of the novel miRNAs were predicted and functionally annotated. The predicted genes are involved in a broad range of functions in response to salt stress including signal transduction, transcriptional regulation, and energy metabolism. Through high-throughput sequencing, these findings provide solid evidence that miRNAs exist in *Populus*; moreover, they are distributed widely and differentially expressed under different salt conditions and different tissues. miRNAs in the root are more sensitive than those in the leaf in response to salt stress. The discovery and characterization of these miRNAs will help uncover the molecular mechanisms of abiotic stress resistance and elucidate new members of these pathways in *Populus*.

## Competing interests

The corresponding author declares that there are no competing interests.

## Authors' contributions

RLW designed the study, TZ prepared the cDNA libraries for miRNA sequencing, JNS analyzed the data and performed the bioinformatic analyses, JNS and RLW drafted the manuscript, and all authors contributed to editing the final version. All authors have read and approved the final manuscript.

## Funding

Publication costs for this article came from Fundamental Research Funds for the Central Universities (TD2012-04), the Beijing Forestry University Young Scientist Fund (No. BLX2011007), the Research Fund for the Doctoral Program of Higher Education of China (20120014120011), Special Fund for Forest Scientific Research in the Public Welfare (201404102), NSF/IOS-0923975, Changjiang Scholars Award and "Thousand-person Plan" Award.

## Supplementary Material

Additional file 1 Novel microRNAs (miRNAs) identified in libraries constructed from the leaves of *Populus euphratica *that were treated with (3dSL) or without (control, 3dCKL) salt. Click here for file

Additional file 2 Novel miRNAs identified in libraries constructed from the roots of *Populus euphratica *that were treated with (3dSR) or without (control, 3dCKR) salt. Click here for file

Additional file 3 Significant expression changes in conserved *Populus euphratica *miRNAs between libraries that were constructed from the leaves of salt-treated (3dSL) or control-treated (3dCKL) plants. Click here for file

Additional file 4 Significant expression changes in conserved *Populus euphratica *miRNAs between libraries that were constructed from the roots of salt-treated (3dSR) and control-treated (3dCKR) plants. Click here for file

Additional file 5 **Summary of target genes of novel miRNAs from the root tissue**. Click here for file

Additional file 6 **Summary of target genes of novel miRNAs from the leaf tissue**. Click here for file

Additional file 7 Significant expression changes in conserved *Populus euphratica *miRNAs between the control leaf (3dCKL) and control root (3dCKR) libraries. Click here for file

Additional file 8 Significant expression changes in conserved miRNAs from salt-treated *Populus euphratica *in the leaf (3dSL) and root (3dSR) libraries. Click here for file

Additional file 9 Significant expression changes in novel miRNAs in the leaves of salt-treated *Populus euphratica *(3dSL) and control-treated (3dCKL) libraries. Click here for file

Additional file 10 Significant expression changes in novel miRNAs in the roots of salt-treated *Populus euphratica *(3dSR) and control-treated (3dCKR) libraries. Click here for file

Additional file 11 Significant expression changes in novel miRNAs in control-treated *Populus euphratica *leaf (3dCKL) and root (3dCKR) libraries. Click here for file

Additional file 12 Significant expression changes in novel miRNAs in the leaves of salt-treated *Populus euphratica *(3dSL) and untreated root (3dSR) libraries. Click here for file

## Notes

This article has been published as part of *BMC Genetics *Volume 15 Supplement 1, 2014: Selected articles from the International Symposium on Quantitative Genetics and Genomics of Woody Plants. The full contents of the supplement are available online at http://www.biomedcentral.com/bmcgenet/supplements/15/S1.
