# Supplementary material for: Genome-wide analysis of salt-responsive and novel microRNAs in Populus euphratica by deep sequencing
Source: BMC Genet. 2014 Jun 20;15(Suppl 1):S6. doi: 10.1186/1471-2156-15-S1-S6 (PMC4118626; doi:10.1186/1471-2156-15-S1-S6)
Supplement: Additional file 1 — Novel microRNAs (miRNAs) identified in libraries constructed from the leaves of Populus euphratica that were treated with (3dSL) or without (control, 3dCKL) salt. [file 1471-2156-15-S1-S6-S1.doc]

Additional file 1 - Novel miRNAs identified in *P. euphratica* treated (3dSL) with salt and control (3dCKL) libraries in leaf tissue.

| miRNA | MiRNA sequence(5’-3’) | L(nt) | Arm | Precursor | Location | MFE | MiRNA* sequence(5’-3’) | Fold change |
| --- | --- | --- | --- | --- | --- | --- | --- | --- |
|  |  |  |  | Length(nt) |  |  |  | S/C |
| Peu-sM1 | ATATTGTTCGCTTTGGGCCTT | 21 | 5p | 360 | Chr01:18490861:18491220:+ | -93.51 | * | 0.52 |
| Peu-sM2 | GCGTGCGAGGAGCCAAGCATA | 21 | 5p | 333 | Chr01:47432780:47433112:+ | -79.1 | * | 3.56 |
| Peu-sM3 | CAGCCAAGGATGACTTGCCGA | 21 | 5p | 168 | Chr01:80673:80840:- | -58.2 | * | 0.31 |
| Peu-sM4 | GGAATGTTGTCTGGCTCGAGG | 21 | 5p | 161 | Chr01:6622528:6622688:- | -56.5 | * | 4.32 |
| Peu-sM5 | TTCCATGGAATAGGCAGTGATG | 22 | 5p | 108 | Chr01:21311322:21311429:- | -50.6 | TAACACTGTTATTCCATGGAAGA | 0.45 |
| Peu-sM6 | TTCCATGGAATAGGCAGTGATG | 22 | 5p | 108 | Chr01:21315965:21316072:- | -49.1 | TAACACTGTTATTCCATGGAAGA | 0.45 |
| Peu-sM7 | TTCCATGGAATAGGCAGTGATG | 22 | 5p | 108 | Chr01:21319239:21319346:- | -49.1 | TAACACTGTTATTCCATGGAAGA | 0.45 |
| Peu-sM8 | TTCCATGGAATAGGCAGTGATG | 22 | 5p | 108 | Chr01:21322511:21322618:- | -49.1 | TAACACTGTTATTCCATGGAAGA | 0.45 |
| Peu-sM9 | TTCCATGGAATAGGCAGTGATG | 22 | 5p | 108 | Chr01:21325783:21325890:- | -49.1 | TAACACTGTTATTCCATGGAAGA | 0.45 |
| Peu-sM10 | TTTGGTGTTGTTGGATTCAT | 20 | 5p | 285 | Chr01:27237470:27237754:- | -136.2 | * | 0.40 |
| Peu-sM11 | TGTGTTCTCAGGTCGCCCCTG | 21 | 3p | 86 | Chr01:28030196:28030281:- | -39.2 | * | 2.52 |
| Peu-sM12 | AATCGTAATCATGGCTTAGGC | 21 | 3p | 202 | Chr02:5474406:5474607:+ | -84.7 | CTAAGCCGTGATTACGATTGA | 1.09 |
| Peu-sM13 | GGAATGTTGTCTGGCTCGAGG | 21 | 5p | 131 | Chr02:13561874:13562004:+ | -57.8 | * | 4.31 |
| Peu-sM14 | TTATAACCAAGACTAATGCAT | 21 | 5p | 135 | Chr02:13939358:13939492:+ | -34.32 | * | 0.66 |
| Peu-sM15 | TCATGCTTTAGAGATTGCTGG | 21 | 5p | 235 | Chr02:18085015:18085249:+ | -54.8 | * | 0.81 |
| Peu-sM16 | TGGATCCCGCCTTGCATCAAC | 21 | 3p | 174 | Chr03:6270772:6270945:+ | -75.2 | TGGTGCAGGTCGGGAACTGA | 2.31 |
| Peu-sM17 | TTGCATGCATGAACTTGAAAT | 21 | 3p | 209 | Chr04:21244617:21244825:+ | -82.24 | * | 0.32 |
| Peu-sM18 | GTGGGCGTGCCGGAGTGGTTA | 21 | 5p | 78 | Chr04:7552668:7552745:- | -27.9 | ATCATGTGGGCTTTGCCCGCGC | 0.11 |
| Peu-sM19 | TTTGGAAGTGTAGTTGCGGTT | 21 | 3p | 344 | Chr04:23199383:23199726:- | -72.36 | * | 0.52 |
| Peu-sM20 | TGGATCCCGCCTTGCATCAAC | 21 | 3p | 150 | Chr04:24020968:24021117:- | -69.4 | TGGTGCAGGTCGGGAACTGA | 2.32 |
| Peu-sM21 | TTCGGGAAACTCATTCGGTAT | 21 | 3p | 157 | Chr05:8376058:8376214:- | -55.5 | * | 0.57 |
| Peu-sM22 | TGAGGATTAGGAAAAGAAGAC | 21 | 5p | 149 | Chr05:14820448:14820596:- | -28.2 | * | 0.75 |
| Peu-sM23 | GCATGAGGGGAGTCGAGCAGG | 21 | 3p | 106 | Chr06:159790:159895:+ | -60.7 | * | 2.04 |
| Peu-sM24 | TATGATGGCTCGTGATCTTCA | 21 | 3p | 123 | Chr06:6946106:6946228:- | -78.4 | * | 0.60 |
| Peu-sM25 | ATTGTATTACTTATTTTTACTA | 22 | 5p | 203 | Chr06:10873319:10873521:- | -34.2 | * | 1.40 |
| Peu-sM26 | TCATGCTTTAGAGATTGCTGG | 21 | 5p | 235 | Chr06:16296452:16296686:- | -54.8 | * | 0.81 |
| Peu-sM27 | TTTGAAACAAGAGGGACTATT | 21 | 3p | 166 | Chr06:21595884:21596049:- | -63.75 | * | 0.47 |
| Peu-sM28 | GACAGAATGAGAAGTGAGCA | 20 | 5p | 108 | Chr06:26631518:26631625:- | -52.41 | * | 0.89 |
| Peu-sM29 | GGAATGTTGTCTGGCTCGAGG | 21 | 5p | 150 | Chr07:12409358:12409507:+ | -67.54 | * | 4.38 |
| Peu-sM30 | TTTGTTGATAGTCATCTAGT | 20 | 5p | 77 | Chr07:15484819:15484895:- | -29.4 | * | 1.92 |
| Peu-sM31 | AGATGGGAGAGTATGCAAGAAG | 22 | 5p | 109 | Chr08:8165930:8166038:+ | -47.1 | TCTTGCCTACTCCTCCCATTCC | 1.65 |
| Peu-sM32 | GTGGGCGTGCCGGAGTGGTTA | 21 | 5p | 75 | Chr08:8574800:8574874:+ | -32.3 | * | 0.11 |
| Peu-sM33 | TTTCCTCCACATTCGGTCAAT | 21 | 3p | 80 | Chr08:9457705:9457784:- | -34 | TGACCGAATATGGATGAAAAG | 1.16 |
| Peu-sM34 | TCTTGATCAATGGCCATTGTA | 21 | 5p | 138 | Chr08:14051169:14051306:- | -51.8 | CAGTGCCCATTGATTAAGATG | 0.66 |
| Peu-sM35 | TTCCATGGAATAGGCAGTGATG | 22 | 5p | 107 | Chr08:19062360:19062466:- | -48.2 | * | 0.45 |
| Peu-sM36 | GCGGCAGCATCAAGATTCACA | 21 | 5p | 130 | Chr09:4423818:4423947:+ | -58.2 | * | 10.61 |
| Peu-sM37 | ATTCGGTCAACGTTCGAGTGA | 21 | 3p | 100 | Chr10:12167986:12168085:+ | -36.8 | CCTCGAATATTGATCGAATAT | 0.38 |
| Peu-sM38 | GCAGCATCATCAAGATTCACA | 21 | 5p | 125 | Chr10:20940509:20940633:- | -54.5 | * | 7.93 |
| Peu-sM39 | GCGTGCGAGGAGCCAAGCATA | 21 | 3p | 117 | Chr11:17249459:17249575:- | -59.2 | * | 3.56 |
| Peu-sM40 | TGTTGGGATGGCTCAATCATG | 21 | 5p | 96 | Chr12:12562848:12562943:+ | -44.2 | * | 1.86 |
| Peu-sM41 | TGAAGACGAGCCGAATCAATA | 21 | 5p | 248 | Chr12:1811437:1811684:- | -45.35 | * | 0.37 |
| Peu-sM42 | TGAAGACGAGCCGAATCAATA | 21 | 3p | 88 | Chr12:1815784:1815871:- | -38.3 | * | 0.37 |
| Peu-sM43 | ATTTTAGGAAGGGAATGAATA | 21 | 3p | 148 | Chr12:9109814:9109961:- | -53.3 | * | 1.38 |
| Peu-sM44 | TGAGGATTAGGAAAAGAAGAC | 21 | 5p | 149 | Chr13:14722820:14722968:+ | -28.2 | * | 0.75 |
| Peu-sM45 | TGAGGATTAGGAAAAGAAGAC | 21 | 5p | 148 | Chr13:14888716:14888863:+ | -28.2 | * | 0.75 |
| Peu-sM46 | AGCTGCCGACTCATTCATTCA | 21 | 5p | 112 | Chr13:9936780:9936891:- | -40.6 | * | 1.10 |
| Peu-sM47 | CCAGCTGGCAATGATTTTGAA | 21 | 5p | 83 | Chr13:10682835:10682917:- | -20.9 | * | 0.46 |
| Peu-sM48 | GGAATGTTGTCTGGCTCGAGG | 21 | 5p | 140 | Chr14:8105976:8106115:+ | -50.9 | * | 4.31 |
| Peu-sM49 | GGAATGTTGTCTGGCTCGAGG | 21 | 5p | 140 | Chr14:8119902:8120041:+ | -50.9 | * | 4.31 |
| Peu-sM50 | TCATGCTTTAGAGATTGCTGG | 21 | 5p | 235 | Chr14:15345069:15345303:+ | -54.6 | * | 0.81 |
| Peu-sM51 | TCATGCTTTAGAGATTGCTGG | 21 | 5p | 235 | Chr14:5160069:5160303:- | -56.2 | * | 0.81 |
| Peu-sM52 | TAAAAGTATGGAGGGAAAGTG | 21 | 3p | 171 | Chr14:13937467:13937637:- | -62.6 | * | 0.57 |
| Peu-sM53 | ATCTCCCTCAAAGGCTTCCTC | 21 | 5p | 87 | Chr15:12599979:12600065:+ | -43.4 | * | 1.05 |
| Peu-sM54 | GCATGAGGGGAGTCACGCAGG | 21 | 3p | 106 | Chr16:125051:125156:+ | -53.3 | * | 3.83 |
| Peu-sM55 | TTAAAATTCTAGGGCATCAG | 20 | 5p | 234 | Chr16:7224206:7224439:+ | -74.29 | * | 0.85 |
| Peu-sM56 | TAAAAGTATGGAGGGAAAGTG | 21 | 3p | 158 | Chr17:1459026:1459183:+ | -58.79 | CTTTCCCTTTATACTTTTATA | 0.60 |
| Peu-sM57 | TTTCTTGTCGCAGGAGAGATGG | 22 | 5p | 156 | Chr17:8830018:8830173:+ | -72.3 | * | 1.20 |
| Peu-sM58 | GTGGGCGTGCCGGAGTGGTTA | 21 | 5p | 76 | Chr17:13852154:13852229:+ | -28.6 | * | 0.11 |
| Peu-sM59 | CAGGCGGTCTCCTTGGCTAA | 20 | 5p | 240 | Chr17:5767071:5767310:- | -84.2 | AGCCAAGGATGACTTGCCTGCT | 1.79 |
| Peu-sM60 | TCTTGATCAATGGCCATTGTA | 21 | 5p | 140 | Chr17:14311113:14311252:- | -57 | CAGTGCCCATTGATTAAGATG | 0.65 |
| Peu-sM61 | TCATGCTTTAGAGATTGCTGG | 21 | 5p | 235 | Chr17:15953465:15953699:- | -57.7 | * | 0.81 |
| Peu-sM62 | GCTCATTTCTCTTTCTGTCACT | 22 | 5p | 223 | Chr18:1583173:1583395:- | -59.1 | * | 6.07 |
| Peu-sM63 | AGATATGGTAGAGGGGCGCA | 20 | 3p | 84 | Chr19:9697924:9698007:+ | -34.6 | * | 0.66 |
| Peu-sM64 | TTTGGAAGGAAGATTTGAAGT | 21 | 3p | 126 | Chr19:10067558:10067683:+ | -80.1 | * | 2.92 |
| Peu-sM65 | TGAGGTCAAGTCGTCGTCCCC | 21 | 3p | 253 | Chr19:14250805:14251057:+ | -104.5 | * | 0.47 |
| Peu-sM66 | TTTGCAAAGAAGGATTACTAG | 21 | 3p | 103 | Chr19:15292459:15292561:+ | -42.69 | * | 0.96 |
| Peu-sM67 | AGCTGCCGACTCATTCATTCA | 21 | 5p | 104 | Chr19:15787418:15787521:+ | -36 | * | 1.10 |
| Peu-sM68 | TCCTTCCATTAGATTCCGCAA | 21 | 3p | 116 | Chr19:3286160:3286275:- | -38.6 | GTGTAGGATCTAATGAAAGGA | 1.16 |
| Peu-sM69 | CAGCCAAGGATGACTTGCCGA | 21 | 5p | 105 | Chr19:3663036:3663140:- | -43.8 | * | 0.31 |
| Peu-sM70 | TTTGGAAGGAAGATTTGAAGT | 21 | 3p | 126 | Chr19:10067559:10067684:- | -70.92 | * | 2.92 |
| Peu-sM71 | TCATGCTTTAGAGATTGCTGG | 21 | 5p | 326 | scaffold_20:362067:362392:- | -84.8 | * | 0.81 |
| Peu-sM72 | TGCTGAAATCTTGAGATACGG | 21 | 3p | 84 | scaffold_30:207260:207343:- | -21.6 | * | 0.38 |
| Peu-sM73 | TATTATTGTAAACAAGCTGAC | 21 | 5p | 210 | Chr01:39575014:39575223:+ | -38.8 | * | x |
| Peu-sM74 | TTGTAAGGGAAGCCCACATGG | 21 | 3p | 145 | Chr01:2252365:2252509:- | -57.8 | AAGTGGACTTCCCTTACAATC | x |
| Peu-sM75 | TCAGGATTGTGACAAGAGGTA | 21 | 5p | 124 | Chr01:32266976:32267099:- | -25.95 | * | x |
| Peu-sM76 | GAAGTGGAGGAAGACGACTGGTT | 23 | 5p | 178 | Chr01:46654996:46655173:- | -62.4 | * | x |
| Peu-sM77 | TCCACACGAGATTTCTGACTCTT | 23 | 3p | 118 | Chr02:4499456:4499573:+ | -31 | * | x |
| Peu-sM78 | TAATTGTTGGATTTTGGTTGGTT | 23 | 5p | 89 | Chr02:18302302:18302390:+ | -19.1 | * | x |
| Peu-sM79 | TCCACTGGACATCCTGCAAGCC | 22 | 3p | 359 | Chr02:168741:169099:- | -109.7 | * | x |
| Peu-sM80 | TATAAACTGACCCGGACACCT | 21 | 3p | 121 | Chr03:6380203:6380323:+ | -27.8 | * | x |
| Peu-sM81 | TTGAGAGAGTGTGTTAGAGAA | 21 | 3p | 294 | Chr03:10592068:10592361:- | -53.3 | * | x |
| Peu-sM82 | TTGGAACTTTTTGGAGACGGA | 21 | 3p | 282 | Chr03:19503247:19503528:- | -82.9 | * | x |
| Peu-sM83 | TGGGCGGGAACATGCATGCATA | 22 | 5p | 114 | Chr04:1330520:1330633:+ | -23.8 | * | x |
| Peu-sM84 | CCATTGAAGAGAATCATTTGT | 21 | 5p | 103 | Chr04:22273498:22273600:+ | -54.4 | * | x |
| Peu-sM85 | CATGGATGAGAGAAGAGATGGGT | 23 | 5p | 77 | Chr04:13573183:13573259:- | -30.5 | * | x |
| Peu-sM86 | ACAATTGATTGTTGGATGAAT | 21 | 3p | 225 | Chr05:1082732:1082956:+ | -95.19 | * | x |
| Peu-sM87 | ACAATTGATTGTTGGATGAAT | 21 | 3p | 226 | Chr05:1370865:1371090:+ | -97.01 | * | x |
| Peu-sM88 | TGTTTTTGGAGTTTCGTAGGA | 21 | 5p | 348 | Chr05:1421538:1421885:+ | -198.7 | * | x |
| Peu-sM89 | TTGGGCAGTCTCCTTGGCTA | 20 | 3p | 113 | Chr05:6809980:6810092:+ | -43.62 | * | x |
| Peu-sM90 | TCCAGTGAGATTGTTGTGTGA | 21 | 5p | 111 | Chr05:1362747:1362857:- | -30.6 | * | x |
| Peu-sM91 | TTTTTGGAGTTTCGTAGGAAG | 21 | 5p | 344 | Chr05:1421539:1421882:- | -197.7 | * | x |
| Peu-sM92 | CAGCCAAGGATGACTTGCCGG | 21 | 5p | 121 | Chr06:27635764:27635884:+ | -52.26 | * | x |
| Peu-sM93 | GAGAGATAGATACAAGAAGAG | 21 | 5p | 136 | Chr06:332670:332805:- | -48.7 | * | x |
| Peu-sM94 | TATAAAGAGCAGCTTGTACAA | 21 | 3p | 209 | Chr06:4471439:4471647:- | -52.9 | * | x |
| Peu-sM95 | CAGCCAAGGATGACTTGCCGG | 21 | 5p | 114 | Chr06:5794389:5794502:- | -64.29 | * | x |
| Peu-sM96 | TGACCTGATAGTTCGAGGGAC | 21 | 3p | 126 | Chr06:8251086:8251211:- | -62.8 | * | x |
| Peu-sM97 | CAGTATGACGAATTCCTCACAT | 22 | 5p | 71 | Chr06:18217939:18218009:- | -22.7 | GAGGGGGAGATTTGTTGTGTGTGG | x |
| Peu-sM98 | TTTGGAAGTATGGTTGTGGTTGC | 23 | 5p | 337 | Chr07:9419744:9420080:+ | -52.8 | * | x |
| Peu-sM99 | AGATCCGTAGATAGCAAGTAG | 21 | 5p | 116 | Chr08:250581:250696:+ | -43.9 | ATGCTATCTACTGATCTGAAC | x |
| Peu-sM100 | CAGCCAAGGATGACTTGCCGG | 21 | 5p | 196 | Chr08:5569970:5570165:+ | -55.6 | GGCAAGCTGTCCTTGGCTATG | x |
| Peu-sM101 | AGATCAGTAGATAGCATGTAG | 21 | 5p | 92 | Chr08:250591:250682:- | -42.5 | * | x |
| Peu-sM102 | CAATGGACATTGTAAGGGTGA | 21 | 5p | 123 | Chr10:13692757:13692879:- | -45.54 | * | x |
| Peu-sM103 | GTCTGGGTGGTGTAGTTGGTTAT | 23 | 5p | 85 | Chr11:2193031:2193115:+ | -27 | CCCCGGTTCGAACCCGGGCTC | x |
| Peu-sM104 | AGGATATCAGAAAGAAGTTGC | 21 | 3p | 233 | Chr11:1086921:1087153:- | -44.2 | * | x |
| Peu-sM105 | TCAGATTAGGTTTATCGTTGG | 21 | 5p | 321 | Chr11:11606025:11606345:- | -67.85 | * | x |
| Peu-sM106 | GGAGGCAGCGGTTCATCGATC | 21 | 5p | 104 | Chr12:6004885:6004988:+ | -43.8 | * | x |
| Peu-sM107 | GCTATGAGATCTGAGGGCAT | 20 | 5p | 180 | Chr13:1151036:1151215:+ | -45.64 | * | x |
| Peu-sM108 | GGCAGGTTGTTCTTGGCTACA | 21 | 3p | 116 | Chr13:4599494:4599609:+ | -49.25 | CAGCCAAGGATGACTTGCCGA | x |
| Peu-sM109 | TTGAGAGAGTGTGTTAGAGAA | 21 | 5p | 126 | Chr13:14417763:14417888:- | -24.53 | * | x |
| Peu-sM110 | GTGGTTCGCTCGTTGGGATTG | 21 | 3p | 76 | Chr14:16457187:16457262:- | -30.9 | * | x |
| Peu-sM111 | GGAGGCAGCGGTTCATCGATC | 21 | 3p | 290 | Chr15:6734424:6734713:+ | -64.54 | * | x |
| Peu-sM112 | CGGAACTCGGGTAGAAAGGGG | 21 | 5p | 151 | Chr15:8519558:8519708:+ | -33.5 | * | x |
| Peu-sM113 | TGTTGGGATGGCTCAATCATA | 21 | 5p | 95 | Chr15:11798528:11798622:+ | -46.6 | * | x |
| Peu-sM114 | TGAATATGCTTGAGCTGTGTT | 21 | 5p | 83 | Chr15:4019227:4019309:- | -42 | * | x |
| Peu-sM115 | TAGGACATGTAGAGAACACGG | 21 | 3p | 92 | Chr15:5373491:5373582:- | -24.3 | * | x |
| Peu-sM116 | ATGGGGAAGACAGGCACATGA | 21 | 5p | 159 | Chr16:13268996:13269154:- | -57.1 | * | x |
| Peu-sM117 | GATGCTGGTGACTGGTTGGGCGG | 23 | 5p | 263 | Chr17:2141472:2141734:+ | -104.7 | * | x |
| Peu-sM118 | CAGCCAAGGATGACTTGCCGG | 21 | 3p | 92 | Chr17:10307043:10307134:+ | -26.2 | * | x |
| Peu-sM119 | GGCAGTCTCCTTGGCTAAGC | 20 | 3p | 152 | Chr17:5766964:5767115:- | -58.1 | ATAGCCAAGGATGACTTGCCTG | x |
| Peu-sM120 | CAGCCAAGGATGACTTGCCGG | 21 | 5p | 141 | Chr18:268208:268348:+ | -68.8 | GGCAAGCTGTCCTTGGCTAC | x |
| Peu-sM121 | GACAGAAAGAGAAATGAGCA | 20 | 3p | 225 | Chr18:1583172:1583396:+ | -55.1 | * | x |
| Peu-sM122 | TTTGGAAGTATGGTTGTGGTTGC | 23 | 5p | 314 | Chr18:8618332:8618645:+ | -77.74 | * | x |
| Peu-sM123 | TGCTGGTGGGTTCTTGGGCTAA | 22 | 5p | 99 | Chr18:14522214:14522312:+ | -31.1 | * | x |
| Peu-sM124 | CGAGCCAAGAATGACTTGTCG | 21 | 5p | 138 | Chr18:16370838:16370975:- | -59.9 | CAGGTCGTTCTTGGCTCAACT | x |
| Peu-sM125 | CAGCCAAGGATGACTTGCCGG | 21 | 5p | 109 | Chr18:16384496:16384604:- | -52.1 | * | x |
| Peu-sM126 | AGATCAGTAGATAGCATGTAG | 21 | 5p | 95 | Chr19:9492527:9492621:+ | -32.2 | * | x |
| Peu-sM127 | AGAAACTGGATTGATGAGGGC | 21 | 3p | 162 | Chr19:12766388:12766549:- | -42.3 | * | x |
| Peu-sM128 | AGAAACTGGATTGATGAGGGC | 21 | 3p | 162 | Chr19:12845529:12845690:- | -39.3 | * | x |
| Peu-sM129 | AGAAACTGGATTGATGAGGGC | 21 | 3p | 162 | Chr19:12859403:12859564:- | -47 | * | x |
| Peu-sM130 | TGACTTTGCAAAGATAGATTT | 21 | 3p | 93 | Chr19:15292464:15292556:- | -37.79 | * | x |
| Peu-sM131 | CAGCCAAGGATGACTTGCCGG | 21 | 5p | 109 | scaffold_127:4422:4530:- | -53.4 | * | x |
| Peu-sM132 | GGTCATGCTCTGACAGCCTCACT | 23 | 3p | 111 | scaffold_1722:735:845:- | -42.76 | * | x |
| Peu-sM133 | TGGGAGTTTGAATTCGGTGTT | 21 | 5p | 277 | scaffold_20:680419:680695:+ | -66.09 | * | x |
| Peu-sM134 | ACTTCTGGATTGTAACGGCAA | 21 | 3p | 206 | scaffold_20:472140:472345:- | -44.51 | * | x |
| Peu-sM135 | TTTTGCGACGAAGTCGTTCTT | 21 | 5p | 198 | scaffold_20:599362:599559:- | -39.2 | * | x |
| Peu-sM136 | CAAAATATCAAGTCGGCTCAT | 21 | 5p | 221 | scaffold_22:38126:38346:- | -52.6 | * | x |
