# Supplementary material for: Genome-wide analysis of salt-responsive and novel microRNAs in Populus euphratica by deep sequencing
Source: BMC Genet. 2014 Jun 20;15(Suppl 1):S6. doi: 10.1186/1471-2156-15-S1-S6 (PMC4118626; doi:10.1186/1471-2156-15-S1-S6)
Supplement: Additional file 2 — Novel miRNAs identified in libraries constructed from the roots of Populus euphratica that were treated with (3dSR) or without (control, 3dCKR) salt. [file 1471-2156-15-S1-S6-S2.doc]

Additional file 2 - Novel miRNAs identified in *P. euphratica* treated (3dSR) with salt and control (3dCKR) libraries in root tissue.

| miRNA | MiRNA sequence(5’-3’) | L(nt) | Arm | Precursor | Location | MFE | MiRNA* sequence(5’-3’) | Fold change | |
| --- | --- | --- | --- | --- | --- | --- | --- | --- | --- |
|  |  |  |  | Length(nt) |  |  |  | S/C |  |
| Peu-sM1 | CAGGAGGGATAGCGCCATGAG | 21 | 5p | 112 | Chr01:28885490:28885601:+ | -46.91 | * | 2.47 |  |
| Peu-sM2 | TATTATTGTAAACAAGCTGAC | 21 | 5p | 210 | Chr01:39575014:39575223:+ | -38.8 | * | 1.67 |  |
| Peu-sM3 | GGAATGTTGTCTGGCTCGAGG | 21 | 5p | 161 | Chr01:6622528:6622688:- | -56.5 | * | 0.62 |  |
| Peu-sM4 | TTCCATGGAATAGGCAGTGATG | 22 | 5p | 108 | Chr01:21311322:21311429:- | -50.6 | TAACACTGTTATTCCATGGAAGA | 1.31 |  |
| Peu-sM5 | TTCCATGGAATAGGCAGTGATG | 22 | 5p | 108 | Chr01:21315965:21316072:- | -49.1 | TAACACTGTTATTCCATGGAAGA | 1.31 |  |
| Peu-sM6 | TTCCATGGAATAGGCAGTGATG | 22 | 5p | 108 | Chr01:21319239:21319346:- | -49.1 | TAACACTGTTATTCCATGGAAGA | 1.31 |  |
| Peu-sM7 | TTCCATGGAATAGGCAGTGATG | 22 | 5p | 108 | Chr01:21322511:21322618:- | -49.1 | TAACACTGTTATTCCATGGAAGA | 1.31 |  |
| Peu-sM8 | TTCCATGGAATAGGCAGTGATG | 22 | 5p | 108 | Chr01:21325783:21325890:- | -49.1 | TAACACTGTTATTCCATGGAAGA | 1.31 |  |
| Peu-sM9 | TTTGGTGTTGTTGGATTCAT | 20 | 5p | 285 | Chr01:27237470:27237754:- | -136.2 | * | 1.27 |  |
| Peu-sM10 | TGTGTTCTCAGGTCGCCCCTG | 21 | 3p | 86 | Chr01:28030196:28030281:- | -39.2 | * | 0.20 |  |
| Peu-sM11 | TCGGAATAGATGTGAGGTAGT | 21 | 3p | 150 | Chr01:28360356:28360505:- | -41.9 | * | 2.06 |  |
| Peu-sM12 | GGAATGTTGTCTGGCTCGAGG | 21 | 5p | 131 | Chr02:13561874:13562004:+ | -57.8 | * | 0.63 |  |
| Peu-sM13 | TTATAACCAAGACTAATGCAT | 21 | 5p | 135 | Chr02:13939358:13939492:+ | -34.32 | * | 0.31 |  |
| Peu-sM14 | TCATGCTTTAGAGATTGCTGG | 21 | 5p | 235 | Chr02:18085015:18085249:+ | -54.8 | * | 0.94 |  |
| Peu-sM15 | AGAGATCTCGGATCGAATCGGT | 22 | 5p | 72 | Chr02:11442352:11442423:- | -44.1 | * | 0.91 |  |
| Peu-sM16 | TGGATCCCGCCTTGCATCAAC | 21 | 3p | 174 | Chr03:6270772:6270945:+ | -75.2 | TGGTGCAGGTCGGGAACTGA | 1.28 |  |
| Peu-sM17 | TGTCAAGACTGGAGCAGGTAA | 21 | 3p | 96 | Chr03:20171657:20171752:- | -32.2 | * | 0.14 |  |
| Peu-sM18 | TTATGCATTTTTGTCCCTCG | 20 | 3p | 124 | Chr04:2380200:2380323:+ | -63.1 | * | 0.73 |  |
| Peu-sM19 | GTGGGCGTGCCGGAGTGGTTA | 21 | 5p | 78 | Chr04:7552668:7552745:- | -27.9 | ATCATGTGGGCTTTGCCCGCGC | 1.07 |  |
| Peu-sM20 | TGGATCCCGCCTTGCATCAAC | 21 | 3p | 150 | Chr04:24020968:24021117:- | -69.4 | TGGTGCAGGTCGGGAACTGA | 1.29 |  |
| Peu-sM21 | TCGTGACAAAGGTGGTATCAG | 21 | 5p | 138 | Chr05:1760568:1760705:+ | -32.2 | * | 1.21 |  |
| Peu-sM22 | TTATGCATTTTTGTCCCTCG | 20 | 3p | 125 | Chr05:24651380:24651504:+ | -60.6 | * | 0.71 |  |
| Peu-sM23 | TCTTATGCGTTTTTGTCTCTC | 21 | 3p | 115 | Chr05:24651686:24651800:+ | -60.72 | GGGACAAAAATGGCATAAGAGA | 0.79 |  |
| Peu-sM24 | TCTTATGCGTTTTTGTCTCTC | 21 | 3p | 115 | Chr05:24655881:24655995:+ | -61.62 | GAGACAAAAATGGCATAAGAGA | 0.79 |  |
| Peu-sM25 | TTATGCATTTTTGTCCCTCG | 20 | 3p | 125 | Chr05:24668286:24668410:+ | -60 | * | 0.65 |  |
| Peu-sM26 | TTCGGGAAACTCATTCGGTAT | 21 | 3p | 157 | Chr05:8376058:8376214:- | -55.5 | * | 1.46 |  |
| Peu-sM27 | AGATAGATTAGTTTCCTCTTT | 21 | 3p | 145 | Chr05:24647713:24647857:- | -81.9 | * | 0.58 |  |
| Peu-sM28 | GCATGAGGGGAGTCGAGCAGG | 21 | 3p | 106 | Chr06:159790:159895:+ | -60.7 | * | 0.47 |  |
| Peu-sM29 | CAGCCAAGGATGACTTGCCGG | 21 | 5p | 121 | Chr06:27635764:27635884:+ | -52.26 | * | 0.62 |  |
| Peu-sM30 | GGCGGGTTGTCCTTGGCTAT | 20 | 3p | 134 | Chr06:5794379:5794512:- | -71.29 | CAGCCAAGGATGACTTGCCGG | 0.47 |  |
| Peu-sM31 | TATGATGGCTCGTGATCTTCA | 21 | 3p | 123 | Chr06:6946106:6946228:- | -78.4 | * | 1.19 |  |
| Peu-sM32 | TCATGCTTTAGAGATTGCTGG | 21 | 5p | 235 | Chr06:16296452:16296686:- | -54.8 | * | 0.94 |  |
| Peu-sM33 | CAGTATGACGAATTCCTCACAT | 22 | 5p | 71 | Chr06:18217939:18218009:- | -22.7 | * | 1.15 |  |
| Peu-sM34 | TTTGAAACAAGAGGGACTATT | 21 | 3p | 166 | Chr06:21595884:21596049:- | -63.75 | * | 0.77 |  |
| Peu-sM35 | CAGAATGAGAAGTGAGCACGCA | 22 | 5p | 97 | Chr06:26631527:26631623:- | -52.1 | TGTGCTCACTCTCTTCTGTC | 1.24 |  |
| Peu-sM36 | GGAATGTTGTCTGGCTCGAGG | 21 | 5p | 150 | Chr07:12409358:12409507:+ | -67.54 | * | 0.61 |  |
| Peu-sM37 | CAGCCAAGGATGACTTGCCGG | 21 | 5p | 196 | Chr08:5569970:5570165:+ | -55.6 | * | 0.62 |  |
| Peu-sM38 | AGATGGGAGAGTATGCAAGAAG | 22 | 5p | 109 | Chr08:8165930:8166038:+ | -47.1 | TCTTGCCTACTCCTCCCATTCC | 1.57 |  |
| Peu-sM39 | GTGGGCGTGCCGGAGTGGTTA | 21 | 5p | 75 | Chr08:8574800:8574874:+ | -32.3 | * | 1.07 |  |
| Peu-sM40 | TTTCCTCCACATTCGGTCAAT | 21 | 3p | 80 | Chr08:9457705:9457784:- | -34 | * | 0.22 |  |
| Peu-sM41 | TCTTGATCAATGGCCATTGTA | 21 | 5p | 138 | Chr08:14051169:14051306:- | -51.8 | CAGTGCCCATTGATTAAGATG | 1.35 |  |
| Peu-sM42 | TTCCATGGAATAGGCAGTGATG | 22 | 5p | 107 | Chr08:19062360:19062466:- | -48.2 | * | 1.34 |  |
| Peu-sM43 | CAACTATTGGATCTCTTTTCT | 21 | 3p | 328 | Chr09:2730554:2730881:+ | -87.8 | * | 0.53 |  |
| Peu-sM44 | GCGGCAGCATCAAGATTCACA | 21 | 5p | 130 | Chr09:4423818:4423947:+ | -58.2 | * | 2.70 |  |
| Peu-sM45 | CAGGAGGGATAGCGCCATGAG | 21 | 5p | 116 | Chr09:7498158:7498273:+ | -57.4 | * | 2.47 |  |
| Peu-sM46 | CTCAAGGAGTAATTAGTGACA | 21 | 5p | 235 | Chr09:7718374:7718608:+ | -66 | * | 1.77 |  |
| Peu-sM47 | TCAAGATCAGTCATCAAGCAT | 21 | 3p | 107 | Chr10:17358738:17358844:- | -53 | * | 1.17 |  |
| Peu-sM48 | GCAGCATCATCAAGATTCACA | 21 | 5p | 125 | Chr10:20940509:20940633:- | -54.5 | * | 4.57 |  |
| Peu-sM49 | GTCTGGGTGGTGTAGTTGGTTAT | 23 | 5p | 85 | Chr11:2193031:2193115:+ | -27 | CCCCGGTTCGAACCCGGGCTC | 0.78 |  |
| Peu-sM50 | CCAAAAGGCGTAGTCGATGGA | 21 | 5p | 77 | Chr11:7197856:7197932:- | -19.42 | * | 0.82 |  |
| Peu-sM51 | GCGTGCGAGGAGCCAAGCATA | 21 | 3p | 117 | Chr11:17249459:17249575:- | -59.2 | * | 0.79 |  |
| Peu-sM52 | TGTTGGGATGGCTCAATCATG | 21 | 5p | 96 | Chr12:12562848:12562943:+ | -44.2 | * | 0.80 |  |
| Peu-sM53 | ATTTTAGGAAGGGAATGAATA | 21 | 3p | 148 | Chr12:9109814:9109961:- | -53.3 | * | 1.31 |  |
| Peu-sM54 | AATGAAGTTTGATCCAAGATC | 21 | 5p | 315 | Chr13:4144284:4144598:+ | -84.65 | * | 1.29 |  |
| Peu-sM55 | CGATGGGGTCGGTCCATGGAT | 21 | 3p | 76 | Chr13:14791873:14791948:+ | -32.7 | * | 0.85 |  |
| Peu-sM56 | AGCTGCCGACTCATTCATTCA | 21 | 5p | 112 | Chr13:9936780:9936891:- | -40.6 | * | 2.88 |  |
| Peu-sM57 | GGAATGTTGTCTGGCTCGAGG | 21 | 5p | 140 | Chr14:8105976:8106115:+ | -50.9 | * | 0.63 |  |
| Peu-sM58 | GGAATGTTGTCTGGCTCGAGG | 21 | 5p | 140 | Chr14:8119902:8120041:+ | -50.9 | * | 0.63 |  |
| Peu-sM59 | AGGCAAATGATGGAAAAAAA | 20 | 3p | 236 | Chr14:15319439:15319674:+ | -47.61 | * | 1.70 |  |
| Peu-sM60 | TCATGCTTTAGAGATTGCTGG | 21 | 5p | 235 | Chr14:15345069:15345303:+ | -54.6 | * | 0.94 |  |
| Peu-sM61 | TCATGCTTTAGAGATTGCTGG | 21 | 5p | 235 | Chr14:5160069:5160303:- | -56.2 | * | 0.94 |  |
| Peu-sM62 | TTGGGATGGCTCAATCATAT | 20 | 5p | 92 | Chr15:11798530:11798621:+ | -45.2 | * | 1.29 |  |
| Peu-sM63 | ATCTCCCTCAAAGGCTTCCTC | 21 | 5p | 87 | Chr15:12599979:12600065:+ | -43.4 | * | 0.85 |  |
| Peu-sM64 | CAGCCAAGGATGACTTGCCGG | 21 | 3p | 92 | Chr17:10307043:10307134:+ | -26.2 | * | 0.62 |  |
| Peu-sM65 | GTGGGCGTGCCGGAGTGGTTA | 21 | 5p | 76 | Chr17:13852154:13852229:+ | -28.6 | * | 1.07 |  |
| Peu-sM66 | TCATGCTTTAGAGATTGCTGG | 21 | 5p | 235 | Chr17:15953465:15953699:- | -57.7 | * | 0.94 |  |
| Peu-sM67 | CAGCCAAGGATGACTTGCCGG | 21 | 5p | 121 | Chr18:268218:268338:+ | -57.4 | GGCAAGCTGTCCTTGGCTACA | 0.73 |  |
| Peu-sM68 | GCTCATTTCTCTTTCTGTCACT | 22 | 5p | 223 | Chr18:1583173:1583395:- | -59.1 | * | 0.71 |  |
| Peu-sM69 | CAGCCAAGAATGATTTGCCGG | 21 | 5p | 120 | Chr18:16381170:16381289:- | -52.1 | * | 1.79 |  |
| Peu-sM70 | AGATATGGTAGAGGGGCGCA | 20 | 3p | 84 | Chr19:9697924:9698007:+ | -34.6 | * | 1.59 |  |
| Peu-sM71 | AGCTGCCGACTCATTCATTCA | 21 | 5p | 104 | Chr19:15787418:15787521:+ | -36 | * | 2.88 |  |
| Peu-sM72 | TCCTTCCATTAGATTCCGCAA | 21 | 3p | 116 | Chr19:3286160:3286275:- | -38.6 | GTGTAGGATCTAATGAAAGGA | 0.30 |  |
| Peu-sM73 | AATGAAGTTTGATCCAAGATC | 21 | 5p | 324 | Chr19:4229730:4230053:- | -84.74 | * | 1.29 |  |
| Peu-sM74 | CAGCCAAGAATGATTTGCCGG | 21 | 5p | 120 | scaffold_127:1100:1219:- | -53.9 | * | 1.79 |  |
| Peu-sM75 | TCATGCTTTAGAGATTGCTGG | 21 | 5p | 326 | scaffold_20:362067:362392:- | -84.8 | * | 0.94 |  |
| Peu-sM76 | CAATTCAATAAGAGATCTCGG | 21 | 5p | 96 | scaffold_23:338151:338246:- | -33.6 | * | 0.58 |  |
| Peu-sM77 | TGCTGAAATCTTGAGATACGG | 21 | 3p | 84 | scaffold_30:207260:207343:- | -21.6 | * | 1.00 |  |
| Peu-sM78 | TGGAAATTTTGGAAGACTTGA | 21 | 5p | 94 | Chr01:1881696:1881789:+ | -22.3 | * | x |  |
| Peu-sM79 | TAACTGGTTTTAGAGTTCGGGT | 22 | 5p | 257 | Chr01:23155255:23155511:+ | -50.64 | * | x |  |
| Peu-sM80 | TTGTAAGGGAAGCCCACATGG | 21 | 3p | 145 | Chr01:2252365:2252509:- | -57.8 | AAGTGGACTTCCCTTACAATC | x |  |
| Peu-sM81 | TTGCAGTGCCTCGGAACTCCAA | 22 | 3p | 213 | Chr01:14215942:14216154:- | -66.3 | * | x |  |
| Peu-sM82 | CTAAGCCGTGATTACGATTGA | 21 | 5p | 180 | Chr02:5474416:5474595:+ | -84.7 | AATCGTAATCATGGCTTAGGC | x |  |
| Peu-sM83 | CTCGTGTGGGTTGTGTGGTGT | 21 | 3p | 195 | Chr02:14106459:14106653:- | -60 | * | x |  |
| Peu-sM84 | CTATTGATGGTGGAATTGGAT | 21 | 3p | 192 | Chr04:20518491:20518682:+ | -48.5 | * | x |  |
| Peu-sM85 | TGCCAAAGAAGATTTGCCCCG | 21 | 3p | 116 | Chr04:4855678:4855793:- | -47.66 | * | x |  |
| Peu-sM86 | TTTGGATATTTGTAGACGAGGA | 22 | 3p | 129 | Chr04:17452373:17452501:- | -22.9 | * | x |  |
| Peu-sM87 | TAGACTGCTGCATTGGGTTA | 20 | 5p | 81 | Chr05:1141987:1142067:+ | -30.7 | * | x |  |
| Peu-sM88 | AGGATGGGTGCTGGCTTAAGGT | 22 | 3p | 94 | Chr05:3503399:3503492:+ | -34.7 | * | x |  |
| Peu-sM89 | AGAGGGACTTAGTGAAAGGTAT | 22 | 5p | 155 | Chr05:19401462:19401616:+ | -54.3 | * | x |  |
| Peu-sM90 | TTATGCATTTTTGTCCCTCG | 20 | 3p | 125 | Chr05:24655571:24655695:+ | -62.3 | * | x |  |
| Peu-sM91 | AAGGGTTTCTTACAGAGTTTA | 21 | 5p | 136 | Chr05:25090695:25090830:+ | -57.84 | AGCTCTGTTGGTCTCTCTTTG | x |  |
| Peu-sM92 | CTATAAAGGAGGAACATAGTTGT | 23 | 3p | 195 | Chr05:3100668:3100862:- | -38.1 | * | x |  |
| Peu-sM93 | AGATGTTGGGTAATTCGTATGGA | 23 | 5p | 236 | Chr05:14541456:14541691:- | -82.2 | * | x |  |
| Peu-sM94 | GAGGAGGGATGGAGAGGGAG | 20 | 3p | 237 | Chr05:21896702:21896938:- | -66.4 | * | x |  |
| Peu-sM95 | ATTGTCACTACCTCTGAGGCAA | 22 | 5p | 347 | Chr06:4670685:4671031:- | -87.2 | * | x |  |
| Peu-sM96 | CGATGTTGGTGAGGTTCAATC | 21 | 5p | 107 | Chr06:4914093:4914199:- | -42.9 | * | x |  |
| Peu-sM97 | CGGCTTTCTTGAACTTGGCAC | 21 | 5p | 160 | Chr07:9144016:9144175:+ | -62.8 | * | x |  |
| Peu-sM98 | TGGGGATGTAGCTCAGATGG | 20 | 3p | 346 | Chr07:13932336:13932681:+ | -61.37 | * | x |  |
| Peu-sM99 | TTTGTTGATAGTCATCTAGT | 20 | 5p | 77 | Chr07:15484819:15484895:- | -29.4 | * | x |  |
| Peu-sM100 | CTGACCAACGAAACGAGCTTG | 21 | 5p | 119 | Chr08:896394:896512:+ | -37.2 | * | x |  |
| Peu-sM101 | TGGAGCACCATCAAGATTCAC | 21 | 5p | 142 | Chr08:3423593:3423734:+ | -56.2 | * | x |  |
| Peu-sM102 | TAGGAAACACGAGCGAAAAGG | 21 | 3p | 123 | Chr08:19423547:19423669:+ | -35 | * | x |  |
| Peu-sM103 | TTATGGAAGCTGGAAGTACGA | 21 | 5p | 263 | Chr09:990532:990794:+ | -56.34 | * | x |  |
| Peu-sM104 | TGTGTTCTCAGGTCGCCCCTG | 21 | 3p | 101 | Chr09:6766515:6766615:- | -46.5 | * | x |  |
| Peu-sM105 | TCATCTTGACTAGTAAGACGCGT | 23 | 5p | 269 | Chr10:4406876:4407144:- | -106.1 | * | x |  |
| Peu-sM106 | AGATATTGGTGCGGTTCAATC | 21 | 5p | 100 | Chr11:334851:334950:- | -53.3 | * | x |  |
| Peu-sM107 | GGCAGGTTGTTCTTGGCTAC | 20 | 3p | 118 | Chr13:4599492:4599609:+ | -50.25 | AGCCAAGGATGACTTGCCGA | x |  |
| Peu-sM108 | CATAAGGCGGTGGACATATTG | 21 | 5p | 265 | Chr13:13647587:13647851:+ | -73 | * | x |  |
| Peu-sM109 | CGTAGGAAACACGAGCGAAAAG | 22 | 3p | 122 | Chr14:589471:589592:+ | -28.1 | TGCTTGCTTGTGTTTGTTCCTTGTTT | x |  |
| Peu-sM110 | CATCTGCAGACTACTTGCCTT | 21 | 5p | 137 | Chr14:7860844:7860980:+ | -66.7 | AGGCAAGTATTCAGTAGATGT | x |  |
| Peu-sM111 | TTTCGGGAAGTGAAATTTGGA | 21 | 3p | 136 | Chr14:10400677:10400812:+ | -41.9 | * | x |  |
| Peu-sM112 | CAGGAATTGGCAGATTTCCTC | 21 | 3p | 85 | Chr14:11387631:11387715:- | -25.4 | * | x |  |
| Peu-sM113 | AGAGGGACTTAGTGAAAGGTAT | 22 | 5p | 155 | Chr15:4748303:4748457:+ | -48.1 | * | x |  |
| Peu-sM114 | CTTCGGGTAGCTGGCAGAAGG | 21 | 3p | 211 | Chr15:8488083:8488293:+ | -61.43 | * | x |  |
| Peu-sM115 | ATGGGGAAGACAGGCACATGA | 21 | 5p | 159 | Chr16:13268996:13269154:- | -57.1 | * | x |  |
| Peu-sM116 | TAATGGAGACTCGCTTTTGACC | 22 | 3p | 126 | Chr17:15343505:15343630:+ | -36.3 | * | x |  |
| Peu-sM117 | TCAGGCGGTCTCCTTGGCTAA | 21 | 5p | 245 | Chr17:5767067:5767311:- | -86.9 | * | x |  |
| Peu-sM118 | GACAGAAGACTAGAGAGCAC | 20 | 5p | 107 | Chr17:13418460:13418566:- | -51.6 | * | x |  |
| Peu-sM119 | TCTTGATCAATGGCCATTGTA | 21 | 5p | 140 | Chr17:14311113:14311252:- | -57 | * | x |  |
| Peu-sM120 | CAGCTTTCTTGAACTTCTTTG | 21 | 5p | 143 | Chr18:14916703:14916845:- | -54.4 | * | x |  |
| Peu-sM121 | CGAGCCAAGAATGACTTGTCG | 21 | 5p | 115 | Chr18:16370851:16370965:- | -57 | CCAGGTCGTTCTTGGCTCAAC | x |  |
| Peu-sM122 | TGAGGTCAAGTCGTCGTCCCC | 21 | 3p | 253 | Chr19:14250805:14251057:+ | -104.5 | * | x |  |
| Peu-sM123 | TGACTTTGCAAAGATAGATTT | 21 | 3p | 93 | Chr19:15292464:15292556:- | -37.79 | * | x |  |
| Peu-sM124 | AGGATGGGTGCTGGCTTAAGGT | 22 | 3p | 97 | scaffold_1458:1508:1604:+ | -37.7 | * | x |  |
| Peu-sM125 | CTCTGCCACAAATTTTGACGT | 21 | 3p | 192 | scaffold_207:52944:53135:+ | -48 | * | x |  |
| Peu-sM126 | AACTTCTGGATTGTAACGGCA | 21 | 3p | 204 | scaffold_20:472141:472344:- | -44.51 | * | x |  |
| Peu-sM127 | TTTGATAATTTGGCAGCTCTA | 21 | 3p | 156 | scaffold_30:63760:63915:- | -27.5 | * | x |  |
| Peu-sM128 | CTCGGGGGGGACGAATCGGAG | 21 | 3p | 239 | scaffold_45:55385:55623:- | -102.16 | * | x |  |
