# Supplementary material for: Genome-wide analysis of salt-responsive and novel microRNAs in Populus euphratica by deep sequencing
Source: BMC Genet. 2014 Jun 20;15(Suppl 1):S6. doi: 10.1186/1471-2156-15-S1-S6 (PMC4118626; doi:10.1186/1471-2156-15-S1-S6)
Supplement: Additional file 3 — Significant expression changes in conserved Populus euphratica miRNAs between libraries that were constructed from the leaves of salt-treated (3dSL) or control-treated (3dCKL) plants. [file 1471-2156-15-S1-S6-S3.doc]

Additional file 3 - Significantly expression changed of conserved miRNAs identified in *P. euphratica* between treated (3dSL) with salt and control (3dCKL) libraries in leaf tissue.

| pairwise | miR-name | 3dCKL-  expressed | 3dSL-  expressed | 3dCKL-std | 3dSL-std | fold-change(log2 3dSL/3dCKL) | p-value | sig-lable |
| --- | --- | --- | --- | --- | --- | --- | --- | --- |
| 3dCKL-3dSL | miR5301 | 450 | 43 | 28.0868 | 2.6894 | -3.38454 | 1.12E-86 | ** |
| 3dCKL-3dSL | miR6145e | 238 | 28 | 14.8548 | 1.7512 | -3.08451 | 7.85E-43 | ** |
| 3dCKL-3dSL | miR6457b | 191 | 30 | 11.9213 | 1.8763 | -2.66758 | 4.93E-30 | ** |
| 3dCKL-3dSL | miR6478 | 3222 | 605 | 201.1015 | 37.8389 | -2.40998 | 0 | ** |
| 3dCKL-3dSL | miR858 | 624 | 126 | 38.947 | 7.8805 | -2.30515 | 4.7E-80 | ** |
| 3dCKL-3dSL | miR2916 | 24026 | 5115 | 1499.586 | 319.9111 | -2.22882 | 0 | ** |
| 3dCKL-3dSL | miR1310 | 2921 | 651 | 182.3145 | 40.716 | -2.16276 | 0 | ** |
| 3dCKL-3dSL | miR845b-5p | 2512 | 608 | 156.7868 | 38.0266 | -2.04372 | 2.6E-272 | ** |
| 3dCKL-3dSL | miR390a | 467 | 120 | 29.1479 | 7.5052 | -1.95743 | 2.55E-49 | ** |
| 3dCKL-3dSL | miR6300 | 6515 | 1715 | 406.6345 | 107.2625 | -1.92259 | 0 | ** |
| 3dCKL-3dSL | miR3949 | 178 | 48 | 11.1099 | 3.0021 | -1.8878 | 6.73E-19 | ** |
| 3dCKL-3dSL | miR2610a | 71 | 20 | 4.4315 | 1.2509 | -1.82483 | 4.86E-08 | ** |
| 3dCKL-3dSL | miR5665 | 36 | 11 | 2.2469 | 0.688 | -1.70746 | 0.000228 | ** |
| 3dCKL-3dSL | miR5562-3p | 75 | 26 | 4.6811 | 1.6261 | -1.52543 | 7.84E-07 | ** |
| 3dCKL-3dSL | miR399f | 21 | 8 | 1.3107 | 0.5003 | -1.38947 | 0.016373 | * |
| 3dCKL-3dSL | miR164a | 65624 | 26391 | 4095.929 | 1650.591 | -1.31121 | 0 | ** |
| 3dCKL-3dSL | miR395a | 241 | 100 | 15.042 | 6.2544 | -1.26605 | 1.17E-14 | ** |
| 3dCKL-3dSL | miR3954 | 19 | 8 | 1.1859 | 0.5003 | -1.24512 | 0.036177 | * |
| 3dCKL-3dSL | miR6485 | 44 | 20 | 2.7463 | 1.2509 | -1.13452 | 0.002698 | ** |
| 3dCKL-3dSL | miR5298b | 149 | 68 | 9.2999 | 4.253 | -1.12873 | 3.11E-08 | ** |
| 3dCKL-3dSL | miR4995 | 220 | 105 | 13.7313 | 6.5671 | -1.06414 | 1.38E-10 | ** |
| 3dCKL-3dSL | miR394b-3p | 67 | 34 | 4.1818 | 2.1265 | -0.97564 | 0.001023 |  |
| 3dCKL-3dSL | miR165a-3p | 2791 | 1480 | 174.2006 | 92.5647 | -0.91222 | 2.49E-90 |  |
| 3dCKL-3dSL | miR165a | 4584 | 2432 | 286.1109 | 152.1063 | -0.91149 | 6.4E-147 |  |
| 3dCKL-3dSL | miR2089-3p | 32 | 17 | 1.9973 | 1.0632 | -0.90964 | 0.033438 |  |
| 3dCKL-3dSL | miR4348 | 45 | 24 | 2.8087 | 1.501 | -0.90398 | 0.011811 |  |
| 3dCKL-3dSL | miR780.2 | 84 | 45 | 5.2429 | 2.8145 | -0.89749 | 0.000597 |  |
| 3dCKL-3dSL | miR159a | 3463 | 1896 | 216.1435 | 118.5829 | -0.86609 | 2.1E-102 |  |
| 3dCKL-3dSL | miR403c-5p | 1350 | 758 | 84.2604 | 47.4081 | -0.82972 | 3.03E-38 |  |
| 3dCKL-3dSL | miR5671 | 4848 | 2741 | 302.5885 | 171.4323 | -0.81972 | 5.8E-130 |  |
| 3dCKL-3dSL | miR6441 | 17666 | 10672 | 1102.625 | 667.4665 | -0.72418 | 0 |  |
| 3dCKL-3dSL | miR5755 | 215 | 134 | 13.4192 | 8.3809 | -0.67912 | 0.000015 |  |
| 3dCKL-3dSL | miR4993 | 118 | 74 | 7.365 | 4.6282 | -0.67023 | 0.001548 |  |
| 3dCKL-3dSL | miR6425a-5p | 113 | 71 | 7.0529 | 4.4406 | -0.66746 | 0.002028 |  |
| 3dCKL-3dSL | miR2912a | 24462 | 15430 | 1526.799 | 965.0495 | -0.66183 | 0 |  |
| 3dCKL-3dSL | miR4414b | 50 | 32 | 3.1208 | 2.0014 | -0.64091 | 0.048584 |  |
| 3dCKL-3dSL | miR5037c | 193 | 129 | 12.0461 | 8.0681 | -0.57827 | 0.00038 |  |
| 3dCKL-3dSL | miR2604 | 320 | 216 | 19.9728 | 13.5094 | -0.56407 | 7.55E-06 |  |
| 3dCKL-3dSL | miR162a | 2172 | 1526 | 135.5656 | 95.4417 | -0.5063 | 3.45E-26 |  |
| 3dCKL-3dSL | miR393h | 1296 | 1882 | 80.89 | 117.7073 | 0.541171 | 1.06E-25 |  |
| 3dCKL-3dSL | miR1523a | 484 | 714 | 30.2089 | 44.6562 | 0.563887 | 2.11E-11 |  |
| 3dCKL-3dSL | miR6433-3p | 290 | 430 | 18.1004 | 26.8938 | 0.571252 | 1.46E-07 |  |
| 3dCKL-3dSL | miR5658 | 101 | 153 | 6.3039 | 9.5692 | 0.602154 | 0.001027 |  |
| 3dCKL-3dSL | miR5368 | 249 | 384 | 15.5414 | 24.0168 | 0.627927 | 6.4E-08 |  |
| 3dCKL-3dSL | miR169ac | 14925 | 23041 | 931.5455 | 1441.07 | 0.629442 | 0 |  |
| 3dCKL-3dSL | miR167h | 231342 | 359284 | 14439.24 | 22470.96 | 0.638067 | 0 |  |
| 3dCKL-3dSL | miR168a | 61813 | 97935 | 3858.065 | 6125.219 | 0.666884 | 0 |  |
| 3dCKL-3dSL | miR827 | 82 | 131 | 5.118 | 8.1932 | 0.678847 | 0.00073 |  |
| 3dCKL-3dSL | miR5646 | 2186 | 3512 | 136.4394 | 219.6535 | 0.686969 | 2.8E-70 |  |
| 3dCKL-3dSL | miR6474 | 922 | 1574 | 57.5467 | 98.4438 | 0.774567 | 1.23E-39 |  |
| 3dCKL-3dSL | miR6476 | 33 | 57 | 2.0597 | 3.565 | 0.791468 | 0.011141 |  |
| 3dCKL-3dSL | miR6426a | 459 | 835 | 28.6485 | 52.224 | 0.866253 | 4.55E-26 |  |
| 3dCKL-3dSL | miR1446a | 946 | 1733 | 59.0447 | 108.3883 | 0.87633 | 2.71E-53 |  |
| 3dCKL-3dSL | miR482a | 784 | 1465 | 48.9334 | 91.6265 | 0.904945 | 9.32E-48 |  |
| 3dCKL-3dSL | miR1448 | 759 | 1431 | 47.3731 | 89.5001 | 0.917821 | 9.02E-48 |  |
| 3dCKL-3dSL | miR171b-3p | 5067 | 9869 | 316.2574 | 617.2439 | 0.964741 | 0 |  |
| 3dCKL-3dSL | miR6427-5p | 45 | 92 | 2.8087 | 5.754 | 1.034663 | 0.000051 | ** |
| 3dCKL-3dSL | miR5725 | 11 | 24 | 0.6866 | 1.501 | 1.128382 | 0.02837 | * |
| 3dCKL-3dSL | miR472b | 4111 | 9110 | 256.5885 | 569.7732 | 1.150931 | 0 | ** |
| 3dCKL-3dSL | miR171e | 83 | 192 | 5.1805 | 12.0084 | 1.212881 | 2.62E-11 | ** |
| 3dCKL-3dSL | miR482c-3p | 262 | 627 | 16.3528 | 39.2149 | 1.261864 | 1.46E-35 | ** |
| 3dCKL-3dSL | miR5139 | 92 | 227 | 5.7422 | 14.1974 | 1.305951 | 1.56E-14 | ** |
| 3dCKL-3dSL | miR530b | 81 | 200 | 5.0556 | 12.5087 | 1.306978 | 5.44E-13 | ** |
| 3dCKL-3dSL | miR394a | 15 | 38 | 0.9362 | 2.3767 | 1.344071 | 0.001458 | ** |
| 3dCKL-3dSL | miR156a | 60513 | 158751 | 3776.926 | 9928.877 | 1.394418 | 0 | ** |
| 3dCKL-3dSL | miR6445a | 253 | 668 | 15.791 | 41.7792 | 1.403682 | 3.32E-44 | ** |
| 3dCKL-3dSL | miR396b-3p | 7336 | 20406 | 457.8773 | 1276.267 | 1.478897 | 0 | ** |
| 3dCKL-3dSL | miR168a-3p | 685 | 2044 | 42.7544 | 127.8394 | 1.580188 | 1.6E-156 | ** |
| 3dCKL-3dSL | miR774b-5p | 721 | 2209 | 45.0013 | 138.1591 | 1.618292 | 7.3E-175 | ** |
| 3dCKL-3dSL | miR397a | 252 | 823 | 15.7286 | 51.4735 | 1.71044 | 1.27E-71 | ** |
| 3dCKL-3dSL | miR475a-3p | 399 | 1435 | 24.9036 | 89.7502 | 1.849561 | 1.8E-137 | ** |
| 3dCKL-3dSL | miR167f-3p | 960 | 3651 | 59.9185 | 228.3471 | 1.930155 | 0 | ** |
| 3dCKL-3dSL | miR2911 | 13068 | 54536 | 815.6407 | 3410.884 | 2.06414 | 0 | ** |
| 3dCKL-3dSL | miR6447 | 4 | 18 | 0.2497 | 1.1258 | 2.172683 | 0.002558 | ** |
| 3dCKL-3dSL | miR6462c-5p | 7 | 33 | 0.4369 | 2.0639 | 2.239998 | 2.46E-05 | ** |
| 3dCKL-3dSL | miR477a-5p | 157 | 814 | 9.7992 | 50.9106 | 2.37723 | 5E-108 | ** |
| 3dCKL-3dSL | miR6433-5p | 214 | 1165 | 13.3568 | 72.8634 | 2.44762 | 3.7E-159 | ** |
| 3dCKL-3dSL | miR473a-3p | 186 | 1155 | 11.6092 | 72.238 | 2.637489 | 9.9E-172 | ** |
| 3dCKL-3dSL | miR479 | 280 | 1760 | 17.4762 | 110.0769 | 2.655048 | 1.3E-262 | ** |
| 3dCKL-3dSL | miR6453 | 253 | 1593 | 15.791 | 99.6321 | 2.657508 | 4.1E-238 | ** |
| 3dCKL-3dSL | miR6421-5p | 14 | 99 | 0.8738 | 6.1918 | 2.824984 | 3.19E-17 | ** |
| 3dCKL-3dSL | miR6427-3p | 30 | 225 | 1.8725 | 14.0723 | 2.909821 | 1.85E-38 | ** |
| 3dCKL-3dSL | miR157d-3p | 71 | 649 | 4.4315 | 40.5909 | 3.195289 | 3.1E-118 | ** |
| 3dCKL-3dSL | miR3627-5p | 1253 | 11819 | 78.2061 | 739.2042 | 3.24062 | 0 | ** |
| 3dCKL-3dSL | miR473a-5p | 97 | 939 | 6.0543 | 58.7285 | 3.278029 | 2.2E-174 | ** |
| 3dCKL-3dSL | miR408b | 961 | 10253 | 59.9809 | 641.2607 | 3.418336 | 0 | ** |
| 3dCKL-3dSL | miR391 | 970 | 10818 | 60.5427 | 676.5979 | 3.482274 | 0 | ** |
| 3dCKL-3dSL | miR393a-3p | 3 | 35 | 0.1872 | 2.189 | 3.54762 | 3.49E-08 | ** |
| 3dCKL-3dSL | miR169n-3p | 9 | 106 | 0.5617 | 6.6296 | 3.56105 | 1.81E-22 | ** |
| 3dCKL-3dSL | miR160b-3p | 198 | 2603 | 12.3582 | 162.8013 | 3.719572 | 0 | ** |
| 3dCKL-3dSL | miR160a | 8 | 119 | 0.4993 | 7.4427 | 3.897847 | 8.01E-27 | ** |
| 3dCKL-3dSL | miR477a-3p | 23 | 536 | 1.4355 | 33.5234 | 4.545543 | 1.3E-128 | ** |
| 3dCKL-3dSL | miR397b-3p | 2 | 49 | 0.1248 | 3.0646 | 4.618009 | 5.83E-13 | ** |
| 3dCKL-3dSL | miR398c-5p | 14 | 351 | 0.8738 | 21.9528 | 4.650958 | 6.75E-86 | ** |
| 3dCKL-3dSL | miR6424 | 1 | 43 | 0.0624 | 2.6894 | 5.429595 | 2.5E-12 | ** |
