# Supplementary material for: Genome-wide analysis of salt-responsive and novel microRNAs in Populus euphratica by deep sequencing
Source: BMC Genet. 2014 Jun 20;15(Suppl 1):S6. doi: 10.1186/1471-2156-15-S1-S6 (PMC4118626; doi:10.1186/1471-2156-15-S1-S6)
Supplement: Additional file 4 — Significant expression changes in conserved Populus euphratica miRNAs between libraries that were constructed from the roots of salt-treated (3dSR) and control-treated (3dCKR) plants. [file 1471-2156-15-S1-S6-S4.doc]

Additional file 4 - Significantly expression changed of conserved miRNAs identified in *P. euphratica* between treated root (3dSR) and control root (3dCKR) libraries.

| pairwise | miR-name | 3dCKR-std | 3dSR-std | fold-change(log2 3dSR/3dCKR) | p-value | sig-lable |
| --- | --- | --- | --- | --- | --- | --- |
| 3dCKR-3dSR | miR1310 | 103.8708 | 118.3737 | 0.18855844 | 8.07990282904989e-05 |  |
| 3dCKR-3dSR | miR1444a | 5.0960 | 0.9997 | -2.34979815 | 2.25799174068711e-12 | ** |
| 3dCKR-3dSR | miR1446a | 148.3509 | 175.9434 | 0.24609772 | 5.28401637335666e-10 |  |
| 3dCKR-3dSR | miR1447 | 13.2748 | 19.5819 | 0.56083063 | 8.10372326589448e-06 |  |
| 3dCKR-3dSR | miR1448 | 66.1225 | 110.1410 | 0.73613844 | 1.82718897525085e-41 |  |
| 3dCKR-3dSR | miR1450 | 214.2218 | 265.5616 | 0.30994125 | 2.03625607083356e-21 |  |
| 3dCKR-3dSR | miR1508b | 20.6358 | 36.2236 | 0.81178056 | 3.9761892650806e-17 |  |
| 3dCKR-3dSR | miR1511 | 47.3742 | 52.7477 | 0.15500661 | 0.0296395479189641 |  |
| 3dCKR-3dSR | miR1515 | 1.5099 | 2.3522 | 0.63955773 | 0.0853547527154504 |  |
| 3dCKR-3dSR | miR1520d | 5521.6366 | 5524.9873 | 0.00087521 | 0.897261181897406 |  |
| 3dCKR-3dSR | miR1523a | 22.9636 | 20.5228 | -0.16212125 | 0.133187591999724 |  |
| 3dCKR-3dSR | miR1535a | 44.2285 | 53.5710 | 0.27647591 | 0.000129308292221264 |  |
| 3dCKR-3dSR | miR156a | 19226.2298 | 22001.0398 | 0.19449583 | 0 |  |
| 3dCKR-3dSR | miR156b-3p | 69.8973 | 113.0225 | 0.69330137 | 1.91714391766649e-38 |  |
| 3dCKR-3dSR | miR157a | 42805.2191 | 98921.4117 | 1.20849612 | 0 | ** |
| 3dCKR-3dSR | miR157d-3p | 3.3344 | 5.9981 | 0.84707839 | 0.000406387821837124 |  |
| 3dCKR-3dSR | miR159a | 223.9734 | 475.1413 | 1.08502921 | 0 | ** |
| 3dCKR-3dSR | miR160a | 14.0298 | 59.0986 | 2.07462951 | 7.52457453550316e-108 | ** |
| 3dCKR-3dSR | miR160b-3p | 25.2914 | 28.1674 | 0.15537950 | 0.111427075214292 |  |
| 3dCKR-3dSR | miR162a | 69.3311 | 94.3814 | 0.44499992 | 1.98478814184731e-15 |  |
| 3dCKR-3dSR | miR164a | 2439.9261 | 3946.3187 | 0.69367002 | 0 |  |
| 3dCKR-3dSR | miR165a | 87.7649 | 49.1607 | -0.83613862 | 1.96203057640387e-41 |  |
| 3dCKR-3dSR | miR165a-3p | 51.2748 | 24.2275 | -1.08160442 | 2.10321717779204e-37 | ** |
| 3dCKR-3dSR | miR166a | 17115.2175 | 16321.8080 | -0.06847875 | 2.86851009204057e-69 |  |
| 3dCKR-3dSR | miR166h-3p | 4464.0543 | 6591.4089 | 0.56223230 | 0 |  |
| 3dCKR-3dSR | miR167f-3p | 164.5198 | 101.7320 | -0.69348767 | 1.73641782175165e-55 |  |
| 3dCKR-3dSR | miR167h | 6635.2122 | 7316.6462 | 0.14103989 | 6.29763902190874e-121 |  |
| 3dCKR-3dSR | miR168a | 5985.3119 | 5207.8540 | -0.20073743 | 2.78167782682924e-195 |  |
| 3dCKR-3dSR | miR168a-3p | 51.0232 | 48.5726 | -0.07101067 | 0.319102482561755 |  |
| 3dCKR-3dSR | miR169ac | 388.1786 | 437.9767 | 0.17413354 | 2.18721181419832e-12 |  |
| 3dCKR-3dSR | miR169n-3p | 9.3742 | 8.2326 | -0.18734744 | 0.269605628265353 |  |
| 3dCKR-3dSR | miR171b-3p | 1249.9100 | 1473.6436 | 0.23756343 | 1.18341367630511e-67 |  |
| 3dCKR-3dSR | miR171e | 60.0828 | 74.6819 | 0.31380658 | 3.44531691351425e-07 |  |
| 3dCKR-3dSR | miR172a | 104.0596 | 186.2342 | 0.83970797 | 5.76161491029343e-86 |  |
| 3dCKR-3dSR | miR172a-3p | 102.1092 | 179.5305 | 0.81411610 | 9.28240997061198e-79 |  |
| 3dCKR-3dSR | miR1886.2 | 16.4205 | 23.5218 | 0.51850041 | 5.20364369369561e-06 |  |
| 3dCKR-3dSR | miR2086-3p | 66.6258 | 53.4534 | -0.31779923 | 1.05991462822745e-06 |  |
| 3dCKR-3dSR | miR2089-3p | 26.6126 | 103.9666 | 1.96593876 | 1.13562718834373e-175 | ** |
| 3dCKR-3dSR | miR2111a | 3.0199 | 5.7629 | 0.93229421 | 0.000171726286835148 |  |
| 3dCKR-3dSR | miR2119 | 7.5497 | 10.5848 | 0.48750279 | 0.00391533814198702 |  |
| 3dCKR-3dSR | miR2199 | 4418.5046 | 3007.7971 | -0.55485094 | 0 |  |
| 3dCKR-3dSR | miR2604 | 124.8840 | 156.7731 | 0.32808938 | 1.319016309939e-14 |  |
| 3dCKR-3dSR | miR2630a | 7.8013 | 5.8217 | -0.42227406 | 0.029471722907684 |  |
| 3dCKR-3dSR | miR2637 | 13.5265 | 11.6433 | -0.21628858 | 0.1277662702559 |  |
| 3dCKR-3dSR | miR2651 | 15.7285 | 15.5832 | -0.01338957 | 0.914721303961456 |  |
| 3dCKR-3dSR | miR2666 | 20.8245 | 38.4582 | 0.88500938 | 1.04525072730201e-20 |  |
| 3dCKR-3dSR | miR2911 | 4447.7596 | 3333.0454 | -0.41623784 | 0 |  |
| 3dCKR-3dSR | miR2912a | 675.0030 | 588.6930 | -0.19737844 | 6.88806289059051e-23 |  |
| 3dCKR-3dSR | miR2913 | 7.1722 | 5.5864 | -0.36049684 | 0.0716643203778577 |  |
| 3dCKR-3dSR | miR2916 | 1036.5691 | 1125.4027 | 0.11862504 | 9.68275813433491e-15 |  |
| 3dCKR-3dSR | miR2936 | 77.1324 | 134.5450 | 0.80267987 | 4.25491042142766e-58 |  |
| 3dCKR-3dSR | miR2938 | 71.1556 | 61.3920 | -0.21292663 | 0.000580287282990616 |  |
| 3dCKR-3dSR | miR319b-5p | 1.5728 | 2.6462 | 0.75058688 | 0.0354300170988905 |  |
| 3dCKR-3dSR | miR3434-3p | 21.0761 | 17.2886 | -0.28578688 | 0.0130774378782832 |  |
| 3dCKR-3dSR | miR3509-5p | 67.5066 | 59.5103 | -0.18188917 | 0.00399947656876324 |  |
| 3dCKR-3dSR | miR3514-5p | 0.3775 | 3.5871 | 3.24826942 | 5.25382618243305e-12 | ** |
| 3dCKR-3dSR | miR3626-5p | 5.1589 | 12.0549 | 1.22448430 | 1.04575763394525e-11 | ** |
| 3dCKR-3dSR | miR3627-5p | 5.2848 | 2.7638 | -0.93519565 | 0.00028730210709897 |  |
| 3dCKR-3dSR | miR390a | 40.9569 | 72.9177 | 0.83216253 | 3.17462736036846e-34 |  |
| 3dCKR-3dSR | miR390d-3p | 0.8179 | 1.1173 | 0.45002024 | 0.395455522053773 |  |
| 3dCKR-3dSR | miR391 | 3.9007 | 2.2934 | -0.76624504 | 0.00864168418939913 |  |
| 3dCKR-3dSR | miR393a-3p | 2.8311 | 1.5877 | -0.83442437 | 0.0162430727655239 |  |
| 3dCKR-3dSR | miR393h | 7.5497 | 6.5861 | -0.19699490 | 0.298344430322474 |  |
| 3dCKR-3dSR | miR3949 | 1.8245 | 1.4701 | -0.31158686 | 0.429360605319861 |  |
| 3dCKR-3dSR | miR394a | 0.8808 | 23.0514 | 4.70989609 | 5.65628604925821e-92 | ** |
| 3dCKR-3dSR | miR394b-3p | 94.7483 | 95.6751 | 0.01404345 | 0.786095047838871 |  |
| 3dCKR-3dSR | miR3954 | 189.9999 | 294.1407 | 0.63050776 | 1.8729000144314e-82 |  |
| 3dCKR-3dSR | miR395a | 0.6291 | 1.9406 | 1.62514151 | 0.000872906653499484 | ** |
| 3dCKR-3dSR | miR396a | 372.2614 | 558.2910 | 0.58470127 | 1.69996654909978e-135 |  |
| 3dCKR-3dSR | miR396b-3p | 362.8873 | 443.6220 | 0.28980935 | 9.75994250925969e-31 |  |
| 3dCKR-3dSR | miR397a | 29.0033 | 6.1745 | -2.23182284 | 9.33139444975715e-60 | ** |
| 3dCKR-3dSR | miR398c-5p | 1.9503 | 0.4116 | -2.24438117 | 2.90705858790118e-05 | ** |
| 3dCKR-3dSR | miR399f | 3.3344 | 11.0553 | 1.72923909 | 3.76600133941262e-17 | ** |
| 3dCKR-3dSR | miR403c-5p | 72.2880 | 65.9788 | -0.13175364 | 0.0295319791204412 |  |
| 3dCKR-3dSR | miR408b | 447.1919 | 57.0405 | -2.97083552 | 0 | ** |
| 3dCKR-3dSR | miR415 | 2.3278 | 1.6465 | -0.49956460 | 0.16660766946019 |  |
| 3dCKR-3dSR | miR4343a | 1.0066 | 1.5289 | 0.60300355 | 0.191106883692747 |  |
| 3dCKR-3dSR | miR4348 | 2.7682 | 4.2927 | 0.63293717 | 0.0205593960352556 |  |
| 3dCKR-3dSR | miR4399 | 53.9172 | 131.0167 | 1.28093323 | 7.77499686324955e-120 | ** |
| 3dCKR-3dSR | miR4413a | 586.7348 | 2.0582 | -8.15518156 | 0 | ** |
| 3dCKR-3dSR | miR4414b | 11.7649 | 6.9389 | -0.76171018 | 5.37838478855029e-06 |  |
| 3dCKR-3dSR | miR472b | 361.1257 | 802.2125 | 1.15148335 | 0 | ** |
| 3dCKR-3dSR | miR473a-3p | 25.5430 | 20.8756 | -0.29111031 | 0.00542992462418667 |  |
| 3dCKR-3dSR | miR473a-5p | 92.8609 | 104.4370 | 0.16948975 | 0.000840294631879557 |  |
| 3dCKR-3dSR | miR475a-3p | 53.5397 | 54.2179 | 0.01816018 | 0.792054317876225 |  |
| 3dCKR-3dSR | miR475a-5p | 82.9205 | 100.6735 | 0.27988326 | 1.09355722812892e-07 |  |
| 3dCKR-3dSR | miR477a-3p | 28.8146 | 21.8165 | -0.40138032 | 6.47274923202659e-05 |  |
| 3dCKR-3dSR | miR477a-5p | 153.8244 | 165.0057 | 0.10123150 | 0.0111745088418669 |  |
| 3dCKR-3dSR | miR479 | 36.1755 | 49.5723 | 0.45452124 | 4.41994332356419e-09 |  |
| 3dCKR-3dSR | miR482a | 67.6953 | 111.1995 | 0.71602272 | 5.94120894589789e-40 |  |
| 3dCKR-3dSR | miR482c-3p | 33.1556 | 48.3962 | 0.54564121 | 7.39947309243456e-12 |  |
| 3dCKR-3dSR | miR4993 | 2.1391 | 4.5868 | 1.10048407 | 0.000124703664430536 | ** |
| 3dCKR-3dSR | miR4995 | 14.4702 | 8.9383 | -0.69501249 | 3.19116757509239e-06 |  |
| 3dCKR-3dSR | miR5020b | 46.9338 | 55.6292 | 0.24521509 | 0.000504142297701924 |  |
| 3dCKR-3dSR | miR5021 | 10.5066 | 15.0540 | 0.51885100 | 0.000267698810422962 |  |
| 3dCKR-3dSR | miR5037c | 75.9371 | 88.1481 | 0.21512457 | 0.000112173635150567 |  |
| 3dCKR-3dSR | miR5139 | 24.6622 | 17.4650 | -0.49783486 | 6.53721481838906e-06 |  |
| 3dCKR-3dSR | miR5140 | 17.1755 | 38.5170 | 1.16514324 | 1.17283990233771e-31 | ** |
| 3dCKR-3dSR | miR5210 | 30.6391 | 24.8155 | -0.30413240 | 0.00150242719622958 |  |
| 3dCKR-3dSR | miR5218 | 56.6854 | 65.8024 | 0.21516300 | 0.000845792049071386 |  |
| 3dCKR-3dSR | miR5221 | 374.7151 | 29.5787 | -3.66316356 | 0 | ** |
| 3dCKR-3dSR | miR5224b | 146.5893 | 371.9980 | 1.34351506 | 0 | ** |
| 3dCKR-3dSR | miR5229a | 2.4536 | 1.4113 | -0.79787538 | 0.031446701573842 |  |
| 3dCKR-3dSR | miR5230 | 539.0461 | 465.2033 | -0.21254733 | 3.27117092427407e-21 |  |
| 3dCKR-3dSR | miR5234 | 2.7682 | 7.9386 | 1.51993643 | 8.22157383087562e-11 | ** |
| 3dCKR-3dSR | miR5239 | 1.1324 | 1.8229 | 0.68685177 | 0.107958966632928 |  |
| 3dCKR-3dSR | miR5248 | 1.1954 | 0.9997 | -0.25792632 | 0.592720383243961 |  |
| 3dCKR-3dSR | miR5255 | 12.3940 | 8.5267 | -0.53958247 | 0.000587286361942232 |  |
| 3dCKR-3dSR | miR5260 | 16.1060 | 25.8740 | 0.68390487 | 9.11150467939108e-10 |  |
| 3dCKR-3dSR | miR5263 | 1.6987 | 0.5880 | -1.53054303 | 0.00263867789503333 | ** |
| 3dCKR-3dSR | miR5265 | 4361.8192 | 4915.9479 | 0.17253966 | 2.86176136098157e-120 |  |
| 3dCKR-3dSR | miR529 | 9.3742 | 10.8200 | 0.20693302 | 0.193769368467028 |  |
| 3dCKR-3dSR | miR5296 | 1.6987 | 2.1170 | 0.31759018 | 0.392719342829964 |  |
| 3dCKR-3dSR | miR5298b | 1.6987 | 1.4701 | -0.20851679 | 0.602144458403605 |  |
| 3dCKR-3dSR | miR5301 | 85.8774 | 72.9765 | -0.23484655 | 3.27740195461357e-05 |  |
| 3dCKR-3dSR | miR530b | 0.6921 | 2.8814 | 2.05771754 | 1.68448104951652e-06 | ** |
| 3dCKR-3dSR | miR535a | 2.8940 | 3.4107 | 0.23700294 | 0.409349712450731 |  |
| 3dCKR-3dSR | miR5368 | 36.8675 | 27.9910 | -0.39738656 | 7.5617189437297e-06 |  |
| 3dCKR-3dSR | miR5373 | 7.9901 | 7.2918 | -0.13193857 | 0.468115772809232 |  |
| 3dCKR-3dSR | miR5561-5p | 1.5728 | 2.1170 | 0.42868604 | 0.25737231016915 |  |
| 3dCKR-3dSR | miR5562-3p | 3.3973 | 24.2863 | 2.83768219 | 1.24908248868767e-64 | ** |
| 3dCKR-3dSR | miR5632 | 5.5993 | 2.6462 | -1.08132437 | 2.56210361081806e-05 | ** |
| 3dCKR-3dSR | miR5646 | 361.2515 | 414.7489 | 0.19923458 | 7.02911037275553e-15 |  |
| 3dCKR-3dSR | miR5647 | 11.1987 | 19.0527 | 0.76666419 | 6.46854820020491e-09 |  |
| 3dCKR-3dSR | miR5652 | 1.5099 | 2.2934 | 0.60303500 | 0.107084917911852 |  |
| 3dCKR-3dSR | miR5656 | 3.8377 | 4.2339 | 0.14174526 | 0.576260786528574 |  |
| 3dCKR-3dSR | miR5657 | 0.7550 | 7.4682 | 3.30621201 | 5.38602104032598e-24 | ** |
| 3dCKR-3dSR | miR5658 | 102.3609 | 179.2953 | 0.80867293 | 8.85422444043814e-78 |  |
| 3dCKR-3dSR | miR5665 | 6.7947 | 6.9977 | 0.04247096 | 0.827383825463351 |  |
| 3dCKR-3dSR | miR5671 | 657.1984 | 438.3884 | -0.58411934 | 1.39138547965343e-159 |  |
| 3dCKR-3dSR | miR5672 | 1.2583 | 2.9402 | 1.22443837 | 0.000862832640013301 | ** |
| 3dCKR-3dSR | miR5740 | 0.1887 | 1.1761 | 2.63984441 | 0.000507081084911916 | ** |
| 3dCKR-3dSR | miR5755 | 7.8013 | 7.0566 | -0.14474132 | 0.432684663284143 |  |
| 3dCKR-3dSR | miR5759 | 0.7550 | 1.0585 | 0.48747272 | 0.373936551681004 |  |
| 3dCKR-3dSR | miR5772 | 48.0662 | 68.3310 | 0.50751749 | 2.48859859868477e-14 |  |
| 3dCKR-3dSR | miR5776 | 1.0695 | 0.9409 | -0.18482318 | 0.711799875475164 |  |
| 3dCKR-3dSR | miR6022 | 51.0861 | 47.8081 | -0.09567573 | 0.181176351155227 |  |
| 3dCKR-3dSR | miR6028 | 2.5166 | 0.6469 | -1.95986131 | 1.23865446313988e-05 | ** |
| 3dCKR-3dSR | miR6032 | 2.7053 | 10.1144 | 1.90255025 | 8.24172301179713e-18 | ** |
| 3dCKR-3dSR | miR6035 | 10.3179 | 1.2349 | -3.06268324 | 4.44145470265674e-31 | ** |
| 3dCKR-3dSR | miR6103-3p | 13.0861 | 10.6436 | -0.29804900 | 0.0418863371652934 |  |
| 3dCKR-3dSR | miR6145e | 1.8245 | 3.7635 | 1.04457382 | 0.000879339336873964 | ** |
| 3dCKR-3dSR | miR6171 | 27.9967 | 21.9341 | -0.35208127 | 0.00049471923725572 |  |
| 3dCKR-3dSR | miR6173 | 38.1887 | 26.9913 | -0.50065135 | 1.70230900835778e-08 |  |
| 3dCKR-3dSR | miR6300 | 1387.0623 | 941.2266 | -0.55941859 | 1.47677868728665e-309 |  |
| 3dCKR-3dSR | miR6421-3p | 512.1190 | 657.5532 | 0.36062854 | 1.18315367607175e-66 |  |
| 3dCKR-3dSR | miR6425a-5p | 8.1159 | 5.5864 | -0.53883221 | 0.00549430267144629 |  |
| 3dCKR-3dSR | miR6426a | 35.8609 | 42.2217 | 0.23557297 | 0.00355003942868745 |  |
| 3dCKR-3dSR | miR6428 | 113.6225 | 135.0154 | 0.24887542 | 3.82497913416314e-08 |  |
| 3dCKR-3dSR | miR6433-3p | 5.6622 | 6.3509 | 0.16559834 | 0.423837350552309 |  |
| 3dCKR-3dSR | miR6433-5p | 3.9007 | 5.0572 | 0.37460579 | 0.119350681137573 |  |
| 3dCKR-3dSR | miR6438b | 2.3278 | 1.4701 | -0.66305282 | 0.0746086531915226 |  |
| 3dCKR-3dSR | miR6439a | 1.3841 | 1.6465 | 0.25045433 | 0.550019370181036 |  |
| 3dCKR-3dSR | miR6441 | 1863.8866 | 2443.5668 | 0.39067446 | 3.85212896760071e-281 |  |
| 3dCKR-3dSR | miR6445a | 14.2185 | 28.2850 | 0.99226790 | 1.32954818218441e-18 |  |
| 3dCKR-3dSR | miR6448 | 6.7947 | 16.1125 | 1.24569860 | 1.54378653001403e-15 | ** |
| 3dCKR-3dSR | miR6450a | 293.3045 | 206.1690 | -0.50857178 | 1.20154745225861e-56 |  |
| 3dCKR-3dSR | miR6453 | 53.0993 | 14.9952 | -1.82419208 | 3.23746115756789e-83 | ** |
| 3dCKR-3dSR | miR6454 | 37.5596 | 94.3226 | 1.32842179 | 1.03148701245362e-91 | ** |
| 3dCKR-3dSR | miR6457b | 1.0695 | 0.8821 | -0.27792236 | 0.58727590880111 |  |
| 3dCKR-3dSR | miR6460 | 3.5232 | 1.2937 | -1.44538327 | 2.90257649063995e-05 | ** |
| 3dCKR-3dSR | miR6462c-5p | 6.6060 | 3.9987 | -0.72424592 | 0.00111802668285044 |  |
| 3dCKR-3dSR | miR6466-5p | 2.0132 | 2.0582 | 0.03189268 | 0.933067724431713 |  |
| 3dCKR-3dSR | miR6471 | 14.9735 | 11.0553 | -0.43767331 | 0.00181075029026826 |  |
| 3dCKR-3dSR | miR6474 | 23.3410 | 52.4537 | 1.16817817 | 1.11743123965604e-42 | ** |
| 3dCKR-3dSR | miR6476 | 20.1324 | 27.0501 | 0.42611476 | 4.47266795345105e-05 |  |
| 3dCKR-3dSR | miR6478 | 224.6655 | 158.4196 | -0.50402776 | 4.0197067445649e-43 |  |
| 3dCKR-3dSR | miR6485 | 9.5629 | 9.8792 | 0.04694603 | 0.773589068007075 |  |
| 3dCKR-3dSR | miR774b-5p | 31.1424 | 29.3435 | -0.08583917 | 0.347892156206625 |  |
| 3dCKR-3dSR | miR780.2 | 6.8576 | 4.5868 | -0.58021575 | 0.0063744273998813 |  |
| 3dCKR-3dSR | miR827 | 5.5364 | 12.1137 | 1.12961950 | 1.61833919582934e-10 | ** |
| 3dCKR-3dSR | miR837-3p | 4.7185 | 3.6459 | -0.37205331 | 0.132576945593534 |  |
| 3dCKR-3dSR | miR845b-5p | 204.5959 | 150.1870 | -0.44601729 | 7.77287785517446e-32 |  |
| 3dCKR-3dSR | miR846-5p | 8.3046 | 16.0537 | 0.95092326 | 1.5878250669329e-10 |  |
| 3dCKR-3dSR | miR847-5p | 3.9636 | 3.7635 | -0.07473640 | 0.768398374350129 |  |
| 3dCKR-3dSR | miR858 | 22.1457 | 27.9910 | 0.33793643 | 0.000825862346000891 |  |
| 3dCKR-3dSR | miR860 | 8.3675 | 6.2333 | -0.42480050 | 0.0233658116348466 |  |
| 3dCKR-3dSR | miR860-3p | 25.9205 | 98.7329 | 1.92943736 | 7.20029427944957e-163 | ** |
| 3dCKR-3dSR | miR948 | 0.3146 | 1.2349 | 1.97280364 | 0.00269197515341632 | ** |
