# Supplementary material for: Genome-wide analysis of salt-responsive and novel microRNAs in Populus euphratica by deep sequencing
Source: BMC Genet. 2014 Jun 20;15(Suppl 1):S6. doi: 10.1186/1471-2156-15-S1-S6 (PMC4118626; doi:10.1186/1471-2156-15-S1-S6)
Supplement: Additional file 5 — Summary of target genes of novel miRNAs from the root tissue. [file 1471-2156-15-S1-S6-S5.doc]

Additional file 5 - Summary of target genes of novel miRNAs from root tissue.

| MiRNA name | Targets number | Targets ID |
| --- | --- | --- |
| 3dSR-m0001_5p | 15 | Potri.017G041800.1[]~~Potri.017G041800.2[]~~Potri.017G041800.5[]~~Potri.017G041800.3[]~~Potri.017G041800.6[]~~Potri.017G041800.4[]~~Potri.018G139800.2[]~~Potri.018G139800.5[]~~Potri.018G139800.6[]~~Potri.018G139800.1[]~~Potri.018G139800.3[]~~Potri.015G008200.1[]~~Potri.001G025400.1[]~~Potri.001G029000.1[]~~Potri.001G029000.2[] |
| 3dSR-m0004_5p | 3 | Potri.005G007000.1[]~~Potri.001G379700.1[]~~Potri.001G379700.2[] |
| 3dSR-m0005_3p | 19 | Potri.003G190700.1[]~~Potri.011G104700.1[]~~Potri.011G104900.1[]~~Potri.011G105100.1[]~~Potri.002G048300.2[]~~Potri.002G048300.5[]~~Potri.002G048300.3[]~~Potri.002G048300.4[]~~Potri.002G048300.1[]~~Potri.T085700.2[]~~Potri.T085700.3[]~~Potri.T085700.1[]~~Potri.T086100.3[]~~Potri.T086100.1[]~~Potri.T086100.2[]~~Potri.001G029400.1[]~~Potri.001G034700.1[]~~Potri.001G034900.1[]~~Potri.001G035300.1[] |
| 3dSR-m0006_5p | 1 | Potri.001G455100.1[] |
| 3dSR-m0007_3p | 1 | Potri.001G354200.1[] |
| 3dSR-m0008_5p | 3 | Potri.003G175400.1[]~~Potri.001G298400.2[]~~Potri.001G298400.1[] |
| 3dSR-m0009_5p | 3 | Potri.003G175400.1[]~~Potri.001G298400.2[]~~Potri.001G298400.1[] |
| 3dSR-m0010_5p | 3 | Potri.003G175400.1[]~~Potri.001G298400.2[]~~Potri.001G298400.1[] |
| 3dSR-m0011_5p | 3 | Potri.003G175400.1[]~~Potri.001G298400.2[]~~Potri.001G298400.1[] |
| 3dSR-m0012_5p | 3 | Potri.003G175400.1[]~~Potri.001G298400.2[]~~Potri.001G298400.1[] |
| 3dSR-m0013_5p | 23 | Potri.004G164500.2[]~~Potri.004G164500.3[]~~Potri.004G164500.1[]~~Potri.003G034200.1[]~~Potri.003G175700.1[]~~Potri.011G122100.1[]~~Potri.011G122100.2[]~~Potri.011G122100.3[]~~Potri.011G155900.1[]~~Potri.018G021400.1[]~~Potri.018G021400.2[]~~Potri.016G054700.6[]~~Potri.016G122400.1[]~~Potri.016G141500.2[]~~Potri.002G130400.1[]~~Potri.014G036000.1[]~~Potri.001G052500.1[]~~Potri.001G459100.1[]~~Potri.012G035900.1[]~~Potri.012G035900.3[]~~Potri.012G035900.4[]~~Potri.012G035900.2[]~~Potri.012G036100.1[] |
| 3dSR-m0014_3p | 2 | Potri.009G067800.1[]~~Potri.009G067800.2[] |
| 3dSR-m0017_5p | 1 | Potri.001G455100.1[] |
| 3dSR-m0018_5p | 2 | Potri.006G170700.2[]~~Potri.006G170700.1[] |
| 3dSR-m0023_3p | 8 | Potri.003G198900.1[]~~Potri.003G199100.1[]~~Potri.003G199400.2[]~~Potri.003G199400.1[]~~Potri.003G199500.1[]~~Potri.003G199600.1[]~~Potri.003G200200.1[]~~Potri.003G200500.1[] |
| 3dSR-m0024_3p | 30 | Potri.004G005500.3[]~~Potri.004G005500.6[]~~Potri.004G005500.5[]~~Potri.004G005500.2[]~~Potri.004G005500.1[]~~Potri.004G005500.4[]~~Potri.003G152000.1[]~~Potri.003G152300.1[]~~Potri.003G152300.2[]~~Potri.003G152400.1[]~~Potri.003G152400.3[]~~Potri.003G152400.2[]~~Potri.003G152400.4[]~~Potri.006G148400.1[]~~Potri.006G168600.1[]~~Potri.005G033400.1[]~~Potri.001G077600.2[]~~Potri.001G077600.3[]~~Potri.001G077600.1[]~~Potri.001G077600.4[]~~Potri.001G078100.1[]~~Potri.001G422300.1[]~~Potri.001G471200.2[]~~Potri.001G471200.3[]~~Potri.001G471200.4[]~~Potri.001G471200.5[]~~Potri.001G471200.6[]~~Potri.001G471200.7[]~~Potri.001G471200.1[]~~Potri.001G471200.8[] |
| 3dSR-m0025_3p | 9 | Potri.T171800.1[]~~Potri.009G147300.1[]~~Potri.011G044400.1[]~~Potri.018G026500.2[]~~Potri.018G026500.1[]~~Potri.018G026500.3[]~~Potri.014G138300.1[]~~Potri.014G138300.1[]~~Potri.008G008900.1[] |
| 3dSR-m0026_3p | 5 | Potri.011G052600.1[]~~Potri.011G052600.1[]~~Potri.011G052600.1[]~~Potri.011G052600.1[]~~Potri.011G052600.1[] |
| 3dSR-m0030_5p | 5 | Potri.010G140300.1[]~~Potri.010G212000.1[]~~Potri.001G289800.3[]~~Potri.001G289800.2[]~~Potri.001G289800.1[] |
| 3dSR-m0032_3p | 1 | Potri.013G035400.1[] |
| 3dSR-m0034_3p | 30 | Potri.004G005500.3[]~~Potri.004G005500.6[]~~Potri.004G005500.5[]~~Potri.004G005500.2[]~~Potri.004G005500.1[]~~Potri.004G005500.4[]~~Potri.003G152000.1[]~~Potri.003G152300.1[]~~Potri.003G152300.2[]~~Potri.003G152400.1[]~~Potri.003G152400.3[]~~Potri.003G152400.2[]~~Potri.003G152400.4[]~~Potri.006G148400.1[]~~Potri.006G168600.1[]~~Potri.005G033400.1[]~~Potri.001G077600.2[]~~Potri.001G077600.3[]~~Potri.001G077600.1[]~~Potri.001G077600.4[]~~Potri.001G078100.1[]~~Potri.001G422300.1[]~~Potri.001G471200.2[]~~Potri.001G471200.3[]~~Potri.001G471200.4[]~~Potri.001G471200.5[]~~Potri.001G471200.6[]~~Potri.001G471200.7[]~~Potri.001G471200.1[]~~Potri.001G471200.8[] |
| 3dSR-m0035_3p | 12 | Potri.003G152000.1[]~~Potri.003G152300.1[]~~Potri.003G152300.2[]~~Potri.015G135100.1[]~~Potri.001G077600.2[]~~Potri.001G077600.3[]~~Potri.001G077600.1[]~~Potri.001G077600.4[]~~Potri.001G078100.1[]~~Potri.001G422000.1[]~~Potri.001G422000.2[]~~Potri.001G422300.1[] |
| 3dSR-m0036_3p | 30 | Potri.004G005500.3[]~~Potri.004G005500.6[]~~Potri.004G005500.5[]~~Potri.004G005500.2[]~~Potri.004G005500.1[]~~Potri.004G005500.4[]~~Potri.003G152000.1[]~~Potri.003G152300.1[]~~Potri.003G152300.2[]~~Potri.003G152400.1[]~~Potri.003G152400.3[]~~Potri.003G152400.2[]~~Potri.003G152400.4[]~~Potri.006G148400.1[]~~Potri.006G168600.1[]~~Potri.005G033400.1[]~~Potri.001G077600.2[]~~Potri.001G077600.3[]~~Potri.001G077600.1[]~~Potri.001G077600.4[]~~Potri.001G078100.1[]~~Potri.001G422300.1[]~~Potri.001G471200.2[]~~Potri.001G471200.3[]~~Potri.001G471200.4[]~~Potri.001G471200.5[]~~Potri.001G471200.6[]~~Potri.001G471200.7[]~~Potri.001G471200.1[]~~Potri.001G471200.8[] |
| 3dSR-m0037_3p | 12 | Potri.003G152000.1[]~~Potri.003G152300.1[]~~Potri.003G152300.2[]~~Potri.015G135100.1[]~~Potri.001G077600.2[]~~Potri.001G077600.3[]~~Potri.001G077600.1[]~~Potri.001G077600.4[]~~Potri.001G078100.1[]~~Potri.001G422000.1[]~~Potri.001G422000.2[]~~Potri.001G422300.1[] |
| 3dSR-m0038_3p | 30 | Potri.004G005500.3[]~~Potri.004G005500.6[]~~Potri.004G005500.5[]~~Potri.004G005500.2[]~~Potri.004G005500.1[]~~Potri.004G005500.4[]~~Potri.003G152000.1[]~~Potri.003G152300.1[]~~Potri.003G152300.2[]~~Potri.003G152400.1[]~~Potri.003G152400.3[]~~Potri.003G152400.2[]~~Potri.003G152400.4[]~~Potri.006G148400.1[]~~Potri.006G168600.1[]~~Potri.005G033400.1[]~~Potri.001G077600.2[]~~Potri.001G077600.3[]~~Potri.001G077600.1[]~~Potri.001G077600.4[]~~Potri.001G078100.1[]~~Potri.001G422300.1[]~~Potri.001G471200.2[]~~Potri.001G471200.3[]~~Potri.001G471200.4[]~~Potri.001G471200.5[]~~Potri.001G471200.6[]~~Potri.001G471200.7[]~~Potri.001G471200.1[]~~Potri.001G471200.8[] |
| 3dSR-m0039_5p | 1 | Potri.006G007300.1[] |
| 3dSR-m0041_3p | 1 | Potri.004G011700.1[] |
| 3dSR-m0043_3p | 36 | Potri.010G252200.1[]~~Potri.017G030100.1[]~~Potri.009G075700.1[]~~Potri.004G035100.1[]~~Potri.004G035100.2[]~~Potri.003G167400.1[]~~Potri.011G143300.1[]~~Potri.011G143300.2[]~~Potri.006G040500.1[]~~Potri.006G114900.1[]~~Potri.018G023400.1[]~~Potri.018G023400.2[]~~Potri.018G023400.3[]~~Potri.018G023400.4[]~~Potri.007G016300.2[]~~Potri.007G016300.1[]~~Potri.007G054400.1[]~~Potri.002G011400.1[]~~Potri.002G028200.1[]~~Potri.002G089100.2[]~~Potri.002G127100.1[]~~Potri.002G127100.2[]~~Potri.019G053700.1[]~~Potri.019G053700.1[]~~Potri.019G057200.1[]~~Potri.005G060900.1[]~~Potri.005G066300.2[]~~Potri.005G066300.3[]~~Potri.005G066300.1[]~~Potri.005G122700.1[]~~Potri.005G234500.1[]~~Potri.005G234500.2[]~~Potri.008G027400.2[]~~Potri.001G271900.1[]~~Potri.001G348300.1[]~~Potri.001G348300.2[] |
| 3dSR-m0044_3p | 8 | Potri.003G151900.1[]~~Potri.003G152100.1[]~~Potri.003G152400.1[]~~Potri.003G152400.3[]~~Potri.003G152400.2[]~~Potri.003G152400.4[]~~Potri.005G063300.2[]~~Potri.005G063300.3[] |
| 3dSR-m0045_3p | 6 | Potri.009G014500.1[]~~Potri.009G014500.2[]~~Potri.009G014500.3[]~~Potri.009G014500.4[]~~Potri.002G174100.2[]~~Potri.002G174100.1[] |
| 3dSR-m0048_5p | 1 | Potri.001G168400.1[] |
| 3dSR-m0049_3p | 8 | Potri.010G047400.1[]~~Potri.009G024000.1[]~~Potri.019G104600.2[]~~Potri.019G104600.1[]~~Potri.005G091500.1[]~~Potri.001G325400.2[]~~Potri.001G325400.1[]~~Potri.001G455100.1[] |
| 3dSR-m0052_5p | 1 | Potri.016G026300.1[] |
| 3dSR-m0053_3p | 1 | Potri.003G188400.1[] |
| 3dSR-m0056_5p | 1 | Potri.001G455100.1[] |
| 3dSR-m0057_3p | 6 | Potri.003G131200.2[]~~Potri.003G131200.3[]~~Potri.003G131200.4[]~~Potri.003G131200.1[]~~Potri.005G094000.2[]~~Potri.005G094000.1[] |
| 3dSR-m0058_5p | 4 | Potri.017G126900.1[]~~Potri.004G048600.2[]~~Potri.004G048600.3[]~~Potri.004G048600.1[] |
| 3dSR-m0059_5p | 1 | Potri.008G016900.1[] |
| 3dSR-m0060_5p | 3 | Potri.006G170700.2[]~~Potri.006G170700.1[]~~Potri.016G112400.1[] |
| 3dSR-m0065_3p | 2 | Potri.011G108200.1[]~~Potri.011G108300.1[] |
| 3dSR-m0066_5p | 12 | Potri.009G072900.3[]~~Potri.009G072900.2[]~~Potri.009G072900.1[]~~Potri.013G080100.1[]~~Potri.015G105400.1[]~~Potri.014G006800.1[]~~Potri.001G144000.2[]~~Potri.001G144000.1[]~~Potri.001G144000.4[]~~Potri.001G144000.3[]~~Potri.001G144000.5[]~~Potri.001G278400.1[] |
| 3dSR-m0067_5p | 3 | Potri.003G175400.1[]~~Potri.001G298400.2[]~~Potri.001G298400.1[] |
| 3dSR-m0069_3p | 4 | Potri.006G039300.1[]~~Potri.015G094700.2[]~~Potri.015G094700.3[]~~Potri.015G094700.1[] |
| 3dSR-m0070_5p | 12 | Potri.010G177400.3[]~~Potri.010G177400.2[]~~Potri.010G177400.1[]~~Potri.011G108000.2[]~~Potri.011G108000.3[]~~Potri.011G108000.1[]~~Potri.002G241100.1[]~~Potri.001G122100.1[]~~Potri.012G022700.3[]~~Potri.012G022700.4[]~~Potri.012G022700.2[]~~Potri.012G022700.1[] |
| 3dSR-m0072_5p | 4 | Potri.006G050200.5[]~~Potri.006G050200.1[]~~Potri.006G050200.6[]~~Potri.006G050200.2[] |
| 3dSR-m0073_3p | 2 | Potri.009G067800.1[]~~Potri.009G067800.2[] |
| 3dSR-m0074_5p | 1 | Potri.015G066900.1[] |
| 3dSR-m0076_5p | 8 | Potri.009G009100.1[]~~Potri.007G048700.1[]~~Potri.007G048700.2[]~~Potri.002G241100.1[]~~Potri.005G098500.1[]~~Potri.005G098500.3[]~~Potri.005G098500.2[]~~Potri.001G242700.1[] |
| 3dSR-m0082_3p | 7 | Potri.010G121500.1[]~~Potri.010G121500.2[]~~Potri.T038300.1[]~~Potri.T039800.1[]~~Potri.001G128400.1[]~~Potri.001G338000.2[]~~Potri.001G338000.1[] |
| 3dSR-m0083_5p | 1 | Potri.005G211100.1[] |
| 3dSR-m0085_5p | 10 | Potri.013G120800.1[]~~Potri.013G120900.1[]~~Potri.013G121300.1[]~~Potri.013G121400.1[]~~Potri.013G121800.1[]~~Potri.013G121900.1[]~~Potri.013G122000.1[]~~Potri.013G122400.1[]~~Potri.013G122500.1[]~~Potri.013G122900.1[] |
| 3dSR-m0086_3p | 4 | Potri.010G003700.1[]~~Potri.010G003700.3[]~~Potri.010G003700.2[]~~Potri.011G074200.1[] |
| 3dSR-m0089_5p | 3 | Potri.018G034700.1[]~~Potri.018G034700.3[]~~Potri.018G034700.2[] |
| 3dSR-m0090_5p | 1 | Potri.001G455100.1[] |
| 3dSR-m0091_5p | 1 | Potri.001G455100.1[] |
| 3dSR-m0092_3p | 6 | Potri.010G234700.3[]~~Potri.006G017500.1[]~~Potri.019G057500.4[]~~Potri.005G092800.3[]~~Potri.005G092800.2[]~~Potri.005G092800.1[] |
| 3dSR-m0093_3p | 20 | Potri.010G189100.1[]~~Potri.004G197900.1[]~~Potri.004G198600.1[]~~Potri.013G035900.1[]~~Potri.016G054200.3[]~~Potri.016G054200.1[]~~Potri.016G054200.2[]~~Potri.002G012800.1[]~~Potri.005G248500.2[]~~Potri.005G248500.3[]~~Potri.005G248500.1[]~~Potri.014G082100.1[]~~Potri.014G164700.1[]~~Potri.014G164700.2[]~~Potri.008G115700.1[]~~Potri.001G147700.1[]~~Potri.001G166800.4[]~~Potri.001G166800.2[]~~Potri.001G166800.1[]~~Potri.001G166800.3[] |
| 3dSR-m0096_3p | 5 | Potri.003G197500.1[]~~Potri.003G197500.2[]~~Potri.002G235400.1[]~~Potri.014G148900.1[]~~Potri.014G148900.2[] |
| 3dSR-m0098_3p | 10 | Potri.017G115400.1[]~~Potri.017G115400.2[]~~Potri.004G099200.1[]~~Potri.004G099200.3[]~~Potri.004G099200.2[]~~Potri.006G087900.1[]~~Potri.006G087900.2[]~~Potri.016G100400.1[]~~Potri.016G100400.2[]~~Potri.016G100400.3[] |
| 3dSR-m0099_5p | 2 | Potri.004G013800.1[]~~Potri.004G164000.1[] |
| 3dSR-m0100_5p | 3 | Potri.015G091200.1[]~~Potri.008G138700.1[]~~Potri.012G093900.1[] |
| 3dSR-m0101_5p | 4 | Potri.006G106300.1[]~~Potri.002G068900.1[]~~Potri.019G035600.2[]~~Potri.019G035600.1[] |
| 3dSR-m0106_5p | 1 | Potri.006G241800.2[] |
| 3dSR-m0107_5p | 12 | Potri.009G072900.3[]~~Potri.009G072900.2[]~~Potri.009G072900.1[]~~Potri.013G080100.1[]~~Potri.015G105400.1[]~~Potri.014G006800.1[]~~Potri.001G144000.2[]~~Potri.001G144000.1[]~~Potri.001G144000.4[]~~Potri.001G144000.3[]~~Potri.001G144000.5[]~~Potri.001G278400.1[] |
| 3dSR-m0110_5p | 11 | Potri.010G063100.1[]~~Potri.004G065900.1[]~~Potri.004G065900.3[]~~Potri.004G065900.2[]~~Potri.003G144900.2[]~~Potri.003G144900.1[]~~Potri.002G065500.1[]~~Potri.002G137700.2[]~~Potri.002G137700.3[]~~Potri.002G137700.1[]~~Potri.008G116000.1[] |
| 3dSR-m0111_5p | 34 | Potri.009G081600.1[]~~Potri.009G081600.2[]~~Potri.009G081600.3[]~~Potri.009G081600.4[]~~Potri.009G081600.5[]~~Potri.009G081600.6[]~~Potri.009G081600.7[]~~Potri.009G081600.8[]~~Potri.013G025800.1[]~~Potri.011G129400.2[]~~Potri.011G129400.1[]~~Potri.006G027300.3[]~~Potri.006G027300.6[]~~Potri.006G027300.4[]~~Potri.006G027300.2[]~~Potri.006G027300.5[]~~Potri.006G027300.1[]~~Potri.006G137500.1[]~~Potri.006G137500.2[]~~Potri.006G137500.3[]~~Potri.006G137500.4[]~~Potri.006G137500.5[]~~Potri.006G137500.6[]~~Potri.006G137500.7[]~~Potri.018G063300.2[]~~Potri.018G063300.1[]~~Potri.018G063300.3[]~~Potri.018G127000.1[]~~Potri.002G083100.1[]~~Potri.001G287400.2[]~~Potri.001G287400.1[]~~Potri.001G287400.4[]~~Potri.001G287400.3[]~~Potri.012G102000.1[] |
| 3dSR-m0112_5p | 9 | Potri.006G201900.4[]~~Potri.006G201900.3[]~~Potri.006G201900.1[]~~Potri.006G201900.2[]~~Potri.006G201900.5[]~~Potri.016G068200.3[]~~Potri.016G068200.1[]~~Potri.016G068200.2[]~~Potri.015G061600.1[] |
| 3dSR-m0113_5p | 29 | Potri.010G091700.1[]~~Potri.009G052900.2[]~~Potri.009G052900.5[]~~Potri.009G052900.7[]~~Potri.009G052900.3[]~~Potri.009G052900.4[]~~Potri.009G052900.8[]~~Potri.009G052900.6[]~~Potri.009G052900.1[]~~Potri.009G060600.2[]~~Potri.009G060600.4[]~~Potri.009G060600.5[]~~Potri.009G060600.6[]~~Potri.009G060600.3[]~~Potri.009G060600.1[]~~Potri.003G139600.2[]~~Potri.003G139600.3[]~~Potri.003G139600.1[]~~Potri.006G053500.3[]~~Potri.006G053500.1[]~~Potri.006G053500.4[]~~Potri.006G053500.2[]~~Potri.001G257600.1[]~~Potri.001G257600.2[]~~Potri.001G266000.2[]~~Potri.001G266000.5[]~~Potri.001G266000.4[]~~Potri.001G266000.3[]~~Potri.001G266000.1[] |
| 3dSR-m0114_3p | 2 | Potri.001G403500.1[]~~Potri.001G403500.2[] |
| 3dSR-m0115_3p | 1 | Potri.006G081600.1[] |
| 3dSR-m0117_3p | 3 | Potri.013G051600.1[]~~Potri.013G051700.1[]~~Potri.018G005200.1[] |
| 3dSR-m0118_5p | 1 | Potri.005G211100.1[] |
| 3dSR-m0119_3p | 2 | Potri.009G119000.1[]~~Potri.001G191800.1[] |
| 3dSR-m0120_5p | 29 | Potri.010G091700.1[]~~Potri.009G052900.2[]~~Potri.009G052900.5[]~~Potri.009G052900.7[]~~Potri.009G052900.3[]~~Potri.009G052900.4[]~~Potri.009G052900.8[]~~Potri.009G052900.6[]~~Potri.009G052900.1[]~~Potri.009G060600.2[]~~Potri.009G060600.4[]~~Potri.009G060600.5[]~~Potri.009G060600.6[]~~Potri.009G060600.3[]~~Potri.009G060600.1[]~~Potri.003G139600.2[]~~Potri.003G139600.3[]~~Potri.003G139600.1[]~~Potri.006G053500.3[]~~Potri.006G053500.1[]~~Potri.006G053500.4[]~~Potri.006G053500.2[]~~Potri.001G257600.1[]~~Potri.001G257600.2[]~~Potri.001G266000.2[]~~Potri.001G266000.5[]~~Potri.001G266000.4[]~~Potri.001G266000.3[]~~Potri.001G266000.1[] |
| 3dSR-m0121_3p | 1 | Potri.013G035400.1[] |
| 3dSR-m0122_3p | 1 | Potri.019G087600.1[] |
| 3dSR-m0124_3p | 20 | Potri.T001400.1[]~~Potri.T001500.1[]~~Potri.T001700.1[]~~Potri.T001900.1[]~~Potri.T002200.1[]~~Potri.T002300.2[]~~Potri.T002300.1[]~~Potri.T002400.1[]~~Potri.T002500.1[]~~Potri.T002600.1[]~~Potri.T002900.3[]~~Potri.T002900.2[]~~Potri.T002900.1[]~~Potri.T003000.1[]~~Potri.003G014200.2[]~~Potri.003G014200.1[]~~Potri.019G046000.1[]~~Potri.001G066500.1[]~~Potri.T112700.1[]~~Potri.T011800.1[] |
| 3dSR-m0126_3p | 43 | Potri.T044800.1[]~~Potri.T024700.1[]~~Potri.T024900.1[]~~Potri.T024900.2[]~~Potri.T025000.1[]~~Potri.T025300.1[]~~Potri.T025500.1[]~~Potri.T025800.1[]~~Potri.T026200.1[]~~Potri.T026400.1[]~~Potri.T026600.1[]~~Potri.T026700.1[]~~Potri.T026800.1[]~~Potri.T026900.1[]~~Potri.T027200.1[]~~Potri.T028100.1[]~~Potri.T028300.1[]~~Potri.T028700.1[]~~Potri.T029000.1[]~~Potri.011G124100.1[]~~Potri.011G124400.1[]~~Potri.018G136300.1[]~~Potri.018G136700.1[]~~Potri.018G137900.1[]~~Potri.019G002800.1[]~~Potri.019G002800.2[]~~Potri.019G014500.1[]~~Potri.019G020200.1[]~~Potri.019G020200.2[]~~Potri.019G020500.1[]~~Potri.019G022800.1[]~~Potri.019G023300.1[]~~Potri.T052000.1[]~~Potri.T052600.1[]~~Potri.T053000.1[]~~Potri.T013300.2[]~~Potri.T013300.3[]~~Potri.T013300.1[]~~Potri.T014300.1[]~~Potri.T014900.1[]~~Potri.T015200.1[]~~Potri.T015900.1[]~~Potri.T015900.2[] |
| 3dSR-m0127_3p | 39 | Potri.T044600.1[]~~Potri.T044800.1[]~~Potri.T024700.1[]~~Potri.T024900.1[]~~Potri.T024900.2[]~~Potri.T025300.1[]~~Potri.T025500.1[]~~Potri.T025800.1[]~~Potri.T025900.1[]~~Potri.T026200.1[]~~Potri.T026400.1[]~~Potri.T026600.1[]~~Potri.T026700.1[]~~Potri.T026800.1[]~~Potri.T026900.1[]~~Potri.T027200.1[]~~Potri.T027300.1[]~~Potri.T027500.1[]~~Potri.T027700.1[]~~Potri.T028100.1[]~~Potri.T028500.1[]~~Potri.T028700.1[]~~Potri.T029000.1[]~~Potri.011G124000.1[]~~Potri.019G014500.1[]~~Potri.019G020200.1[]~~Potri.019G020200.2[]~~Potri.019G020500.1[]~~Potri.019G022800.1[]~~Potri.T052000.1[]~~Potri.T053000.1[]~~Potri.001G406000.1[]~~Potri.T012000.1[]~~Potri.T012900.1[]~~Potri.T013600.1[]~~Potri.T014700.1[]~~Potri.T015200.1[]~~Potri.T015900.1[]~~Potri.T015900.2[] |
