# Supplementary material for: Genome-wide analysis of salt-responsive and novel microRNAs in Populus euphratica by deep sequencing
Source: BMC Genet. 2014 Jun 20;15(Suppl 1):S6. doi: 10.1186/1471-2156-15-S1-S6 (PMC4118626; doi:10.1186/1471-2156-15-S1-S6)
Supplement: Additional file 6 — Summary of target genes of novel miRNAs from the leaf tissue. [file 1471-2156-15-S1-S6-S6.doc]

Additional file 6 - Summary of target genes of novel miRNAs from leaf tissue.

| MiRNA name | Targets number | Targets ID |
| --- | --- | --- |
| 3dSL-m0002_5p | 3 | Potri.005G007000.1[]~~Potri.001G379700.1[]~~Potri.001G379700.2[] |
| 3dSL-m0005_3p | 19 | Potri.003G190700.1[]~~Potri.011G104700.1[]~~Potri.011G104900.1[]~~Potri.011G105100.1[]~~Potri.002G048300.2[]~~Potri.002G048300.5[]~~Potri.002G048300.3[]~~Potri.002G048300.4[]~~Potri.002G048300.1[]~~Potri.T085700.2[]~~Potri.T085700.3[]~~Potri.T085700.1[]~~Potri.T086100.3[]~~Potri.T086100.1[]~~Potri.T086100.2[]~~Potri.001G029400.1[]~~Potri.001G034700.1[]~~Potri.001G034900.1[]~~Potri.001G035300.1[] |
| 3dSL-m0006_5p | 1 | Potri.001G455100.1[] |
| 3dSL-m0007_5p | 3 | Potri.003G175400.1[]~~Potri.001G298400.2[]~~Potri.001G298400.1[] |
| 3dSL-m0008_5p | 3 | Potri.003G175400.1[]~~Potri.001G298400.2[]~~Potri.001G298400.1[] |
| 3dSL-m0009_5p | 3 | Potri.003G175400.1[]~~Potri.001G298400.2[]~~Potri.001G298400.1[] |
| 3dSL-m0010_5p | 3 | Potri.003G175400.1[]~~Potri.001G298400.2[]~~Potri.001G298400.1[] |
| 3dSL-m0011_5p | 3 | Potri.003G175400.1[]~~Potri.001G298400.2[]~~Potri.001G298400.1[] |
| 3dSL-m0012_5p | 23 | Potri.004G164500.2[]~~Potri.004G164500.3[]~~Potri.004G164500.1[]~~Potri.003G034200.1[]~~Potri.003G175700.1[]~~Potri.011G122100.1[]~~Potri.011G122100.2[]~~Potri.011G122100.3[]~~Potri.011G155900.1[]~~Potri.018G021400.1[]~~Potri.018G021400.2[]~~Potri.016G054700.6[]~~Potri.016G122400.1[]~~Potri.016G141500.2[]~~Potri.002G130400.1[]~~Potri.014G036000.1[]~~Potri.001G052500.1[]~~Potri.001G459100.1[]~~Potri.012G035900.1[]~~Potri.012G035900.3[]~~Potri.012G035900.4[]~~Potri.012G035900.2[]~~Potri.012G036100.1[] |
| 3dSL-m0013_3p | 2 | Potri.009G067800.1[]~~Potri.009G067800.2[] |
| 3dSL-m0014_5p | 6 | Potri.016G096700.1[]~~Potri.005G151500.2[]~~Potri.005G151500.3[]~~Potri.005G151500.1[]~~Potri.005G219100.1[]~~Potri.014G126700.1[] |
| 3dSL-m0015_5p | 1 | Potri.001G386900.1[] |
| 3dSL-m0017_3p | 2 | Potri.005G071000.2[]~~Potri.005G071000.1[] |
| 3dSL-m0018_5p | 1 | Potri.001G455100.1[] |
| 3dSL-m0019_5p | 2 | Potri.006G170700.2[]~~Potri.006G170700.1[] |
| 3dSL-m0021_5p | 1 | Potri.011G024800.1[] |
| 3dSL-m0022_3p | 3 | Potri.019G045400.1[]~~Potri.019G082800.1[]~~Potri.014G033300.1[] |
| 3dSL-m0024_3p | 2 | Potri.016G060900.1[]~~Potri.016G113200.1[] |
| 3dSL-m0025_3p | 5 | Potri.011G042500.1[]~~Potri.018G033600.1[]~~Potri.008G189200.1[]~~Potri.001G300400.1[]~~Potri.001G300400.2[] |
| 3dSL-m0026_3p | 5 | Potri.004G222000.1[]~~Potri.003G190300.1[]~~Potri.003G190300.2[]~~Potri.003G190400.1[]~~Potri.003G190700.1[] |
| 3dSL-m0027_5p | 2 | Potri.004G020000.1[]~~Potri.004G020000.2[] |
| 3dSL-m0029_5p | 2 | Potri.004G215900.1[]~~Potri.001G000600.1[] |
| 3dSL-m0032_3p | 11 | Potri.007G048800.1[]~~Potri.007G048800.2[]~~Potri.001G337400.9[]~~Potri.001G337400.4[]~~Potri.001G337400.5[]~~Potri.001G337400.6[]~~Potri.001G337400.7[]~~Potri.001G337400.8[]~~Potri.001G337400.3[]~~Potri.001G337400.1[]~~Potri.001G337400.2[] |
| 3dSL-m0034_3p | 51 | Potri.009G056900.1[]~~Potri.009G058700.1[]~~Potri.004G066200.1[]~~Potri.004G066200.2[]~~Potri.004G066200.3[]~~Potri.003G012800.1[]~~Potri.003G013200.1[]~~Potri.003G013200.2[]~~Potri.003G013700.1[]~~Potri.003G025100.1[]~~Potri.003G027200.1[]~~Potri.003G027600.1[]~~Potri.003G028200.1[]~~Potri.003G028700.1[]~~Potri.003G029200.1[]~~Potri.003G029400.6[]~~Potri.003G029400.4[]~~Potri.003G029400.5[]~~Potri.003G029400.7[]~~Potri.003G029400.2[]~~Potri.003G029400.3[]~~Potri.003G029400.1[]~~Potri.T092800.1[]~~Potri.018G117400.1[]~~Potri.005G006700.1[]~~Potri.005G008600.1[]~~Potri.005G008800.1[]~~Potri.005G009300.1[]~~Potri.005G013200.1[]~~Potri.005G013800.1[]~~Potri.005G013900.1[]~~Potri.005G016200.1[]~~Potri.005G016800.1[]~~Potri.005G016900.1[]~~Potri.008G117000.2[]~~Potri.008G117000.1[]~~Potri.001G473300.2[]~~Potri.001G473300.1[]~~Potri.T040800.1[]~~Potri.T040800.3[]~~Potri.T040800.2[]~~Potri.T040800.4[]~~Potri.T041300.1[]~~Potri.T041300.2[]~~Potri.T041300.3[]~~Potri.T041700.1[]~~Potri.T041700.2[]~~Potri.T155600.1[]~~Potri.T155700.1[]~~Potri.T155700.2[]~~Potri.T155700.3[] |
| 3dSL-m0035_3p | 51 | Potri.009G056900.1[]~~Potri.009G058700.1[]~~Potri.004G066200.1[]~~Potri.004G066200.2[]~~Potri.004G066200.3[]~~Potri.003G012800.1[]~~Potri.003G013200.1[]~~Potri.003G013200.2[]~~Potri.003G013700.1[]~~Potri.003G025100.1[]~~Potri.003G027200.1[]~~Potri.003G027600.1[]~~Potri.003G028200.1[]~~Potri.003G028700.1[]~~Potri.003G029200.1[]~~Potri.003G029400.6[]~~Potri.003G029400.4[]~~Potri.003G029400.5[]~~Potri.003G029400.7[]~~Potri.003G029400.2[]~~Potri.003G029400.3[]~~Potri.003G029400.1[]~~Potri.T092800.1[]~~Potri.018G117400.1[]~~Potri.005G006700.1[]~~Potri.005G008600.1[]~~Potri.005G008800.1[]~~Potri.005G009300.1[]~~Potri.005G013200.1[]~~Potri.005G013800.1[]~~Potri.005G013900.1[]~~Potri.005G016200.1[]~~Potri.005G016800.1[]~~Potri.005G016900.1[]~~Potri.008G117000.2[]~~Potri.008G117000.1[]~~Potri.001G473300.2[]~~Potri.001G473300.1[]~~Potri.T040800.1[]~~Potri.T040800.3[]~~Potri.T040800.2[]~~Potri.T040800.4[]~~Potri.T041300.1[]~~Potri.T041300.2[]~~Potri.T041300.3[]~~Potri.T041700.1[]~~Potri.T041700.2[]~~Potri.T155600.1[]~~Potri.T155700.1[]~~Potri.T155700.2[]~~Potri.T155700.3[] |
| 3dSL-m0036_5p | 2 | Potri.005G014500.1[]~~Potri.005G017600.2[] |
| 3dSL-m0037_3p | 6 | Potri.017G100800.1[]~~Potri.017G101000.1[]~~Potri.004G113800.1[]~~Potri.004G113800.2[]~~Potri.004G113900.1[]~~Potri.008G069900.1[] |
| 3dSL-m0038_5p | 67 | Potri.004G060200.1[]~~Potri.013G024300.1[]~~Potri.003G012800.1[]~~Potri.003G013200.1[]~~Potri.003G013200.2[]~~Potri.003G013700.1[]~~Potri.003G025100.1[]~~Potri.003G027200.1[]~~Potri.003G027600.1[]~~Potri.003G028200.1[]~~Potri.003G028700.1[]~~Potri.003G029200.1[]~~Potri.003G029400.6[]~~Potri.003G029400.4[]~~Potri.003G029400.5[]~~Potri.003G029400.7[]~~Potri.003G029400.2[]~~Potri.003G029400.3[]~~Potri.003G029400.1[]~~Potri.003G184100.1[]~~Potri.T133400.1[]~~Potri.T133400.2[]~~Potri.006G237400.2[]~~Potri.006G237400.1[]~~Potri.006G237400.3[]~~Potri.018G124400.1[]~~Potri.018G124400.2[]~~Potri.T094900.1[]~~Potri.007G046900.2[]~~Potri.007G046900.1[]~~Potri.002G251700.1[]~~Potri.019G024700.1[]~~Potri.019G059600.1[]~~Potri.005G006700.1[]~~Potri.005G007600.1[]~~Potri.005G007900.1[]~~Potri.005G008600.1[]~~Potri.005G008800.1[]~~Potri.005G009300.1[]~~Potri.005G009700.1[]~~Potri.005G011600.1[]~~Potri.005G012100.1[]~~Potri.005G013200.1[]~~Potri.005G013400.1[]~~Potri.005G013700.1[]~~Potri.005G013800.1[]~~Potri.005G015700.1[]~~Potri.005G016100.1[]~~Potri.005G016200.1[]~~Potri.005G016700.1[]~~Potri.005G016800.1[]~~Potri.008G034000.1[]~~Potri.008G034000.2[]~~Potri.T040800.1[]~~Potri.T040800.3[]~~Potri.T040800.2[]~~Potri.T040800.4[]~~Potri.T041300.1[]~~Potri.T041300.2[]~~Potri.T041300.3[]~~Potri.T041700.1[]~~Potri.T041700.2[]~~Potri.T042200.1[]~~Potri.T155600.1[]~~Potri.T155700.1[]~~Potri.T155700.2[]~~Potri.T155700.3[] |
| 3dSL-m0039_5p | 2 | Potri.005G014500.1[]~~Potri.005G017600.2[] |
| 3dSL-m0040_3p | 1 | Potri.004G011700.1[] |
| 3dSL-m0041_5p | 1 | Potri.008G033400.1[] |
| 3dSL-m0042_3p | 6 | Potri.009G014500.1[]~~Potri.009G014500.2[]~~Potri.009G014500.3[]~~Potri.009G014500.4[]~~Potri.002G174100.2[]~~Potri.002G174100.1[] |
| 3dSL-m0044_5p | 3 | Potri.008G168100.1[]~~Potri.008G168100.2[]~~Potri.008G168100.3[] |
| 3dSL-m0045_3p | 3 | Potri.014G098700.1[]~~Potri.008G133200.1[]~~Potri.008G133200.2[] |
| 3dSL-m0051_5p | 1 | Potri.016G026300.1[] |
| 3dSL-m0052_3p | 1 | Potri.003G188400.1[] |
| 3dSL-m0053_5p | 2 | Potri.006G133300.1[]~~Potri.006G133300.2[] |
| 3dSL-m0055_5p | 1 | Potri.001G455100.1[] |
| 3dSL-m0056_5p | 4 | Potri.017G126900.1[]~~Potri.004G048600.2[]~~Potri.004G048600.3[]~~Potri.004G048600.1[] |
| 3dSL-m0057_5p | 2 | Potri.019G062400.1[]~~Potri.008G004400.1[] |
| 3dSL-m0061_5p | 2 | Potri.004G123500.1[]~~Potri.008G004400.1[] |
| 3dSL-m0062_3p | 2 | Potri.011G108200.1[]~~Potri.011G108300.1[] |
| 3dSL-m0063_5p | 12 | Potri.009G072900.3[]~~Potri.009G072900.2[]~~Potri.009G072900.1[]~~Potri.013G080100.1[]~~Potri.015G105400.1[]~~Potri.014G006800.1[]~~Potri.001G144000.2[]~~Potri.001G144000.1[]~~Potri.001G144000.4[]~~Potri.001G144000.3[]~~Potri.001G144000.5[]~~Potri.001G278400.1[] |
| 3dSL-m0064_5p | 3 | Potri.003G175400.1[]~~Potri.001G298400.2[]~~Potri.001G298400.1[] |
| 3dSL-m0065_5p | 12 | Potri.010G177400.3[]~~Potri.010G177400.2[]~~Potri.010G177400.1[]~~Potri.011G108000.2[]~~Potri.011G108000.3[]~~Potri.011G108000.1[]~~Potri.002G241100.1[]~~Potri.001G122100.1[]~~Potri.012G022700.3[]~~Potri.012G022700.4[]~~Potri.012G022700.2[]~~Potri.012G022700.1[] |
| 3dSL-m0066_3p | 14 | Potri.011G047300.1[]~~Potri.011G108200.1[]~~Potri.011G108300.1[]~~Potri.001G387900.1[]~~Potri.001G388100.1[]~~Potri.001G388200.1[]~~Potri.001G388300.1[]~~Potri.001G388400.1[]~~Potri.001G388600.1[]~~Potri.001G388800.1[]~~Potri.001G388800.2[]~~Potri.T061900.1[]~~Potri.T062100.1[]~~Potri.T062200.1[] |
| 3dSL-m0067_5p | 1 | Potri.010G120000.2[] |
| 3dSL-m0068_5p | 8 | Potri.009G009100.1[]~~Potri.007G048700.1[]~~Potri.007G048700.2[]~~Potri.002G241100.1[]~~Potri.005G098500.1[]~~Potri.005G098500.3[]~~Potri.005G098500.2[]~~Potri.001G242700.1[] |
| 3dSL-m0070_3p | 25 | Potri.017G103500.1[]~~Potri.017G103500.2[]~~Potri.017G104300.1[]~~Potri.017G104300.2[]~~Potri.T037600.1[]~~Potri.T037900.1[]~~Potri.T038300.1[]~~Potri.T039300.1[]~~Potri.T039900.1[]~~Potri.T068700.1[]~~Potri.011G008600.1[]~~Potri.011G008800.1[]~~Potri.011G009400.1[]~~Potri.011G011900.1[]~~Potri.011G012000.1[]~~Potri.011G012900.1[]~~Potri.011G013800.1[]~~Potri.011G015300.1[]~~Potri.011G060400.1[]~~Potri.011G060400.2[]~~Potri.T074000.1[]~~Potri.T074300.1[]~~Potri.019G069200.2[]~~Potri.019G069200.1[]~~Potri.001G028700.1[] |
| 3dSL-m0071_5p | 1 | Potri.011G095300.1[] |
| 3dSL-m0073_5p | 1 | Potri.006G219200.1[] |
| 3dSL-m0077_3p | 7 | Potri.010G121500.1[]~~Potri.010G121500.2[]~~Potri.T038300.1[]~~Potri.T039800.1[]~~Potri.001G128400.1[]~~Potri.001G338000.2[]~~Potri.001G338000.1[] |
| 3dSL-m0078_5p | 8 | Potri.005G247900.8[]~~Potri.005G247900.2[]~~Potri.005G247900.3[]~~Potri.005G247900.7[]~~Potri.005G247900.1[]~~Potri.005G247900.4[]~~Potri.005G247900.5[]~~Potri.005G247900.6[] |
| 3dSL-m0080_5p | 1 | Potri.008G033400.1[] |
| 3dSL-m0081_5p | 1 | Potri.008G033400.1[] |
| 3dSL-m0083_5p | 52 | Potri.010G199300.1[]~~Potri.017G102900.2[]~~Potri.017G102900.1[]~~Potri.017G103100.1[]~~Potri.017G103300.2[]~~Potri.017G103300.1[]~~Potri.017G103500.1[]~~Potri.017G103500.2[]~~Potri.017G103800.1[]~~Potri.017G103900.2[]~~Potri.017G103900.1[]~~Potri.017G104000.1[]~~Potri.017G104300.1[]~~Potri.017G104300.2[]~~Potri.017G104700.1[]~~Potri.017G105000.2[]~~Potri.017G105000.1[]~~Potri.017G105500.2[]~~Potri.017G105500.3[]~~Potri.017G105500.1[]~~Potri.013G097000.1[]~~Potri.013G097300.2[]~~Potri.013G097300.3[]~~Potri.013G097300.1[]~~Potri.013G097800.1[]~~Potri.013G097900.1[]~~Potri.013G098000.1[]~~Potri.013G098500.1[]~~Potri.006G174900.1[]~~Potri.T154200.3[]~~Potri.T154200.2[]~~Potri.T154200.1[]~~Potri.T073600.1[]~~Potri.T073800.1[]~~Potri.T074000.1[]~~Potri.T074200.1[]~~Potri.T074300.1[]~~Potri.014G063300.2[]~~Potri.014G063300.3[]~~Potri.014G063300.1[]~~Potri.014G063500.1[]~~Potri.014G063900.2[]~~Potri.014G063900.1[]~~Potri.014G064100.1[]~~Potri.014G064300.1[]~~Potri.014G064800.2[]~~Potri.014G064800.1[]~~Potri.T129000.1[]~~Potri.T129200.2[]~~Potri.T129200.1[]~~Potri.T129300.1[]~~Potri.T129400.1[] |
| 3dSL-m0084_5p | 5 | Potri.011G042500.1[]~~Potri.018G033600.1[]~~Potri.008G189200.1[]~~Potri.001G300400.1[]~~Potri.001G300400.2[] |
| 3dSL-m0085_5p | 1 | Potri.001G455100.1[] |
| 3dSL-m0086_5p | 1 | Potri.001G455100.1[] |
| 3dSL-m0090_3p | 3 | Potri.005G133000.1[]~~Potri.005G133400.1[]~~Potri.008G224300.1[] |
| 3dSL-m0091_3p | 1 | Potri.006G219200.1[] |
| 3dSL-m0093_5p | 2 | Potri.004G013800.1[]~~Potri.007G005200.1[] |
| 3dSL-m0094_5p | 3 | Potri.015G091200.1[]~~Potri.008G138700.1[]~~Potri.012G093900.1[] |
| 3dSL-m0095_5p | 1 | Potri.013G102700.1[] |
| 3dSL-m0097_3p | 2 | Potri.002G174100.2[]~~Potri.002G174100.1[] |
| 3dSL-m0098_5p | 3 | Potri.007G122800.1[]~~Potri.007G122800.2[]~~Potri.014G193900.1[] |
| 3dSL-m0099_5p | 4 | Potri.006G106300.1[]~~Potri.002G068900.1[]~~Potri.019G035600.2[]~~Potri.019G035600.1[] |
| 3dSL-m0102_5p | 7 | Potri.013G006000.5[]~~Potri.013G006000.2[]~~Potri.013G006000.3[]~~Potri.013G006000.1[]~~Potri.013G006000.4[]~~Potri.006G072900.3[]~~Potri.006G072900.2[] |
| 3dSL-m0105_3p | 8 | Potri.017G101000.1[]~~Potri.004G113800.1[]~~Potri.004G113800.2[]~~Potri.004G113900.1[]~~Potri.018G099000.2[]~~Potri.018G099000.3[]~~Potri.018G099000.1[]~~Potri.001G420800.1[] |
| 3dSL-m0107_5p | 12 | Potri.009G072900.3[]~~Potri.009G072900.2[]~~Potri.009G072900.1[]~~Potri.013G080100.1[]~~Potri.015G105400.1[]~~Potri.014G006800.1[]~~Potri.001G144000.2[]~~Potri.001G144000.1[]~~Potri.001G144000.4[]~~Potri.001G144000.3[]~~Potri.001G144000.5[]~~Potri.001G278400.1[] |
| 3dSL-m0110_3p | 4 | Potri.011G147400.1[]~~Potri.011G147400.2[]~~Potri.002G247400.1[]~~Potri.019G036700.6[] |
| 3dSL-m0113_5p | 11 | Potri.010G063100.1[]~~Potri.004G065900.1[]~~Potri.004G065900.3[]~~Potri.004G065900.2[]~~Potri.003G144900.2[]~~Potri.003G144900.1[]~~Potri.002G065500.1[]~~Potri.002G137700.2[]~~Potri.002G137700.3[]~~Potri.002G137700.1[]~~Potri.008G116000.1[] |
| 3dSL-m0114_5p | 9 | Potri.006G201900.4[]~~Potri.006G201900.3[]~~Potri.006G201900.1[]~~Potri.006G201900.2[]~~Potri.006G201900.5[]~~Potri.016G068200.3[]~~Potri.016G068200.1[]~~Potri.016G068200.2[]~~Potri.015G061600.1[] |
| 3dSL-m0116_5p | 2 | Potri.004G123500.1[]~~Potri.008G004400.1[] |
| 3dSL-m0117_3p | 2 | Potri.001G403500.1[]~~Potri.001G403500.2[] |
| 3dSL-m0118_3p | 18 | Potri.009G015000.6[]~~Potri.009G015000.1[]~~Potri.009G015000.2[]~~Potri.009G015000.4[]~~Potri.009G015000.5[]~~Potri.009G015000.3[]~~Potri.004G210200.1[]~~Potri.004G210200.3[]~~Potri.004G210200.2[]~~Potri.004G210200.4[]~~Potri.004G210200.5[]~~Potri.004G210200.6[]~~Potri.007G043300.1[]~~Potri.007G043300.2[]~~Potri.002G037100.1[]~~Potri.001G226900.1[]~~Potri.012G111800.2[]~~Potri.012G111800.1[] |
| 3dSL-m0119_3p | 1 | Potri.006G081600.1[] |
| 3dSL-m0120_3p | 3 | Potri.003G171700.1[]~~Potri.003G171700.4[]~~Potri.003G171700.5[] |
| 3dSL-m0122_3p | 3 | Potri.013G051600.1[]~~Potri.013G051700.1[]~~Potri.018G005200.1[] |
| 3dSL-m0124_3p | 18 | Potri.009G015000.6[]~~Potri.009G015000.1[]~~Potri.009G015000.2[]~~Potri.009G015000.4[]~~Potri.009G015000.5[]~~Potri.009G015000.3[]~~Potri.004G210200.1[]~~Potri.004G210200.3[]~~Potri.004G210200.2[]~~Potri.004G210200.4[]~~Potri.004G210200.5[]~~Potri.004G210200.6[]~~Potri.007G043300.1[]~~Potri.007G043300.2[]~~Potri.002G037100.1[]~~Potri.001G226900.1[]~~Potri.012G111800.2[]~~Potri.012G111800.1[] |
| 3dSL-m0125_3p | 32 | Potri.017G010800.1[]~~Potri.017G010800.2[]~~Potri.017G011800.2[]~~Potri.017G011800.1[]~~Potri.017G011800.3[]~~Potri.017G011800.4[]~~Potri.019G068200.1[]~~Potri.019G068300.1[]~~Potri.019G095900.1[]~~Potri.019G097100.2[]~~Potri.019G097100.1[]~~Potri.019G097500.1[]~~Potri.019G097500.1[]~~Potri.019G097800.1[]~~Potri.019G097800.1[]~~Potri.019G097800.2[]~~Potri.019G097800.2[]~~Potri.019G098500.2[]~~Potri.019G098500.1[]~~Potri.019G098700.1[]~~Potri.019G098900.1[]~~Potri.019G112600.1[]~~Potri.019G113700.2[]~~Potri.019G113700.2[]~~Potri.019G113700.1[]~~Potri.019G113700.1[]~~Potri.019G114600.1[]~~Potri.019G114600.2[]~~Potri.019G114600.3[]~~Potri.019G114800.1[]~~Potri.019G114800.2[]~~Potri.005G183600.1[] |
| 3dSL-m0126_3p | 32 | Potri.017G010800.1[]~~Potri.017G010800.2[]~~Potri.017G011800.2[]~~Potri.017G011800.1[]~~Potri.017G011800.3[]~~Potri.017G011800.4[]~~Potri.019G068200.1[]~~Potri.019G068300.1[]~~Potri.019G095900.1[]~~Potri.019G097100.2[]~~Potri.019G097100.1[]~~Potri.019G097500.1[]~~Potri.019G097500.1[]~~Potri.019G097800.1[]~~Potri.019G097800.1[]~~Potri.019G097800.2[]~~Potri.019G097800.2[]~~Potri.019G098500.2[]~~Potri.019G098500.1[]~~Potri.019G098700.1[]~~Potri.019G098900.1[]~~Potri.019G112600.1[]~~Potri.019G113700.2[]~~Potri.019G113700.2[]~~Potri.019G113700.1[]~~Potri.019G113700.1[]~~Potri.019G114600.1[]~~Potri.019G114600.2[]~~Potri.019G114600.3[]~~Potri.019G114800.1[]~~Potri.019G114800.2[]~~Potri.005G183600.1[] |
| 3dSL-m0127_3p | 32 | Potri.017G010800.1[]~~Potri.017G010800.2[]~~Potri.017G011800.2[]~~Potri.017G011800.1[]~~Potri.017G011800.3[]~~Potri.017G011800.4[]~~Potri.019G068200.1[]~~Potri.019G068300.1[]~~Potri.019G095900.1[]~~Potri.019G097100.2[]~~Potri.019G097100.1[]~~Potri.019G097500.1[]~~Potri.019G097500.1[]~~Potri.019G097800.1[]~~Potri.019G097800.1[]~~Potri.019G097800.2[]~~Potri.019G097800.2[]~~Potri.019G098500.2[]~~Potri.019G098500.1[]~~Potri.019G098700.1[]~~Potri.019G098900.1[]~~Potri.019G112600.1[]~~Potri.019G113700.2[]~~Potri.019G113700.2[]~~Potri.019G113700.1[]~~Potri.019G113700.1[]~~Potri.019G114600.1[]~~Potri.019G114600.2[]~~Potri.019G114600.3[]~~Potri.019G114800.1[]~~Potri.019G114800.2[]~~Potri.005G183600.1[] |
| 3dSL-m0128_3p | 2 | Potri.009G119000.1[]~~Potri.001G191800.1[] |
| 3dSL-m0131_5p | 42 | Potri.009G027600.2[]~~Potri.009G027600.3[]~~Potri.009G027600.1[]~~Potri.T003600.1[]~~Potri.T003800.1[]~~Potri.T003800.1[]~~Potri.T003800.1[]~~Potri.T003900.1[]~~Potri.T003900.1[]~~Potri.T004100.1[]~~Potri.T004100.1[]~~Potri.T004100.1[]~~Potri.T004100.1[]~~Potri.T004200.1[]~~Potri.T004200.1[]~~Potri.004G197600.1[]~~Potri.T044800.1[]~~Potri.T045200.1[]~~Potri.T045300.2[]~~Potri.T045300.1[]~~Potri.T046000.1[]~~Potri.018G135400.1[]~~Potri.018G135600.1[]~~Potri.018G136300.1[]~~Potri.018G136500.1[]~~Potri.018G136700.1[]~~Potri.018G137900.1[]~~Potri.019G002800.1[]~~Potri.019G002800.2[]~~Potri.T012000.1[]~~Potri.T012000.1[]~~Potri.T012000.1[]~~Potri.T012000.1[]~~Potri.T012000.1[]~~Potri.T013600.1[]~~Potri.T013800.1[]~~Potri.T013800.1[]~~Potri.T014600.1[]~~Potri.T014900.1[]~~Potri.T015900.1[]~~Potri.T015900.1[]~~Potri.T015900.2[] |
| 3dSL-m0133_3p | 19 | Potri.T001400.1[]~~Potri.T001500.1[]~~Potri.T001700.1[]~~Potri.T001900.1[]~~Potri.T002200.1[]~~Potri.T002300.2[]~~Potri.T002300.1[]~~Potri.T002400.1[]~~Potri.T002500.1[]~~Potri.T002600.1[]~~Potri.T002900.3[]~~Potri.T002900.2[]~~Potri.T002900.1[]~~Potri.T003000.1[]~~Potri.003G014200.2[]~~Potri.003G014200.1[]~~Potri.019G046000.1[]~~Potri.001G066500.1[]~~Potri.T112700.1[] |
| 3dSL-m0134_5p | 24 | Potri.T001600.1[]~~Potri.T001700.1[]~~Potri.T001900.1[]~~Potri.T002600.1[]~~Potri.T002900.3[]~~Potri.T002900.2[]~~Potri.T002900.1[]~~Potri.T003000.1[]~~Potri.T003000.2[]~~Potri.T005100.1[]~~Potri.T005200.1[]~~Potri.T005500.1[]~~Potri.003G014200.2[]~~Potri.003G014200.1[]~~Potri.006G023400.1[]~~Potri.006G023400.2[]~~Potri.006G023400.4[]~~Potri.016G021400.1[]~~Potri.016G021400.2[]~~Potri.016G021900.1[]~~Potri.016G021900.2[]~~Potri.019G046000.1[]~~Potri.019G097200.1[]~~Potri.019G114300.1[] |
| 3dSL-m0136_3p | 39 | Potri.T044600.1[]~~Potri.T044800.1[]~~Potri.T024700.1[]~~Potri.T024900.1[]~~Potri.T024900.2[]~~Potri.T025300.1[]~~Potri.T025500.1[]~~Potri.T025800.1[]~~Potri.T025900.1[]~~Potri.T026200.1[]~~Potri.T026400.1[]~~Potri.T026600.1[]~~Potri.T026700.1[]~~Potri.T026800.1[]~~Potri.T026900.1[]~~Potri.T027200.1[]~~Potri.T027300.1[]~~Potri.T027500.1[]~~Potri.T027700.1[]~~Potri.T028100.1[]~~Potri.T028500.1[]~~Potri.T028700.1[]~~Potri.T029000.1[]~~Potri.011G124000.1[]~~Potri.019G014500.1[]~~Potri.019G020200.1[]~~Potri.019G020200.2[]~~Potri.019G020500.1[]~~Potri.019G022800.1[]~~Potri.T052000.1[]~~Potri.T053000.1[]~~Potri.001G406000.1[]~~Potri.T012000.1[]~~Potri.T012900.1[]~~Potri.T013600.1[]~~Potri.T014700.1[]~~Potri.T015200.1[]~~Potri.T015900.1[]~~Potri.T015900.2[] |
