# Supplementary material for: Genome-wide analysis of salt-responsive and novel microRNAs in Populus euphratica by deep sequencing
Source: BMC Genet. 2014 Jun 20;15(Suppl 1):S6. doi: 10.1186/1471-2156-15-S1-S6 (PMC4118626; doi:10.1186/1471-2156-15-S1-S6)
Supplement: Additional file 7 — Significant expression changes in conserved Populus euphratica miRNAs between the control leaf (3dCKL) and control root (3dCKR) libraries. [file 1471-2156-15-S1-S6-S7.doc]

Additional file 7 - Significantly expression changed of conserved miRNAs identified in *P. euphratica* between control leaf (3dCKL) and control root (3dCKR) libraries.

| pairwise | miR-name | 3dCKR-std | 3dCKL-std | fold-change(log2 3dCKL/3dCKR) | p-value | sig-lable |
| --- | --- | --- | --- | --- | --- | --- |
| 3dCKR-3dCKL | miR1310 | 103.8708 | 182.3145 | 0.81163916 | 1.61583477537993e-77 |  |
| 3dCKR-3dCKL | miR1444a | 5.0960 | 9.5495 | 0.90605992 | 2.97540494274615e-06 |  |
| 3dCKR-3dCKL | miR1446a | 148.3509 | 59.0447 | -1.32913421 | 5.66045668290829e-140 | ** |
| 3dCKR-3dCKL | miR1447 | 13.2748 | 35.8887 | 1.43483954 | 2.68439750153561e-39 | ** |
| 3dCKR-3dCKL | miR1448 | 66.1225 | 47.3731 | -0.48107319 | 1.85087157164328e-12 |  |
| 3dCKR-3dCKL | miR1450 | 214.2218 | 231.9346 | 0.11461276 | 0.000808342472571927 |  |
| 3dCKR-3dCKL | miR1508b | 20.6358 | 0.1248 | -7.36938762 | 6.81889303923873e-96 | ** |
| 3dCKR-3dCKL | miR1511 | 47.3742 | 35.9511 | -0.39806567 | 5.57829846654556e-07 |  |
| 3dCKR-3dCKL | miR1515 | 1.5099 | 0.4369 | -1.78907799 | 0.00195163359910629 | ** |
| 3dCKR-3dCKL | miR1520d | 5521.6366 | 2073.4300 | -1.41307660 | 0 | ** |
| 3dCKR-3dCKL | miR1523a | 22.9636 | 30.2089 | 0.39562482 | 7.12379870641186e-05 |  |
| 3dCKR-3dCKL | miR1535a | 44.2285 | 0.1872 | -7.88425206 | 1.06883559234554e-206 | ** |
| 3dCKR-3dCKL | miR156a | 19226.2298 | 3776.9256 | -2.34779161 | 0 | ** |
| 3dCKR-3dCKL | miR156b-3p | 69.8973 | 34.3907 | -1.02321825 | 1.05570299215649e-44 | ** |
| 3dCKR-3dCKL | miR157a | 42805.2191 | 273427.2469 | 2.67529840 | 0 | ** |
| 3dCKR-3dCKL | miR157d-3p | 3.3344 | 4.4315 | 0.41036793 | 0.117073374254198 |  |
| 3dCKR-3dCKL | miR159a | 223.9734 | 216.1435 | -0.05133795 | 0.135978192307851 |  |
| 3dCKR-3dCKL | miR160a | 14.0298 | 0.4993 | -4.81244373 | 2.33810765951277e-56 | ** |
| 3dCKR-3dCKL | miR160b-3p | 25.2914 | 12.3582 | -1.03317827 | 1.89271163373877e-17 | ** |
| 3dCKR-3dCKL | miR162a | 69.3311 | 135.5656 | 0.96741658 | 1.4079258142466e-77 |  |
| 3dCKR-3dCKL | miR164a | 2439.9261 | 4095.9293 | 0.74735336 | 0 |  |
| 3dCKR-3dCKL | miR165a | 87.7649 | 286.1109 | 1.70485848 | 0 | ** |
| 3dCKR-3dCKL | miR165a-3p | 51.2748 | 174.2006 | 1.76442773 | 1.90237167962513e-247 | ** |
| 3dCKR-3dCKL | miR166a | 17115.2175 | 49002.9155 | 1.51758796 | 0 | ** |
| 3dCKR-3dCKL | miR166h-3p | 4464.0543 | 2557.8961 | -0.80339691 | 0 |  |
| 3dCKR-3dCKL | miR167f-3p | 164.5198 | 59.9185 | -1.45718781 | 5.57183064954711e-178 | ** |
| 3dCKR-3dCKL | miR167h | 6635.2122 | 14439.2367 | 1.12177997 | 0 | ** |
| 3dCKR-3dCKL | miR168a | 5985.3119 | 3858.0653 | -0.63354887 | 0 |  |
| 3dCKR-3dCKL | miR168a-3p | 51.0232 | 42.7544 | -0.25508048 | 0.000644344282390669 |  |
| 3dCKR-3dCKL | miR169ac | 388.1786 | 931.5455 | 1.26290565 | 0 | ** |
| 3dCKR-3dCKL | miR169n-3p | 9.3742 | 0.5617 | -4.06082387 | 2.35487891448885e-34 | ** |
| 3dCKR-3dCKL | miR171b-3p | 1249.9100 | 316.2574 | -1.98265307 | 0 | ** |
| 3dCKR-3dCKL | miR171e | 60.0828 | 5.1805 | -3.53578879 | 2.39220382232333e-190 | ** |
| 3dCKR-3dCKL | miR172a | 104.0596 | 299.4677 | 1.52499034 | 0 | ** |
| 3dCKR-3dCKL | miR172a-3p | 102.1092 | 295.4731 | 1.53291393 | 0 | ** |
| 3dCKR-3dCKL | miR1886.2 | 16.4205 | 0.1248 | -7.03973631 | 8.36829077350982e-76 | ** |
| 3dCKR-3dCKL | miR2086-3p | 66.6258 | 2.5590 | -4.70242890 | 9.55742715975038e-259 | ** |
| 3dCKR-3dCKL | miR2089-3p | 26.6126 | 1.9973 | -3.73598652 | 3.70623982246092e-89 | ** |
| 3dCKR-3dCKL | miR2111a | 3.0199 | 0.9986 | -1.59652197 | 4.44163900010096e-05 | ** |
| 3dCKR-3dCKL | miR2119 | 7.5497 | 1.7476 | -2.11104431 | 3.84962085147646e-15 | ** |
| 3dCKR-3dCKL | miR2199 | 4418.5046 | 2256.9928 | -0.96915637 | 0 |  |
| 3dCKR-3dCKL | miR2604 | 124.8840 | 19.9728 | -2.64448015 | 1.03151101245165e-295 | ** |
| 3dCKR-3dCKL | miR2610a | 0.5033 | 4.4315 | 3.13830461 | 6.93646059906181e-14 | ** |
| 3dCKR-3dCKL | miR2630a | 7.8013 | 0.3121 | -4.64363429 | 2.71213915107914e-31 | ** |
| 3dCKR-3dCKL | miR2637 | 13.5265 | 0.1248 | -6.76002684 | 4.82758758966536e-62 | ** |
| 3dCKR-3dCKL | miR2651 | 15.7285 | 39.9457 | 1.34465912 | 1.2729977327938e-39 | ** |
| 3dCKR-3dCKL | miR2666 | 20.8245 | 0.2497 | -6.38194222 | 2.03915112306863e-93 | ** |
| 3dCKR-3dCKL | miR2911 | 4447.7596 | 815.6407 | -2.44707314 | 0 | ** |
| 3dCKR-3dCKL | miR2912a | 675.0030 | 1526.7985 | 1.17754386 | 0 | ** |
| 3dCKR-3dCKL | miR2913 | 7.1722 | 1.9973 | -1.84436467 | 2.47313654325861e-12 | ** |
| 3dCKR-3dCKL | miR2916 | 1036.5691 | 1499.5855 | 0.53274749 | 7.01919471787028e-297 |  |
| 3dCKR-3dCKL | miR2936 | 77.1324 | 0.8114 | -6.57077989 | 0 | ** |
| 3dCKR-3dCKL | miR2938 | 71.1556 | 34.0162 | -1.06475532 | 2.01276325237724e-48 | ** |
| 3dCKR-3dCKL | miR319b-5p | 1.5728 | 0.01 | -7.29719141 | 2.68672639714831e-08 | ** |
| 3dCKR-3dCKL | miR3434-3p | 21.0761 | 9.5495 | -1.14211083 | 3.89677243452321e-17 | ** |
| 3dCKR-3dCKL | miR3509-5p | 67.5066 | 16.0407 | -2.07329146 | 2.52812871894033e-120 | ** |
| 3dCKR-3dCKL | miR3626-5p | 5.1589 | 0.01 | -9.01091969 | 1.48525623087827e-25 | ** |
| 3dCKR-3dCKL | miR3627-5p | 5.2848 | 78.2061 | 3.88736036 | 3.28778557903018e-266 | ** |
| 3dCKR-3dCKL | miR3629a-3p | 0.6921 | 6.6784 | 3.27045010 | 5.18456725648892e-21 | ** |
| 3dCKR-3dCKL | miR390a | 40.9569 | 29.1479 | -0.49071458 | 1.64729300260137e-08 |  |
| 3dCKR-3dCKL | miR391 | 3.9007 | 60.5427 | 3.95614806 | 3.15092054197882e-209 | ** |
| 3dCKR-3dCKL | miR393a-3p | 2.8311 | 0.1872 | -3.91871037 | 5.87775139689238e-11 | ** |
| 3dCKR-3dCKL | miR393h | 7.5497 | 80.8900 | 3.42147014 | 7.93923575004844e-248 | ** |
| 3dCKR-3dCKL | miR3949 | 1.8245 | 11.1099 | 2.60627278 | 2.56823093833195e-27 | ** |
| 3dCKR-3dCKL | miR394b-3p | 94.7483 | 4.1818 | -4.50190409 | 0 | ** |
| 3dCKR-3dCKL | miR3954 | 189.9999 | 1.1859 | -7.32387250 | 0 | ** |
| 3dCKR-3dCKL | miR395a | 0.6291 | 15.0420 | 4.57956323 | 1.72026462795104e-58 | ** |
| 3dCKR-3dCKL | miR396a | 372.2614 | 232.5587 | -0.67872112 | 4.85732164146892e-115 |  |
| 3dCKR-3dCKL | miR396b-3p | 362.8873 | 457.8773 | 0.33543948 | 4.0353858180576e-40 |  |
| 3dCKR-3dCKL | miR397a | 29.0033 | 15.7286 | -0.88282680 | 1.41725819237147e-15 |  |
| 3dCKR-3dCKL | miR398c-5p | 1.9503 | 0.8738 | -1.15832105 | 0.0105121751059587 | * |
| 3dCKR-3dCKL | miR399f | 3.3344 | 1.3107 | -1.34708967 | 0.000153843110046751 | ** |
| 3dCKR-3dCKL | miR403c-5p | 72.2880 | 84.2604 | 0.22109859 | 0.000131760253053857 |  |
| 3dCKR-3dCKL | miR408b | 447.1919 | 59.9809 | -2.89831898 | 0 | ** |
| 3dCKR-3dCKL | miR415 | 2.3278 | 0.1248 | -4.22127727 | 1.29311997101693e-09 | ** |
| 3dCKR-3dCKL | miR4343a | 1.0066 | 0.7490 | -0.42645288 | 0.445283395737332 |  |
| 3dCKR-3dCKL | miR4348 | 2.7682 | 2.8087 | 0.02095436 | 0.946221762518237 |  |
| 3dCKR-3dCKL | miR4399 | 53.9172 | 0.1248 | -8.75498381 | 3.17152646750537e-255 | ** |
| 3dCKR-3dCKL | miR4413a | 586.7348 | 0.1248 | -12.19887471 | 0 | ** |
| 3dCKR-3dCKL | miR4414b | 11.7649 | 3.1208 | -1.91450125 | 3.05095142754762e-20 | ** |
| 3dCKR-3dCKL | miR472b | 361.1257 | 256.5885 | -0.49304458 | 1.16782361015635e-63 |  |
| 3dCKR-3dCKL | miR473a-3p | 25.5430 | 11.6092 | -1.13765942 | 2.51295720961603e-20 | ** |
| 3dCKR-3dCKL | miR473a-5p | 92.8609 | 6.0543 | -3.93903919 | 8.57105082405395e-320 | ** |
| 3dCKR-3dCKL | miR475a-3p | 53.5397 | 24.9036 | -1.10425475 | 5.30712940839205e-39 | ** |
| 3dCKR-3dCKL | miR475a-5p | 82.9205 | 179.5059 | 1.11423054 | 3.11710753780795e-128 | ** |
| 3dCKR-3dCKL | miR477a-3p | 28.8146 | 1.4355 | -4.32717476 | 3.44658199226073e-107 | ** |
| 3dCKR-3dCKL | miR477a-5p | 153.8244 | 9.7992 | -3.97247658 | 0 | ** |
| 3dCKR-3dCKL | miR479 | 36.1755 | 17.4762 | -1.04962144 | 6.93771796307613e-25 | ** |
| 3dCKR-3dCKL | miR482a | 67.6953 | 48.9334 | -0.46823614 | 3.55493744607598e-12 |  |
| 3dCKR-3dCKL | miR482c-3p | 33.1556 | 16.3528 | -1.01971488 | 5.95735654085057e-22 | ** |
| 3dCKR-3dCKL | miR4993 | 2.1391 | 7.3650 | 1.78368160 | 4.16326508256203e-12 | ** |
| 3dCKR-3dCKL | miR4995 | 14.4702 | 13.7313 | -0.07561664 | 0.578639443868597 |  |
| 3dCKR-3dCKL | miR5020b | 46.9338 | 91.3133 | 0.96019773 | 3.27634779814543e-52 |  |
| 3dCKR-3dCKL | miR5021 | 10.5066 | 36.7001 | 1.80448811 | 3.22790572869792e-55 | ** |
| 3dCKR-3dCKL | miR5037c | 75.9371 | 12.0461 | -2.65623876 | 1.88030997143308e-181 | ** |
| 3dCKR-3dCKL | miR5139 | 24.6622 | 5.7422 | -2.10262602 | 7.54692446894516e-46 | ** |
| 3dCKR-3dCKL | miR5140 | 17.1755 | 0.1872 | -6.51962786 | 9.90063258380145e-78 | ** |
| 3dCKR-3dCKL | miR5210 | 30.6391 | 22.7815 | -0.42751118 | 1.7077127522738e-05 |  |
| 3dCKR-3dCKL | miR5218 | 56.6854 | 82.9497 | 0.54925956 | 5.49509542998852e-19 |  |
| 3dCKR-3dCKL | miR5221 | 374.7151 | 0.4369 | -9.74427527 | 0 | ** |
| 3dCKR-3dCKL | miR5224b | 146.5893 | 81.8262 | -0.84114504 | 1.40831294860454e-66 |  |
| 3dCKR-3dCKL | miR5227 | 0.8179 | 1.1235 | 0.45800376 | 0.389438216454741 |  |
| 3dCKR-3dCKL | miR5229a | 2.4536 | 0.8738 | -1.48952506 | 0.000480847549081854 | ** |
| 3dCKR-3dCKL | miR5230 | 539.0461 | 1096.5711 | 1.02451879 | 0 | ** |
| 3dCKR-3dCKL | miR5234 | 2.7682 | 0.3745 | -2.88591056 | 1.56654764289175e-08 | ** |
| 3dCKR-3dCKL | miR5239 | 1.1324 | 1.9973 | 0.81866739 | 0.0522202744657864 |  |
| 3dCKR-3dCKL | miR5248 | 1.1954 | 5.6798 | 2.24834668 | 1.50405200736677e-12 | ** |
| 3dCKR-3dCKL | miR5255 | 12.3940 | 29.0230 | 1.22755478 | 1.36593417317972e-25 | ** |
| 3dCKR-3dCKL | miR5260 | 16.1060 | 15.6038 | -0.04570083 | 0.721779780200866 |  |
| 3dCKR-3dCKL | miR5263 | 1.6987 | 0.2497 | -2.76616336 | 1.75182609464444e-05 | ** |
| 3dCKR-3dCKL | miR5265 | 4361.8192 | 1265.4664 | -1.78526077 | 0 | ** |
| 3dCKR-3dCKL | miR529 | 9.3742 | 41.6933 | 2.15304808 | 1.73565963791467e-78 | ** |
| 3dCKR-3dCKL | miR5296 | 1.6987 | 1.5604 | -0.12251518 | 0.761572423754538 |  |
| 3dCKR-3dCKL | miR5298b | 1.6987 | 9.2999 | 2.45278412 | 1.16593429080132e-21 | ** |
| 3dCKR-3dCKL | miR5301 | 85.8774 | 28.0868 | -1.61238625 | 1.35421454094321e-108 | ** |
| 3dCKR-3dCKL | miR530b | 0.6921 | 5.0556 | 2.86882991 | 1.87916669392713e-14 | ** |
| 3dCKR-3dCKL | miR535a | 2.8940 | 4.8684 | 0.75038279 | 0.00462746399352428 |  |
| 3dCKR-3dCKL | miR5368 | 36.8675 | 15.5414 | -1.24623312 | 7.72588433454441e-33 | ** |
| 3dCKR-3dCKL | miR5373 | 7.9901 | 11.1099 | 0.47556037 | 0.00434474154137347 |  |
| 3dCKR-3dCKL | miR5561-5p | 1.5728 | 3.0583 | 0.95939471 | 0.00580941736165382 |  |
| 3dCKR-3dCKL | miR5562-3p | 3.3973 | 4.6811 | 0.46245896 | 0.0719708304310578 |  |
| 3dCKR-3dCKL | miR5632 | 5.5993 | 1.7476 | -1.67987147 | 6.93528259987397e-09 | ** |
| 3dCKR-3dCKL | miR5646 | 361.2515 | 136.4394 | -1.40474326 | 0 | ** |
| 3dCKR-3dCKL | miR5647 | 11.1987 | 0.1248 | -6.48756952 | 5.30600210784233e-51 | ** |
| 3dCKR-3dCKL | miR5652 | 1.5099 | 1.1859 | -0.34847064 | 0.435604973794809 |  |
| 3dCKR-3dCKL | miR5656 | 3.8377 | 3.9322 | 0.03509476 | 0.893255055644953 |  |
| 3dCKR-3dCKL | miR5658 | 102.3609 | 6.3039 | -4.02127628 | 0 | ** |
| 3dCKR-3dCKL | miR5665 | 6.7947 | 2.2469 | -1.59647394 | 7.37344545730805e-10 | ** |
| 3dCKR-3dCKL | miR5671 | 657.1984 | 302.5885 | -1.11897181 | 0 | ** |
| 3dCKR-3dCKL | miR5672 | 1.2583 | 0.1248 | -3.33378609 | 6.11774335288696e-05 | ** |
| 3dCKR-3dCKL | miR5755 | 7.8013 | 13.4192 | 0.78251221 | 1.0022530973746e-06 |  |
| 3dCKR-3dCKL | miR5772 | 48.0662 | 182.1273 | 1.92185253 | 1.91182676267998e-290 | ** |
| 3dCKR-3dCKL | miR5776 | 1.0695 | 0.1872 | -2.51428414 | 0.00140126907759152 | ** |
| 3dCKR-3dCKL | miR6022 | 51.0861 | 0.0624 | -9.67716904 | 5.85521184458132e-244 | ** |
| 3dCKR-3dCKL | miR6028 | 2.5166 | 0.1248 | -4.33378609 | 1.84235279551864e-10 | ** |
| 3dCKR-3dCKL | miR6032 | 2.7053 | 0.01 | -8.07964478 | 9.53918371720168e-14 | ** |
| 3dCKR-3dCKL | miR6035 | 10.3179 | 0.6242 | -4.04699720 | 1.36687505948429e-37 | ** |
| 3dCKR-3dCKL | miR6103-3p | 13.0861 | 0.9986 | -3.71198448 | 2.21841884411771e-44 | ** |
| 3dCKR-3dCKL | miR6145e | 1.8245 | 14.8548 | 3.02535613 | 6.75947078927083e-42 | ** |
| 3dCKR-3dCKL | miR6171 | 27.9967 | 57.6091 | 1.04103993 | 3.96483989607024e-38 | ** |
| 3dCKR-3dCKL | miR6173 | 38.1887 | 13.7937 | -1.46913631 | 3.32605806968363e-43 | ** |
| 3dCKR-3dCKL | miR6300 | 1387.0623 | 406.6345 | -1.77022806 | 0 | ** |
| 3dCKR-3dCKL | miR6421-3p | 512.1190 | 7253.7594 | 3.82417790 | 0 | ** |
| 3dCKR-3dCKL | miR6425a-5p | 8.1159 | 7.0529 | -0.20253450 | 0.276375606265719 |  |
| 3dCKR-3dCKL | miR6426a | 35.8609 | 28.6485 | -0.32395209 | 0.000331508128482397 |  |
| 3dCKR-3dCKL | miR6427-3p | 0.01 | 1.8725 | 7.54882191 | 1.05334962091032e-09 | ** |
| 3dCKR-3dCKL | miR6427-5p | 0.3146 | 2.8087 | 3.15831196 | 2.74198099945874e-09 | ** |
| 3dCKR-3dCKL | miR6428 | 113.6225 | 286.1109 | 1.33232591 | 9.56724475879537e-269 | ** |
| 3dCKR-3dCKL | miR6430 | 0.1887 | 9.9864 | 5.72579837 | 1.17802131903961e-43 | ** |
| 3dCKR-3dCKL | miR6433-3p | 5.6622 | 18.1004 | 1.67658697 | 1.79732036347609e-25 | ** |
| 3dCKR-3dCKL | miR6433-5p | 3.9007 | 13.3568 | 1.77576946 | 1.12085404029667e-20 | ** |
| 3dCKR-3dCKL | miR6438b | 2.3278 | 0.4993 | -2.22098830 | 8.18859540293821e-06 | ** |
| 3dCKR-3dCKL | miR6439a | 1.3841 | 2.6214 | 0.92138933 | 0.0136751074562067 |  |
| 3dCKR-3dCKL | miR6441 | 1863.8866 | 1102.6254 | -0.75737135 | 0 |  |
| 3dCKR-3dCKL | miR6445a | 14.2185 | 15.7910 | 0.15133326 | 0.252095228302567 |  |
| 3dCKR-3dCKL | miR6448 | 6.7947 | 54.0515 | 2.99185290 | 1.51317278626619e-146 | ** |
| 3dCKR-3dCKL | miR6450a | 293.3045 | 82.2631 | -1.83408186 | 0 | ** |
| 3dCKR-3dCKL | miR6453 | 53.0993 | 15.7910 | -1.74959031 | 5.61844426993803e-76 | ** |
| 3dCKR-3dCKL | miR6454 | 37.5596 | 0.3745 | -6.64807217 | 1.88820677008898e-169 | ** |
| 3dCKR-3dCKL | miR6457b | 1.0695 | 11.9213 | 3.47853318 | 2.13155230051909e-38 | ** |
| 3dCKR-3dCKL | miR6460 | 3.5232 | 8.1140 | 1.20352693 | 5.63302863433308e-08 | ** |
| 3dCKR-3dCKL | miR6462c-5p | 6.6060 | 0.4369 | -3.91840196 | 5.35543650796391e-24 | ** |
| 3dCKR-3dCKL | miR6466-5p | 2.0132 | 7.7395 | 1.94274986 | 5.01602664568439e-14 | ** |
| 3dCKR-3dCKL | miR6471 | 14.9735 | 68.2821 | 2.18909594 | 4.80574008385876e-130 | ** |
| 3dCKR-3dCKL | miR6474 | 23.3410 | 57.5467 | 1.30186683 | 1.13734797098946e-53 | ** |
| 3dCKR-3dCKL | miR6476 | 20.1324 | 2.0597 | -3.28901304 | 6.2184690339406e-61 | ** |
| 3dCKR-3dCKL | miR6478 | 224.6655 | 201.1015 | -0.15985476 | 5.0435363360933e-06 |  |
| 3dCKR-3dCKL | miR6485 | 9.5629 | 2.7463 | -1.79995896 | 1.67973510117624e-15 | ** |
| 3dCKR-3dCKL | miR774b-5p | 31.1424 | 45.0013 | 0.53108655 | 2.04019275247801e-10 |  |
| 3dCKR-3dCKL | miR780.2 | 6.8576 | 5.2429 | -0.38733873 | 0.0640198051795255 |  |
| 3dCKR-3dCKL | miR827 | 5.5364 | 5.1180 | -0.11336803 | 0.609515249337814 |  |
| 3dCKR-3dCKL | miR837-3p | 4.7185 | 36.1383 | 2.93712843 | 9.26534986215692e-97 | ** |
| 3dCKR-3dCKL | miR845b-5p | 204.5959 | 156.7868 | -0.38397313 | 8.19729001772454e-24 |  |
| 3dCKR-3dCKL | miR846-5p | 8.3046 | 32.8303 | 1.98304535 | 4.4951123178352e-56 | ** |
| 3dCKR-3dCKL | miR847-5p | 3.9636 | 0.0624 | -5.98912154 | 2.78267245792198e-18 | ** |
| 3dCKR-3dCKL | miR858 | 22.1457 | 38.9470 | 0.81448561 | 5.84529575984289e-18 |  |
| 3dCKR-3dCKL | miR860 | 8.3675 | 21.9701 | 1.39267289 | 1.15001182356903e-23 | ** |
| 3dCKR-3dCKL | miR860-3p | 25.9205 | 0.4993 | -5.69804283 | 1.71409292424452e-111 | ** |
