# Supplementary material for: Genome-wide analysis of salt-responsive and novel microRNAs in Populus euphratica by deep sequencing
Source: BMC Genet. 2014 Jun 20;15(Suppl 1):S6. doi: 10.1186/1471-2156-15-S1-S6 (PMC4118626; doi:10.1186/1471-2156-15-S1-S6)
Supplement: Additional file 8 — Significant expression changes in conserved miRNAs from salt-treated Populus euphratica in the leaf (3dSL) and root (3dSR) libraries. [file 1471-2156-15-S1-S6-S8.doc]

Additional file 8 - Significantly expression changed of conserved miRNAs identified in *P. euphratica* between treated leaf (3dSL) and treated root (3dSR) libraries.

| pairwise | miR-name | 3dSR-  expressed | 3dSL-  expressed | 3dSR-std | 3dSL-std | fold-change(log2 3dSL/3dSR) | p-value | sig-lable |
| --- | --- | --- | --- | --- | --- | --- | --- | --- |
| 3dSR-3dSL | miR4399 | 2228 | 0 | 131.0167 | 0.01 | -13.6775 | 0 | ** |
| 3dSR-3dSL | miR5221 | 503 | 0 | 29.5787 | 0.01 | -11.5303 | 1.7E-145 | ** |
| 3dSR-3dSL | miR5647 | 324 | 0 | 19.0527 | 0.01 | -10.8958 | 5.62E-94 | ** |
| 3dSR-3dSL | miR3626-5p | 205 | 0 | 12.0549 | 0.01 | -10.2354 | 1.01E-59 | ** |
| 3dSR-3dSL | miR2637 | 198 | 0 | 11.6433 | 0.01 | -10.1853 | 1.04E-57 | ** |
| 3dSR-3dSL | miR6454 | 1604 | 2 | 94.3226 | 0.1251 | -9.55838 | 0 | ** |
| 3dSR-3dSL | miR3954 | 5002 | 8 | 294.1407 | 0.5003 | -9.1995 | 0 | ** |
| 3dSR-3dSL | miR6022 | 813 | 2 | 47.8081 | 0.1251 | -8.57803 | 7.7E-230 | ** |
| 3dSR-3dSL | miR860-3p | 1679 | 7 | 98.7329 | 0.4378 | -7.81712 | 0 | ** |
| 3dSR-3dSL | miR5140 | 655 | 3 | 38.517 | 0.1876 | -7.68169 | 1.6E-182 | ** |
| 3dSR-3dSL | miR1886.2 | 400 | 2 | 23.5218 | 0.1251 | -7.55477 | 1.4E-111 | ** |
| 3dSR-3dSL | miR6032 | 172 | 1 | 10.1144 | 0.0625 | -7.33834 | 2.7E-48 | ** |
| 3dSR-3dSL | miR1535a | 911 | 6 | 53.571 | 0.3753 | -7.15726 | 6.5E-250 | ** |
| 3dSR-3dSL | miR2936 | 2288 | 19 | 134.545 | 1.1883 | -6.82305 | 0 | ** |
| 3dSR-3dSL | miR2666 | 654 | 6 | 38.4582 | 0.3753 | -6.6791 | 8.5E-177 | ** |
| 3dSR-3dSL | miR2089-3p | 1768 | 17 | 103.9666 | 1.0632 | -6.61156 | 0 | ** |
| 3dSR-3dSL | miR2630a | 99 | 1 | 5.8217 | 0.0625 | -6.54144 | 1.62E-27 | ** |
| 3dSR-3dSL | miR1508b | 616 | 7 | 36.2236 | 0.4378 | -6.37051 | 2.2E-164 | ** |
| 3dSR-3dSL | miR3514-5p | 61 | 1 | 3.5871 | 0.0625 | -5.84282 | 8.84E-17 | ** |
| 3dSR-3dSL | miR394b-3p | 1627 | 34 | 95.6751 | 2.1265 | -5.49159 | 0 | ** |
| 3dSR-3dSL | miR5234 | 135 | 3 | 7.9386 | 0.1876 | -5.40315 | 7.26E-35 | ** |
| 3dSR-3dSL | miR4413a | 35 | 1 | 2.0582 | 0.0625 | -5.04138 | 1.6E-09 | ** |
| 3dSR-3dSL | miR847-5p | 64 | 2 | 3.7635 | 0.1251 | -4.91092 | 2.09E-16 | ** |
| 3dSR-3dSL | miR5301 | 1241 | 43 | 72.9765 | 2.6894 | -4.76208 | 7.4E-291 | ** |
| 3dSR-3dSL | miR5672 | 50 | 2 | 2.9402 | 0.1251 | -4.55476 | 1.41E-12 | ** |
| 3dSR-3dSL | miR399f | 188 | 8 | 11.0553 | 0.5003 | -4.4658 | 1.22E-43 | ** |
| 3dSR-3dSL | miR5658 | 3049 | 153 | 179.2953 | 9.5692 | -4.2278 | 0 | ** |
| 3dSR-3dSL | miR2086-3p | 909 | 49 | 53.4534 | 3.0646 | -4.12451 | 5.6E-195 | ** |
| 3dSR-3dSL | miR5759 | 18 | 1 | 1.0585 | 0.0625 | -4.08202 | 6.93E-05 | ** |
| 3dSR-3dSL | miR2111a | 98 | 6 | 5.7629 | 0.3753 | -3.94068 | 1.42E-21 | ** |
| 3dSR-3dSL | miR5562-3p | 413 | 26 | 24.2863 | 1.6261 | -3.90065 | 5.99E-86 | ** |
| 3dSR-3dSL | miR319b-5p | 45 | 3 | 2.6462 | 0.1876 | -3.81819 | 2.58E-10 | ** |
| 3dSR-3dSL | miR415 | 28 | 2 | 1.6465 | 0.1251 | -3.71825 | 1.05E-06 | ** |
| 3dSR-3dSL | miR1515 | 40 | 3 | 2.3522 | 0.1876 | -3.64828 | 5.14E-09 | ** |
| 3dSR-3dSL | miR2604 | 2666 | 216 | 156.7731 | 13.5094 | -3.53664 | 0 | ** |
| 3dSR-3dSL | miR5037c | 1499 | 129 | 88.1481 | 8.0681 | -3.44963 | 2.3E-278 | ** |
| 3dSR-3dSL | miR5665 | 119 | 11 | 6.9977 | 0.688 | -3.3464 | 6.97E-23 | ** |
| 3dSR-3dSL | miR390a | 1240 | 120 | 72.9177 | 7.5052 | -3.28031 | 1.8E-220 | ** |
| 3dSR-3dSL | miR394a | 392 | 38 | 23.0514 | 2.3767 | -3.27782 | 8.49E-71 | ** |
| 3dSR-3dSL | miR6300 | 16006 | 1715 | 941.2266 | 107.2625 | -3.1334 | 0 | ** |
| 3dSR-3dSL | miR160a | 1005 | 119 | 59.0986 | 7.4427 | -2.98923 | 7.5E-164 | ** |
| 3dSR-3dSL | miR6485 | 168 | 20 | 9.8792 | 1.2509 | -2.98143 | 1.29E-28 | ** |
| 3dSR-3dSL | miR6476 | 460 | 57 | 27.0501 | 3.565 | -2.92366 | 2.81E-74 | ** |
| 3dSR-3dSL | miR5657 | 127 | 17 | 7.4682 | 1.0632 | -2.81235 | 8.73E-21 | ** |
| 3dSR-3dSL | miR6103-3p | 181 | 27 | 10.6436 | 1.6887 | -2.656 | 2.17E-27 | ** |
| 3dSR-3dSL | miR5740 | 20 | 3 | 1.1761 | 0.1876 | -2.64828 | 0.000482 | ** |
| 3dSR-3dSL | miR171e | 1270 | 192 | 74.6819 | 12.0084 | -2.63671 | 3.9E-181 | ** |
| 3dSR-3dSL | miR2119 | 180 | 31 | 10.5848 | 1.9389 | -2.44868 | 5.4E-25 | ** |
| 3dSR-3dSL | miR6478 | 2694 | 605 | 158.4196 | 37.8389 | -2.06581 | 2.2E-285 | ** |
| 3dSR-3dSL | miR159a | 8080 | 1896 | 475.1413 | 118.5829 | -2.00246 | 0 | ** |
| 3dSR-3dSL | miR845b-5p | 2554 | 608 | 150.187 | 38.0266 | -1.98168 | 1.8E-256 | ** |
| 3dSR-3dSL | miR5265 | 83598 | 21377 | 4915.948 | 1336.997 | -1.87847 | 0 | ** |
| 3dSR-3dSL | miR6441 | 41554 | 10672 | 2443.567 | 667.4665 | -1.87222 | 0 | ** |
| 3dSR-3dSL | miR5224b | 6326 | 1655 | 371.998 | 103.5098 | -1.84553 | 0 | ** |
| 3dSR-3dSL | miR3509-5p | 1012 | 267 | 59.5103 | 16.6992 | -1.83336 | 8.98E-93 | ** |
| 3dSR-3dSL | miR858 | 476 | 126 | 27.991 | 7.8805 | -1.8286 | 2.13E-44 | ** |
| 3dSR-3dSL | miR2916 | 19138 | 5115 | 1125.403 | 319.9111 | -1.8147 | 0 | ** |
| 3dSR-3dSL | miR4414b | 118 | 32 | 6.9389 | 2.0014 | -1.7937 | 7.78E-12 | ** |
| 3dSR-3dSL | miR166h-3p | 112090 | 31880 | 6591.409 | 1993.894 | -1.725 | 0 | ** |
| 3dSR-3dSL | miR948 | 21 | 6 | 1.2349 | 0.3753 | -1.71828 | 0.006127 | ** |
| 3dSR-3dSL | miR477a-5p | 2806 | 814 | 165.0057 | 50.9106 | -1.69648 | 4.1E-228 | ** |
| 3dSR-3dSL | miR396a | 9494 | 2818 | 558.291 | 176.2482 | -1.66341 | 0 | ** |
| 3dSR-3dSL | miR390d-3p | 19 | 6 | 1.1173 | 0.3753 | -1.5739 | 0.014534 | * |
| 3dSR-3dSL | miR1310 | 2013 | 651 | 118.3737 | 40.716 | -1.53968 | 4.5E-143 | ** |
| 3dSR-3dSL | miR4348 | 73 | 24 | 4.2927 | 1.501 | -1.51596 | 1.93E-06 | ** |
| 3dSR-3dSL | miR1520d | 93955 | 33557 | 5524.987 | 2098.779 | -1.39642 | 0 | ** |
| 3dSR-3dSL | miR6438b | 25 | 9 | 1.4701 | 0.5629 | -1.38496 | 0.010218 | * |
| 3dSR-3dSL | miR5671 | 7455 | 2741 | 438.3884 | 171.4323 | -1.35457 | 0 | ** |
| 3dSR-3dSL | miR156b-3p | 1922 | 723 | 113.0225 | 45.2191 | -1.32161 | 4.8E-109 | ** |
| 3dSR-3dSL | miR164a | 67109 | 26391 | 3946.319 | 1650.591 | -1.25752 | 0 | ** |
| 3dSR-3dSL | miR171b-3p | 25060 | 9869 | 1473.644 | 617.2439 | -1.25548 | 0 | ** |
| 3dSR-3dSL | miR2938 | 1044 | 433 | 61.392 | 27.0814 | -1.18075 | 1.2E-50 | ** |
| 3dSR-3dSL | miR156a | 374138 | 158751 | 22001.04 | 9928.877 | -1.14787 | 0 | ** |
| 3dSR-3dSL | miR6450a | 3506 | 1493 | 206.169 | 93.3778 | -1.14268 | 2.1E-157 | ** |
| 3dSR-3dSL | miR6145e | 64 | 28 | 3.7635 | 1.7512 | -1.10373 | 0.000492 | ** |
| 3dSR-3dSL | miR2913 | 95 | 42 | 5.5864 | 2.6268 | -1.08861 | 2.56E-05 | ** |
| 3dSR-3dSL | miR3434-3p | 294 | 135 | 17.2886 | 8.4434 | -1.03393 | 1.03E-12 | ** |
| 3dSR-3dSL | miR6462c-5p | 68 | 33 | 3.9987 | 2.0639 | -0.95416 | 0.001425 |  |
| 3dSR-3dSL | miR5646 | 7053 | 3512 | 414.7489 | 219.6535 | -0.91701 | 7.7E-220 |  |
| 3dSR-3dSL | miR473a-5p | 1776 | 939 | 104.437 | 58.7285 | -0.8305 | 2.82E-48 |  |
| 3dSR-3dSL | miR6173 | 459 | 264 | 26.9913 | 16.5115 | -0.70902 | 9.97E-11 |  |
| 3dSR-3dSL | miR780.2 | 78 | 45 | 4.5868 | 2.8145 | -0.70461 | 0.008345 |  |
| 3dSR-3dSL | miR1446a | 2992 | 1733 | 175.9434 | 108.3883 | -0.6989 | 7.11E-60 |  |
| 3dSR-3dSL | miR827 | 206 | 131 | 12.1137 | 8.1932 | -0.56414 | 0.000414 |  |
| 3dSR-3dSL | miR6445a | 481 | 668 | 28.285 | 41.7792 | 0.562748 | 5.04E-11 |  |
| 3dSR-3dSL | miR5020b | 946 | 1334 | 55.6292 | 83.4333 | 0.584781 | 7.07E-22 |  |
| 3dSR-3dSL | miR477a-3p | 371 | 536 | 21.8165 | 33.5234 | 0.619749 | 1.39E-10 |  |
| 3dSR-3dSL | miR5239 | 31 | 47 | 1.8229 | 2.9396 | 0.689384 | 0.037669 |  |
| 3dSR-3dSL | miR2912a | 10011 | 15430 | 588.693 | 965.0495 | 0.713087 | 0 |  |
| 3dSR-3dSL | miR475a-3p | 922 | 1435 | 54.2179 | 89.7502 | 0.727146 | 1.08E-33 |  |
| 3dSR-3dSL | miR846-5p | 273 | 438 | 16.0537 | 27.3941 | 0.770959 | 2.06E-12 |  |
| 3dSR-3dSL | miR5218 | 1119 | 1850 | 65.8024 | 115.7059 | 0.81425 | 7.08E-52 |  |
| 3dSR-3dSL | miR6428 | 2296 | 3920 | 135.0154 | 245.1714 | 0.860667 | 2.1E-118 |  |
| 3dSR-3dSL | miR2651 | 265 | 457 | 15.5832 | 28.5825 | 0.875141 | 1.17E-15 |  |
| 3dSR-3dSL | miR6474 | 892 | 1574 | 52.4537 | 98.4438 | 0.908256 | 3.98E-53 |  |
| 3dSR-3dSL | miR1447 | 333 | 592 | 19.5819 | 37.0259 | 0.919014 | 2.1E-21 |  |
| 3dSR-3dSL | miR6171 | 373 | 676 | 21.9341 | 42.2796 | 0.946786 | 2.24E-25 |  |
| 3dSR-3dSL | miR5725 | 13 | 24 | 0.7645 | 1.501 | 0.973336 | 0.047348 |  |
| 3dSR-3dSL | miR5772 | 1162 | 2182 | 68.331 | 136.4704 | 0.997976 | 4.38E-85 |  |
| 3dSR-3dSL | miR172a | 3167 | 6043 | 186.2342 | 377.9517 | 1.021084 | 4.9E-241 | ** |
| 3dSR-3dSL | miR3949 | 25 | 48 | 1.4701 | 3.0021 | 1.030058 | 0.003107 | ** |
| 3dSR-3dSL | miR5227 | 15 | 29 | 0.8821 | 1.8138 | 1.040001 | 0.02107 | * |
| 3dSR-3dSL | miR172a-3p | 3053 | 5944 | 179.5305 | 371.7598 | 1.050142 | 3.2E-248 | ** |
| 3dSR-3dSL | miR6457b | 15 | 30 | 0.8821 | 1.8763 | 1.088876 | 0.014842 | * |
| 3dSR-3dSL | miR5373 | 124 | 251 | 7.2918 | 15.6985 | 1.10628 | 5.78E-13 | ** |
| 3dSR-3dSL | miR1523a | 349 | 714 | 20.5228 | 44.6562 | 1.121633 | 9.08E-35 | ** |
| 3dSR-3dSL | miR479 | 843 | 1760 | 49.5723 | 110.0769 | 1.150906 | 1.41E-86 | ** |
| 3dSR-3dSL | miR167f-3p | 1730 | 3651 | 101.732 | 228.3471 | 1.166455 | 2.5E-181 | ** |
| 3dSR-3dSL | miR475a-5p | 1712 | 3716 | 100.6735 | 232.4124 | 1.207003 | 9.8E-195 | ** |
| 3dSR-3dSL | miR166a | 277560 | 631539 | 16321.81 | 39498.79 | 1.275008 | 0 | ** |
| 3dSR-3dSL | miR5021 | 256 | 612 | 15.054 | 38.2768 | 1.346323 | 1.66E-39 | ** |
| 3dSR-3dSL | miR168a-3p | 826 | 2044 | 48.5726 | 127.8394 | 1.396118 | 6E-135 | ** |
| 3dSR-3dSL | miR396b-3p | 7544 | 20406 | 443.622 | 1276.267 | 1.524527 | 0 | ** |
| 3dSR-3dSL | miR5298b | 25 | 68 | 1.4701 | 4.253 | 1.532567 | 1.52E-06 | ** |
| 3dSR-3dSL | miR5769 | 8 | 22 | 0.4704 | 1.376 | 1.548521 | 0.006463 | ** |
| 3dSR-3dSL | miR157a | 1682205 | 4717869 | 98921.41 | 295073.1 | 1.576717 | 0 | ** |
| 3dSR-3dSL | miR5230 | 7911 | 22276 | 465.2033 | 1393.224 | 1.582494 | 0 | ** |
| 3dSR-3dSL | miR167h | 124423 | 359284 | 7316.646 | 22470.96 | 1.618807 | 0 | ** |
| 3dSR-3dSL | miR165a | 836 | 2432 | 49.1607 | 152.1063 | 1.629503 | 5.4E-201 | ** |
| 3dSR-3dSL | miR6466-5p | 35 | 102 | 2.0582 | 6.3795 | 1.63206 | 6.49E-10 | ** |
| 3dSR-3dSL | miR6448 | 274 | 813 | 16.1125 | 50.848 | 1.658011 | 3.96E-70 | ** |
| 3dSR-3dSL | miR395a | 33 | 100 | 1.9406 | 6.2544 | 1.688369 | 3.68E-10 | ** |
| 3dSR-3dSL | miR5255 | 145 | 441 | 8.5267 | 27.5818 | 1.693657 | 5.7E-40 | ** |
| 3dSR-3dSL | miR169ac | 7448 | 23041 | 437.9767 | 1441.07 | 1.718214 | 0 | ** |
| 3dSR-3dSL | miR473a-3p | 355 | 1155 | 20.8756 | 72.238 | 1.79094 | 5E-110 | ** |
| 3dSR-3dSL | miR860 | 106 | 352 | 6.2333 | 22.0154 | 1.820445 | 1.74E-35 | ** |
| 3dSR-3dSL | miR165a-3p | 412 | 1480 | 24.2275 | 92.5647 | 1.933817 | 8.8E-156 | ** |
| 3dSR-3dSL | miR529 | 184 | 678 | 10.82 | 42.4046 | 1.97052 | 3.69E-74 | ** |
| 3dSR-3dSL | miR6433-3p | 108 | 430 | 6.3509 | 26.8938 | 2.082241 | 4.13E-51 | ** |
| 3dSR-3dSL | miR530b | 49 | 200 | 2.8814 | 12.5087 | 2.11809 | 3.93E-25 | ** |
| 3dSR-3dSL | miR774b-5p | 499 | 2209 | 29.3435 | 138.1591 | 2.235218 | 5.9E-279 | ** |
| 3dSR-3dSL | miR160b-3p | 479 | 2603 | 28.1674 | 162.8013 | 2.531014 | 0 | ** |
| 3dSR-3dSL | miR397b-3p | 8 | 49 | 0.4704 | 3.0646 | 2.703739 | 4.14E-09 | ** |
| 3dSR-3dSL | miR6453 | 255 | 1593 | 14.9952 | 99.6321 | 2.73211 | 1.1E-254 | ** |
| 3dSR-3dSL | miR157d-3p | 102 | 649 | 5.9981 | 40.5909 | 2.758579 | 6E-106 | ** |
| 3dSR-3dSL | miR837-3p | 62 | 420 | 3.6459 | 26.2684 | 2.848981 | 1.56E-71 | ** |
| 3dSR-3dSL | miR5248 | 17 | 122 | 0.9997 | 7.6303 | 2.932173 | 1.83E-22 | ** |
| 3dSR-3dSL | miR3629a-3p | 16 | 117 | 0.9409 | 7.3176 | 2.959257 | 8.64E-22 | ** |
| 3dSR-3dSL | miR6460 | 22 | 165 | 1.2937 | 10.3197 | 2.995826 | 1.67E-30 | ** |
| 3dSR-3dSL | miR397a | 105 | 823 | 6.1745 | 51.4735 | 3.059436 | 6.9E-149 | ** |
| 3dSR-3dSL | miR6471 | 188 | 1534 | 11.0553 | 95.9421 | 3.117426 | 4.8E-281 | ** |
| 3dSR-3dSL | miR1444a | 17 | 143 | 0.9997 | 8.9438 | 3.161321 | 5.46E-28 | ** |
| 3dSR-3dSL | miR6421-3p | 11182 | 102356 | 657.5532 | 6401.724 | 3.283281 | 0 | ** |
| 3dSR-3dSL | miR408b | 970 | 10253 | 57.0405 | 641.2607 | 3.490852 | 0 | ** |
| 3dSR-3dSL | miR6427-5p | 7 | 92 | 0.4116 | 5.754 | 3.80525 | 1.83E-21 | ** |
| 3dSR-3dSL | miR6433-5p | 86 | 1165 | 5.0572 | 72.8634 | 3.848784 | 2.8E-257 | ** |
| 3dSR-3dSL | miR393h | 112 | 1882 | 6.5861 | 117.7073 | 4.159636 | 0 | ** |
| 3dSR-3dSL | miR6447 | 1 | 18 | 0.0588 | 1.1258 | 4.258991 | 2.27E-05 | ** |
| 3dSR-3dSL | miR6421-5p | 5 | 99 | 0.294 | 6.1918 | 4.396471 | 2.54E-25 | ** |
| 3dSR-3dSL | miR6430 | 5 | 142 | 0.294 | 8.8812 | 4.916867 | 4.24E-38 | ** |
| 3dSR-3dSL | miR6424 | 1 | 43 | 0.0588 | 2.6894 | 5.515324 | 6.79E-13 | ** |
| 3dSR-3dSL | miR398c-5p | 7 | 351 | 0.4116 | 21.9528 | 5.737018 | 5.1E-99 | ** |
| 3dSR-3dSL | miR6427-3p | 2 | 225 | 0.1176 | 14.0723 | 6.902826 | 1.09E-67 | ** |
| 3dSR-3dSL | miR3627-5p | 47 | 11819 | 2.7638 | 739.2042 | 8.063176 | 0 | ** |
| 3dSR-3dSL | miR391 | 39 | 10818 | 2.2934 | 676.5979 | 8.204667 | 0 | ** |
