# Supplementary material for: Genome-wide analysis of salt-responsive and novel microRNAs in Populus euphratica by deep sequencing
Source: BMC Genet. 2014 Jun 20;15(Suppl 1):S6. doi: 10.1186/1471-2156-15-S1-S6 (PMC4118626; doi:10.1186/1471-2156-15-S1-S6)
Supplement: Additional file 9 — Significant expression changes in novel miRNAs in the leaves of salt-treated Populus euphratica (3dSL) and control-treated (3dCKL) libraries. [file 1471-2156-15-S1-S6-S9.doc]

Additional file 9 - Significantly expression changed of novel miRNAs identified in *P. euphratica* between treated (3dSL) with salt and control (3dCKL) libraries in leaf tissue.

| pairwise | miR-name | 3dCKL-std | 3dSL-std | fold-change(log2 3dSL/3dCKL) | p-value | sig-lable |
| --- | --- | --- | --- | --- | --- | --- |
| 3dCKL-3dSL | novel_mir_106 | 8.1140 | 8.8187 | 0.12015270 | 0.494072964326719 |  |
| 3dCKL-3dSL | novel_mir_11 | 4.9308 | 4.1904 | -0.23473377 | 0.328452963890019 |  |
| 3dCKL-3dSL | novel_mir_111 | 9.3623 | 10.7575 | 0.20040794 | 0.213951582319861 |  |
| 3dCKL-3dSL | novel_mir_119 | 0.01 | 8.5059 | 9.73232008 | 9.96909546585493e-42 | ** |
| 3dCKL-3dSL | novel_mir_122 | 1.6228 | 5.7540 | 1.82608001 | 4.60485346164009e-10 | ** |
| 3dCKL-3dSL | novel_mir_124 | 14.4803 | 0.01 | -10.49987583 | 1.84131786089361e-70 | ** |
| 3dCKL-3dSL | novel_mir_13 | 0.9362 | 4.5031 | 2.26602985 | 2.40986971727572e-10 | ** |
| 3dCKL-3dSL | novel_mir_131 | 2.9335 | 9.8194 | 1.74301188 | 2.77798783422464e-15 | ** |
| 3dCKL-3dSL | novel_mir_135 | 0.01 | 5.2537 | 9.03719001 | 4.73662862607899e-26 | ** |
| 3dCKL-3dSL | novel_mir_143 | 0.6866 | 1.0632 | 0.63087125 | 0.262550686918725 |  |
| 3dCKL-3dSL | novel_mir_146 | 4.1818 | 0.01 | -8.70798028 | 7.26721207542144e-21 | ** |
| 3dCKL-3dSL | novel_mir_150 | 11.8589 | 0.01 | -10.21175453 | 7.7557889620689e-58 | ** |
| 3dCKL-3dSL | novel_mir_152 | 0.01 | 20.1391 | 10.97578350 | 8.39265723479069e-98 | ** |
| 3dCKL-3dSL | novel_mir_158 | 0.3745 | 1.3760 | 1.87744285 | 0.00227415591480683 | ** |
| 3dCKL-3dSL | novel_mir_166 | 1.4980 | 0.01 | -7.22689382 | 6.11573020999125e-08 | ** |
| 3dCKL-3dSL | novel_mir_17 | 1.8100 | 4.0028 | 1.14501984 | 0.000251790689306051 | ** |
| 3dCKL-3dSL | novel_mir_171 | 9.2374 | 0.01 | -9.85134303 | 3.26680491737236e-45 | ** |
| 3dCKL-3dSL | novel_mir_178 | 0.8114 | 1.3134 | 0.69482115 | 0.173527568478112 |  |
| 3dCKL-3dSL | novel_mir_182 | 14.1682 | 77.5542 | 2.45254843 | 1.11479743909562e-169 | ** |
| 3dCKL-3dSL | novel_mir_187 | 0.01 | 2.3767 | 7.89281600 | 3.49477974797114e-12 | ** |
| 3dCKL-3dSL | novel_mir_188 | 0.01 | 1.2509 | 6.96682265 | 9.33273913221773e-07 | ** |
| 3dCKL-3dSL | novel_mir_19 | 2.6838 | 3.3774 | 0.33163589 | 0.262040299053078 |  |
| 3dCKL-3dSL | novel_mir_2 | 1.4980 | 0.01 | -7.22689382 | 6.11573020999125e-08 | ** |
| 3dCKL-3dSL | novel_mir_23 | 2.3094 | 6.6296 | 1.52140375 | 4.13092196088814e-09 | ** |
| 3dCKL-3dSL | novel_mir_233 | 0.5617 | 1.0632 | 0.92054130 | 0.120789676636472 |  |
| 3dCKL-3dSL | novel_mir_237 | 0.01 | 8.9438 | 9.80474412 | 7.73242052986757e-44 | ** |
| 3dCKL-3dSL | novel_mir_238 | 0.01 | 119.7712 | 13.54799342 | 0 | ** |
| 3dCKL-3dSL | novel_mir_239 | 0.01 | 20.3267 | 10.98916030 | 1.04584646713857e-98 | ** |
| 3dCKL-3dSL | novel_mir_244 | 2.6214 | 3.3148 | 0.33858432 | 0.257204248241311 |  |
| 3dCKL-3dSL | novel_mir_28 | 1.1235 | 0.6880 | -0.70751965 | 0.202485244020531 |  |
| 3dCKL-3dSL | novel_mir_283 | 1084.4001 | 0.01 | -16.72653584 | 0 | ** |
| 3dCKL-3dSL | novel_mir_295 | 0.01 | 4.0653 | 8.66721801 | 2.53242058976064e-20 | ** |
| 3dCKL-3dSL | novel_mir_310 | 1.1235 | 0.01 | -6.81185631 | 3.88998495043461e-06 | ** |
| 3dCKL-3dSL | novel_mir_315 | 2.3094 | 0.01 | -7.85137428 | 7.56599145729459e-12 | ** |
| 3dCKL-3dSL | novel_mir_316 | 0.01 | 2.4392 | 7.93026425 | 1.74559153057513e-12 | ** |
| 3dCKL-3dSL | novel_mir_32 | 306.8951 | 64.1073 | -2.25918506 | 0 | ** |
| 3dCKL-3dSL | novel_mir_356 | 1.7476 | 0.01 | -7.44923120 | 3.83809084807944e-09 | ** |
| 3dCKL-3dSL | novel_mir_362 | 2.1221 | 0.01 | -7.72934882 | 6.03414369139416e-11 | ** |
| 3dCKL-3dSL | novel_mir_407 | 1.2483 | 0.01 | -6.96382089 | 9.74498975711124e-07 | ** |
| 3dCKL-3dSL | novel_mir_412 | 0.01 | 1.0632 | 6.73226920 | 7.48929054689076e-06 | ** |
| 3dCKL-3dSL | novel_mir_433 | 1.8725 | 0.01 | -7.54882192 | 9.61498732719071e-10 | ** |
| 3dCKL-3dSL | novel_mir_435 | 1.3731 | 0.01 | -7.10129288 | 2.44126459552466e-07 | ** |
| 3dCKL-3dSL | novel_mir_44 | 15.6038 | 61.3554 | 1.97529291 | 3.77102355952574e-103 | ** |
| 3dCKL-3dSL | novel_mir_467 | 0.01 | 1.2509 | 6.96682265 | 9.33273913221773e-07 | ** |
| 3dCKL-3dSL | novel_mir_468 | 0.01 | 1.0632 | 6.73226920 | 7.48929054689076e-06 | ** |
| 3dCKL-3dSL | novel_mir_47 | 0.01 | 1.4385 | 7.16842141 | 1.162994267143e-07 | ** |
| 3dCKL-3dSL | novel_mir_48 | 0.3745 | 1.0007 | 1.41797191 | 0.0342707452328591 | * |
| 3dCKL-3dSL | novel_mir_49 | 646.9950 | 580.7809 | -0.15576055 | 3.98315598018816e-14 |  |
| 3dCKL-3dSL | novel_mir_51 | 860.1427 | 2761.0548 | 1.68257159 | 0 | ** |
| 3dCKL-3dSL | novel_mir_52 | 3.7449 | 8.3809 | 1.16217798 | 7.5217757900713e-08 | ** |
| 3dCKL-3dSL | novel_mir_53 | 48.3093 | 58.9162 | 0.28636343 | 4.1307360704851e-05 |  |
| 3dCKL-3dSL | novel_mir_55 | 2.4342 | 49.2219 | 4.33778073 | 1.30021151068187e-182 | ** |
| 3dCKL-3dSL | novel_mir_56 | 1.1235 | 0.7505 | -0.58207615 | 0.283619414609193 |  |
| 3dCKL-3dSL | novel_mir_59 | 0.01 | 132.5927 | 13.69471373 | 0 | ** |
| 3dCKL-3dSL | novel_mir_6 | 0.5617 | 3.8152 | 2.76388698 | 6.94339310440762e-11 | ** |
| 3dCKL-3dSL | novel_mir_62 | 5.9294 | 15.6359 | 1.39890423 | 1.8941518323431e-17 | ** |
| 3dCKL-3dSL | novel_mir_65 | 0.6242 | 1.3134 | 1.07322610 | 0.0494261285251229 | * |
| 3dCKL-3dSL | novel_mir_7 | 3.4952 | 2.0639 | -0.76000194 | 0.0152716437021443 |  |
| 3dCKL-3dSL | novel_mir_70 | 2.3094 | 2.3767 | 0.04144173 | 0.901645572791932 |  |
| 3dCKL-3dSL | novel_mir_75 | 4.3691 | 31.8973 | 2.86802628 | 6.32404829207861e-84 | ** |
| 3dCKL-3dSL | novel_mir_8 | 33.3921 | 278.7573 | 3.06143086 | 0 | ** |
| 3dCKL-3dSL | novel_mir_81 | 0.5617 | 6.5045 | 3.53356645 | 6.17146216647035e-22 | ** |
| 3dCKL-3dSL | novel_mir_85 | 0.5617 | 3.1272 | 2.47699978 | 2.95323570170294e-08 | ** |
| 3dCKL-3dSL | novel_mir_88 | 13.1072 | 29.6457 | 1.17746334 | 1.25526025627944e-24 | ** |
| 3dCKL-3dSL | novel_mir_89 | 0.01 | 7.1925 | 9.49034951 | 2.13637075346054e-35 | ** |
| 3dCKL-3dSL | novel_mir_90 | 0.01 | 2.4392 | 7.93026425 | 1.74559153057513e-12 | ** |
| 3dCKL-3dSL | novel_mir_94 | 1.1235 | 0.01 | -6.81185631 | 3.88998495043461e-06 | ** |
| 3dCKL-3dSL | novel_mir_96 | 22.5319 | 16.5115 | -0.44849778 | 0.000114086718067311 |  |
| 3dCKL-3dSL | novel_mir_99 | 148.7976 | 306.0264 | 1.04030486 | 3.78943237161673e-195 | ** |
