# Supplementary material for: Genome-wide analysis of salt-responsive and novel microRNAs in Populus euphratica by deep sequencing
Source: BMC Genet. 2014 Jun 20;15(Suppl 1):S6. doi: 10.1186/1471-2156-15-S1-S6 (PMC4118626; doi:10.1186/1471-2156-15-S1-S6)
Supplement: Additional file 10 — Significant expression changes in novel miRNAs in the roots of salt-treated Populus euphratica (3dSR) and control-treated (3dCKR) libraries. [file 1471-2156-15-S1-S6-S10.doc]

Additional file 10 - Significantly expression changed of novel miRNAs identified in *P. euphratica* between treated root (3dSR) and control root (3dCKR) libraries.

| pairwise | miR-name | 3dCKR-std | 3dSR-std | fold-change(log2 3dSR/3dCKR) | p-value | sig-lable |
| --- | --- | --- | --- | --- | --- | --- |
| 3dCKR-3dSR | novel_mir_100 | 2.2020 | 3.9987 | 0.86071658 | 0.00350637717599609 |  |
| 3dCKR-3dSR | novel_mir_102 | 1.1324 | 0.6469 | -0.80776904 | 0.142660507510893 |  |
| 3dCKR-3dSR | novel_mir_106 | 1.3212 | 1.7053 | 0.36817669 | 0.379457058706293 |  |
| 3dCKR-3dSR | novel_mir_109 | 0.01 | 1.0585 | 6.72587746 | 7.16866323068315e-06 | ** |
| 3dCKR-3dSR | novel_mir_11 | 6.4801 | 7.6446 | 0.23842494 | 0.211304655034388 |  |
| 3dCKR-3dSR | novel_mir_111 | 4.1523 | 4.2927 | 0.04797467 | 0.848347133518728 |  |
| 3dCKR-3dSR | novel_mir_113 | 1.5099 | 0.01 | -7.23830919 | 2.52581009288414e-08 | ** |
| 3dCKR-3dSR | novel_mir_114 | 3.3973 | 3.6459 | 0.10188637 | 0.708771159415943 |  |
| 3dCKR-3dSR | novel_mir_115 | 1.3841 | 0.01 | -7.11280437 | 1.08215890712171e-07 | ** |
| 3dCKR-3dSR | novel_mir_119 | 1.0066 | 4.0575 | 2.01110059 | 1.92842692893915e-08 | ** |
| 3dCKR-3dSR | novel_mir_122 | 10.8841 | 7.7622 | -0.48768461 | 0.00331134377415407 |  |
| 3dCKR-3dSR | novel_mir_124 | 0.01 | 1.5289 | 7.25635024 | 3.65222406274319e-08 | ** |
| 3dCKR-3dSR | novel_mir_127 | 1.5728 | 2.3522 | 0.58067551 | 0.114557672817737 |  |
| 3dCKR-3dSR | novel_mir_128 | 34.2252 | 38.6346 | 0.17483449 | 0.0364722536363624 |  |
| 3dCKR-3dSR | novel_mir_13 | 2.3278 | 0.4116 | -2.49965222 | 1.05387292448864e-06 | ** |
| 3dCKR-3dSR | novel_mir_131 | 1.5728 | 0.01 | -7.29719141 | 1.2202688419984e-08 | ** |
| 3dCKR-3dSR | novel_mir_134 | 0.01 | 23.5806 | 11.20338471 | 1.21275074515893e-115 | ** |
| 3dCKR-3dSR | novel_mir_135 | 1.3841 | 0.01 | -7.11280437 | 1.08215890712171e-07 | ** |
| 3dCKR-3dSR | novel_mir_136 | 7.1722 | 3.7047 | -0.95305900 | 1.73809343568829e-05 |  |
| 3dCKR-3dSR | novel_mir_152 | 2.3278 | 0.01 | -7.86282330 | 1.97293809059453e-12 | ** |
| 3dCKR-3dSR | novel_mir_182 | 0.01 | 20.8168 | 11.02353260 | 3.58464411348957e-102 | ** |
| 3dCKR-3dSR | novel_mir_19 | 1.5099 | 0.4116 | -1.87513812 | 0.00110098039432999 | ** |
| 3dCKR-3dSR | novel_mir_192 | 0.8808 | 1.1173 | 0.34313023 | 0.509948377788175 |  |
| 3dCKR-3dSR | novel_mir_204 | 7.2980 | 2.5874 | -1.49599805 | 5.8073576018792e-10 | ** |
| 3dCKR-3dSR | novel_mir_212 | 1.1324 | 0.8233 | -0.45989352 | 0.373129636097308 |  |
| 3dCKR-3dSR | novel_mir_218 | 5.8510 | 0.01 | -9.19253937 | 4.00114069064616e-30 | ** |
| 3dCKR-3dSR | novel_mir_226 | 6.6060 | 0.01 | -9.36763312 | 6.46906862054672e-34 | ** |
| 3dCKR-3dSR | novel_mir_23 | 12.7086 | 14.8188 | 0.22162352 | 0.103909770278722 |  |
| 3dCKR-3dSR | novel_mir_244 | 1.1324 | 1.5877 | 0.48755468 | 0.271620966374725 |  |
| 3dCKR-3dSR | novel_mir_255 | 0.01 | 1.2937 | 7.01535929 | 5.11679238867536e-07 | ** |
| 3dCKR-3dSR | novel_mir_30 | 0.01 | 1.7641 | 7.46278853 | 2.60685593458915e-09 | ** |
| 3dCKR-3dSR | novel_mir_32 | 276.3178 | 266.3261 | -0.05313468 | 0.081988475198497 |  |
| 3dCKR-3dSR | novel_mir_332 | 5.3477 | 0.5880 | -3.18503047 | 2.3154679075074e-17 | ** |
| 3dCKR-3dSR | novel_mir_375 | 1.6358 | 0.01 | -7.35385255 | 5.89536026856158e-09 | ** |
| 3dCKR-3dSR | novel_mir_42 | 10.0033 | 6.8213 | -0.55235739 | 0.00161363172397181 |  |
| 3dCKR-3dSR | novel_mir_433 | 0.01 | 2.1170 | 7.72587746 | 4.97114529964666e-11 | ** |
| 3dCKR-3dSR | novel_mir_44 | 1.5728 | 0.6469 | -1.28172061 | 0.0114235981753798 | * |
| 3dCKR-3dSR | novel_mir_47 | 3.2086 | 1.7641 | -0.86301160 | 0.00841698274353095 |  |
| 3dCKR-3dSR | novel_mir_485 | 1.1324 | 0.01 | -6.82323985 | 1.98642296130354e-06 | ** |
| 3dCKR-3dSR | novel_mir_488 | 2.6424 | 0.01 | -8.04570506 | 5.19263262117002e-14 | ** |
| 3dCKR-3dSR | novel_mir_49 | 79.0827 | 53.5710 | -0.56190990 | 1.96559358713644e-19 |  |
| 3dCKR-3dSR | novel_mir_51 | 321.0495 | 379.7014 | 0.24206957 | 2.6735125860555e-19 |  |
| 3dCKR-3dSR | novel_mir_52 | 2.7053 | 0.5292 | -2.35390362 | 3.57783227585806e-07 | ** |
| 3dCKR-3dSR | novel_mir_520 | 0.01 | 1.1173 | 6.80387280 | 3.70534015511791e-06 | ** |
| 3dCKR-3dSR | novel_mir_521 | 0.01 | 1.2937 | 7.01535929 | 5.11679238867536e-07 | ** |
| 3dCKR-3dSR | novel_mir_524 | 0.01 | 1.1761 | 6.87786692 | 1.91521699699377e-06 | ** |
| 3dCKR-3dSR | novel_mir_53 | 23.5927 | 28.0498 | 0.24964995 | 0.0120327352439524 |  |
| 3dCKR-3dSR | novel_mir_55 | 22.8377 | 54.1590 | 1.24578374 | 1.5853994536725e-48 | ** |
| 3dCKR-3dSR | novel_mir_59 | 882.7446 | 602.5709 | -0.55086509 | 2.13379631543411e-192 |  |
| 3dCKR-3dSR | novel_mir_6 | 3.9007 | 2.7050 | -0.52810445 | 0.0591613720862994 |  |
| 3dCKR-3dSR | novel_mir_62 | 3.3973 | 3.9987 | 0.23514243 | 0.374501365075974 |  |
| 3dCKR-3dSR | novel_mir_63 | 3.1457 | 3.5871 | 0.18943687 | 0.495457695915774 |  |
| 3dCKR-3dSR | novel_mir_65 | 0.4404 | 1.1173 | 1.34313023 | 0.0301140219555015 | * |
| 3dCKR-3dSR | novel_mir_8 | 37.8112 | 20.4052 | -0.88987679 | 1.0584038348002e-20 |  |
| 3dCKR-3dSR | novel_mir_81 | 1.1324 | 0.7057 | -0.68225674 | 0.205139306062997 |  |
| 3dCKR-3dSR | novel_mir_84 | 1.8245 | 2.8814 | 0.65926880 | 0.049907413571378 |  |
| 3dCKR-3dSR | novel_mir_88 | 87.8278 | 22.6986 | -1.95207435 | 2.11278311258632e-149 | ** |
| 3dCKR-3dSR | novel_mir_89 | 0.01 | 4.5280 | 8.82273015 | 8.81947099099059e-23 | ** |
| 3dCKR-3dSR | novel_mir_95 | 0.01 | 1.8229 | 7.51009161 | 1.34743224255484e-09 | ** |
| 3dCKR-3dSR | novel_mir_96 | 2.2020 | 1.9406 | -0.18231169 | 0.601650627503808 |  |
| 3dCKR-3dSR | novel_mir_98 | 12.0795 | 0.01 | -10.23834508 | 2.10674411404832e-61 | ** |
| 3dCKR-3dSR | novel_mir_99 | 0.01 | 29.5199 | 11.52747212 | 1.36820726123967e-144 | ** |
