# Supplementary material for: Genome-wide analysis of salt-responsive and novel microRNAs in Populus euphratica by deep sequencing
Source: BMC Genet. 2014 Jun 20;15(Suppl 1):S6. doi: 10.1186/1471-2156-15-S1-S6 (PMC4118626; doi:10.1186/1471-2156-15-S1-S6)
Supplement: Additional file 11 — Significant expression changes in novel miRNAs in control-treated Populus euphratica leaf (3dCKL) and root (3dCKR) libraries. [file 1471-2156-15-S1-S6-S11.doc]

Additional file 11 - Significantly expression changed of novel miRNAs identified in *P. euphratica* between control leaf (3dCKL) and control root (3dCKR) libraries.

| pairwise | miR-name | 3wkCKR-std | 3wkCKL-std | fold-change(log2 3wkCKL/3wkCKR) | p-value | sig-lable |
| --- | --- | --- | --- | --- | --- | --- |
| 3wkCKR-3wkCKL | novel_mir_100 | 4.3541 | 0.01 | -8.76623072 | 6.2631827618617e-20 | ** |
| 3wkCKR-3wkCKL | novel_mir_102 | 1.2265 | 0.01 | -6.93840342 | 4.09538573584547e-06 | ** |
| 3wkCKR-3wkCKL | novel_mir_106 | 1.2878 | 13.0310 | 3.33896734 | 2.50830734652441e-39 | ** |
| 3wkCKR-3wkCKL | novel_mir_11 | 11.1611 | 7.3654 | -0.59964344 | 0.000622558129325082 |  |
| 3wkCKR-3wkCKL | novel_mir_111 | 3.0662 | 10.8356 | 1.82125533 | 5.01747391791359e-17 | ** |
| 3wkCKR-3wkCKL | novel_mir_113 | 3.6795 | 0.01 | -8.52336591 | 5.97872739278578e-17 | ** |
| 3wkCKR-3wkCKL | novel_mir_114 | 4.8447 | 0.01 | -8.92026352 | 4.26243601237366e-22 | ** |
| 3wkCKR-3wkCKL | novel_mir_119 | 0.01 | 3.2578 | 8.34775423 | 4.27037644862108e-16 | ** |
| 3wkCKR-3wkCKL | novel_mir_122 | 9.3214 | 3.9660 | -1.23286197 | 9.17702830994929e-09 | ** |
| 3wkCKR-3wkCKL | novel_mir_124 | 1.0425 | 16.9262 | 4.02113883 | 7.69328294151434e-59 | ** |
| 3wkCKR-3wkCKL | novel_mir_127 | 5.0286 | 0.9207 | -2.44935375 | 1.48713594169382e-11 | ** |
| 3wkCKR-3wkCKL | novel_mir_13 | 1.1652 | 52.4075 | 5.49112378 | 4.51031884591922e-215 | ** |
| 3wkCKR-3wkCKL | novel_mir_131 | 1.3491 | 8.1444 | 2.59381113 | 1.00010840796915e-19 | ** |
| 3wkCKR-3wkCKL | novel_mir_135 | 1.1652 | 1.2748 | 0.12969332 | 0.776014147079846 |  |
| 3wkCKR-3wkCKL | novel_mir_136 | 2.9436 | 0.01 | -8.20143783 | 1.06491408241902e-13 | ** |
| 3wkCKR-3wkCKL | novel_mir_150 | 0.01 | 4.6742 | 8.86857566 | 9.16518267290814e-23 | ** |
| 3wkCKR-3wkCKL | novel_mir_152 | 1.5944 | 5.6657 | 1.82924060 | 1.28063609054764e-09 | ** |
| 3wkCKR-3wkCKL | novel_mir_153 | 1.5331 | 0.01 | -7.26030800 | 1.81064810983946e-07 | ** |
| 3wkCKR-3wkCKL | novel_mir_158 | 0.3679 | 2.1954 | 2.57709824 | 3.3045782712833e-06 | ** |
| 3wkCKR-3wkCKL | novel_mir_17 | 0.3679 | 2.6912 | 2.87086403 | 4.38600721021441e-08 | ** |
| 3wkCKR-3wkCKL | novel_mir_171 | 0.01 | 5.5240 | 9.10956951 | 9.14420816010918e-27 | ** |
| 3wkCKR-3wkCKL | novel_mir_18 | 1.6558 | 2.7620 | 0.73818490 | 0.0395582947576278 |  |
| 3wkCKR-3wkCKL | novel_mir_182 | 0.01 | 24.6457 | 11.26712024 | 8.6847659542594e-117 | ** |
| 3wkCKR-3wkCKL | novel_mir_188 | 0.6132 | 1.9122 | 1.64080382 | 0.00119228424626794 | ** |
| 3wkCKR-3wkCKL | novel_mir_19 | 0.6132 | 3.4702 | 2.50058921 | 7.54121821344282e-09 | ** |
| 3wkCKR-3wkCKL | novel_mir_197 | 0.01 | 22.3794 | 11.12795564 | 4.05581536897112e-106 | ** |
| 3wkCKR-3wkCKL | novel_mir_212 | 1.1038 | 0.5666 | -0.96207628 | 0.116643361098526 |  |
| 3wkCKR-3wkCKL | novel_mir_221 | 0.01 | 1.3456 | 7.07210580 | 4.29244718033473e-07 | ** |
| 3wkCKR-3wkCKL | novel_mir_23 | 10.1799 | 15.0849 | 0.56738174 | 0.000135418506278479 |  |
| 3wkCKR-3wkCKL | novel_mir_240 | 0.01 | 21.6004 | 11.07684231 | 1.88650059421988e-102 | ** |
| 3wkCKR-3wkCKL | novel_mir_255 | 1.8397 | 0.01 | -7.52332670 | 8.0052204825778e-09 | ** |
| 3wkCKR-3wkCKL | novel_mir_256 | 3.8635 | 0.01 | -8.59376461 | 9.20306092445744e-18 | ** |
| 3wkCKR-3wkCKL | novel_mir_295 | 42.3756 | 0.01 | -12.04901783 | 6.97687801340149e-188 | ** |
| 3wkCKR-3wkCKL | novel_mir_30 | 1.0425 | 0.01 | -6.70390358 | 2.66054903731508e-05 | ** |
| 3wkCKR-3wkCKL | novel_mir_315 | 1.3491 | 0.01 | -7.07585347 | 1.17627945113594e-06 | ** |
| 3wkCKR-3wkCKL | novel_mir_32 | 218.2556 | 225.7062 | 0.04842738 | 0.168508420044345 |  |
| 3wkCKR-3wkCKL | novel_mir_321 | 20.1759 | 0.01 | -10.97841743 | 8.06528999208598e-90 | ** |
| 3wkCKR-3wkCKL | novel_mir_331 | 0.01 | 21.5296 | 11.07210580 | 4.06511838896652e-102 | ** |
| 3wkCKR-3wkCKL | novel_mir_382 | 0.01 | 1.1331 | 6.82413138 | 4.2949065143918e-06 | ** |
| 3wkCKR-3wkCKL | novel_mir_42 | 14.1661 | 0.4957 | -4.83683156 | 1.10467751641108e-52 | ** |
| 3wkCKR-3wkCKL | novel_mir_423 | 0.01 | 2.1246 | 7.73104744 | 9.22839531450307e-11 | ** |
| 3wkCKR-3wkCKL | novel_mir_435 | 0.01 | 1.2748 | 6.99412711 | 9.24956293144874e-07 | ** |
| 3wkCKR-3wkCKL | novel_mir_44 | 4.0474 | 44.5463 | 3.46023827 | 8.03290299013376e-135 | ** |
| 3wkCKR-3wkCKL | novel_mir_47 | 2.0850 | 2.1954 | 0.07443644 | 0.827706637711785 |  |
| 3wkCKR-3wkCKL | novel_mir_48 | 0.01 | 1.1331 | 6.82413138 | 4.2949065143918e-06 | ** |
| 3wkCKR-3wkCKL | novel_mir_49 | 79.7838 | 410.2655 | 2.36239010 | 0 | ** |
| 3wkCKR-3wkCKL | novel_mir_498 | 0.01 | 1.3456 | 7.07210580 | 4.29244718033473e-07 | ** |
| 3wkCKR-3wkCKL | novel_mir_504 | 0.01 | 144.6163 | 13.81994255 | 0 | ** |
| 3wkCKR-3wkCKL | novel_mir_51 | 609.8155 | 1366.9141 | 1.16447786 | 0 | ** |
| 3wkCKR-3wkCKL | novel_mir_517 | 6.0098 | 0.01 | -9.23117316 | 3.03883411401331e-27 | ** |
| 3wkCKR-3wkCKL | novel_mir_518 | 1.3491 | 0.01 | -7.07585347 | 1.17627945113594e-06 | ** |
| 3wkCKR-3wkCKL | novel_mir_52 | 4.1088 | 65.5093 | 3.99491072 | 4.78807847438209e-222 | ** |
| 3wkCKR-3wkCKL | novel_mir_53 | 18.9494 | 84.1352 | 2.15055734 | 6.42938884988392e-151 | ** |
| 3wkCKR-3wkCKL | novel_mir_55 | 19.3174 | 14.3058 | -0.43330075 | 0.000795109359946338 |  |
| 3wkCKR-3wkCKL | novel_mir_56 | 1.1038 | 25.4955 | 4.52969194 | 3.24973180339997e-95 | ** |
| 3wkCKR-3wkCKL | novel_mir_58 | 1.5331 | 0.01 | -7.26030800 | 1.81064810983946e-07 | ** |
| 3wkCKR-3wkCKL | novel_mir_59 | 227.6383 | 162.8172 | -0.48349020 | 2.61549901896078e-37 |  |
| 3wkCKR-3wkCKL | novel_mir_6 | 8.9534 | 1.4164 | -2.66020689 | 2.74214702683984e-21 | ** |
| 3wkCKR-3wkCKL | novel_mir_62 | 3.6795 | 7.3654 | 1.00125414 | 1.26532591181974e-05 | ** |
| 3wkCKR-3wkCKL | novel_mir_63 | 0.01 | 1.8413 | 7.52458089 | 1.98971648070267e-09 | ** |
| 3wkCKR-3wkCKL | novel_mir_65 | 2.5756 | 2.2663 | -0.18456971 | 0.595196259406727 |  |
| 3wkCKR-3wkCKL | novel_mir_7 | 0.01 | 4.6742 | 8.86857566 | 9.16518267290814e-23 | ** |
| 3wkCKR-3wkCKL | novel_mir_70 | 0.01 | 1.9122 | 7.57908961 | 9.23368267295976e-10 | ** |
| 3wkCKR-3wkCKL | novel_mir_75 | 0.4293 | 16.6429 | 5.27677686 | 4.88315466140043e-68 | ** |
| 3wkCKR-3wkCKL | novel_mir_8 | 41.3944 | 70.2543 | 0.76315092 | 8.32975792063425e-27 |  |
| 3wkCKR-3wkCKL | novel_mir_81 | 2.3917 | 2.1246 | -0.17084519 | 0.636196361614087 |  |
| 3wkCKR-3wkCKL | novel_mir_86 | 0.01 | 1.2040 | 6.91169158 | 1.99313843195994e-06 | ** |
| 3wkCKR-3wkCKL | novel_mir_88 | 109.0971 | 53.7531 | -1.02119289 | 1.47542520583815e-64 | ** |
| 3wkCKR-3wkCKL | novel_mir_89 | 9.0148 | 7.8611 | -0.19756428 | 0.278548783536122 |  |
| 3wkCKR-3wkCKL | novel_mir_96 | 0.9199 | 18.6259 | 4.33968929 | 4.35225985305475e-68 | ** |
| 3wkCKR-3wkCKL | novel_mir_97 | 2.0850 | 0.01 | -7.70390356 | 6.6039639180129e-10 | ** |
| 3wkCKR-3wkCKL | novel_mir_99 | 0.01 | 349.0054 | 15.09096174 | 0 | ** |
