# Supplementary material for: Genome-wide analysis of salt-responsive and novel microRNAs in Populus euphratica by deep sequencing
Source: BMC Genet. 2014 Jun 20;15(Suppl 1):S6. doi: 10.1186/1471-2156-15-S1-S6 (PMC4118626; doi:10.1186/1471-2156-15-S1-S6)
Supplement: Additional file 12 — Significant expression changes in novel miRNAs in the leaves of salt-treated Populus euphratica (3dSL) and untreated root (3dSR) libraries. [file 1471-2156-15-S1-S6-S12.doc]

Additional file 12 - Significantly expression changed of novel miRNAs identified in *P. euphratica* between treated leaf (3dSL) and treated root (3dSR) libraries.

| pairwise | miR-name | 3dSR-std | 3dSL-std | fold-change(log2 3dSL/3dSR) | p-value | sig-lable |
| --- | --- | --- | --- | --- | --- | --- |
| 3dSR-3dSL | novel_mir_100 | 3.9987 | 0.01 | -8.64338722 | 2.75024683062099e-20 | ** |
| 3dSR-3dSL | novel_mir_106 | 1.7053 | 8.8187 | 2.37054043 | 1.21486251043654e-20 | ** |
| 3dSR-3dSL | novel_mir_109 | 1.0585 | 0.01 | -6.72587746 | 6.79012397689438e-06 | ** |
| 3dSR-3dSL | novel_mir_11 | 7.6446 | 4.1904 | -0.86735305 | 4.41950033802658e-05 |  |
| 3dSR-3dSL | novel_mir_111 | 4.2927 | 10.7575 | 1.32538558 | 6.07278916752386e-12 | ** |
| 3dSR-3dSL | novel_mir_114 | 3.6459 | 0.01 | -8.51013118 | 1.467142833531e-18 | ** |
| 3dSR-3dSL | novel_mir_119 | 4.0575 | 8.5059 | 1.06787280 | 2.62977327591587e-07 | ** |
| 3dSR-3dSL | novel_mir_122 | 7.7622 | 5.7540 | -0.43190038 | 0.0270089042030234 |  |
| 3dSR-3dSL | novel_mir_124 | 1.5289 | 0.01 | -7.25635025 | 3.38124576828239e-08 | ** |
| 3dSR-3dSL | novel_mir_127 | 2.3522 | 0.01 | -7.87786691 | 3.15627656131294e-12 | ** |
| 3dSR-3dSL | novel_mir_128 | 38.6346 | 0.01 | -11.91567799 | 7.86412897675826e-190 | ** |
| 3dSR-3dSL | novel_mir_13 | 0.4116 | 4.5031 | 3.45160363 | 7.25200460405221e-16 | ** |
| 3dSR-3dSL | novel_mir_131 | 0.01 | 9.8194 | 9.93949106 | 3.89664063024554e-50 | ** |
| 3dSR-3dSL | novel_mir_134 | 23.5806 | 0.01 | -11.20338478 | 3.84841152302008e-116 | ** |
| 3dSR-3dSL | novel_mir_135 | 0.01 | 5.2537 | 9.03719001 | 3.61514531022336e-27 | ** |
| 3dSR-3dSL | novel_mir_136 | 3.7047 | 0.01 | -8.53321291 | 7.5617493205287e-19 | ** |
| 3dSR-3dSL | novel_mir_143 | 0.4704 | 1.0632 | 1.17645304 | 0.0530499374866792 |  |
| 3dSR-3dSL | novel_mir_152 | 0.01 | 20.1391 | 10.97578350 | 4.76424288424988e-102 | ** |
| 3dSR-3dSL | novel_mir_158 | 0.3528 | 1.3760 | 1.96355800 | 0.00132950245600373 | ** |
| 3dSR-3dSL | novel_mir_17 | 0.01 | 4.0028 | 8.64486572 | 7.0888178024749e-21 | ** |
| 3dSR-3dSL | novel_mir_178 | 0.01 | 1.3134 | 7.03716255 | 2.39517436158455e-07 | ** |
| 3dSR-3dSL | novel_mir_182 | 20.8168 | 77.5542 | 1.89745660 | 1.43585254106572e-127 | ** |
| 3dSR-3dSL | novel_mir_187 | 0.01 | 2.3767 | 7.89281600 | 1.07338689035392e-12 | ** |
| 3dSR-3dSL | novel_mir_188 | 0.01 | 1.2509 | 6.96682265 | 4.94264634500382e-07 | ** |
| 3dSR-3dSL | novel_mir_19 | 0.4116 | 3.3774 | 3.03659817 | 5.33057155017548e-11 | ** |
| 3dSR-3dSL | novel_mir_192 | 1.1173 | 0.01 | -6.80387279 | 3.49967393733702e-06 | ** |
| 3dSR-3dSL | novel_mir_204 | 2.5874 | 0.01 | -8.01535928 | 2.22727872017469e-13 | ** |
| 3dSR-3dSL | novel_mir_23 | 14.8188 | 6.6296 | -1.16043489 | 4.43735303462911e-13 | ** |
| 3dSR-3dSL | novel_mir_233 | 0.01 | 1.0632 | 6.73226920 | 4.34336918092615e-06 | ** |
| 3dSR-3dSL | novel_mir_237 | 0.01 | 8.9438 | 9.80474412 | 9.89473104076765e-46 | ** |
| 3dSR-3dSL | novel_mir_238 | 0.01 | 119.7712 | 13.54799342 | 0 | ** |
| 3dSR-3dSL | novel_mir_239 | 0.01 | 20.3267 | 10.98916030 | 5.42159017519215e-103 | ** |
| 3dSR-3dSL | novel_mir_244 | 1.5877 | 3.3148 | 1.06198349 | 0.00143466284614986 | ** |
| 3dSR-3dSL | novel_mir_255 | 1.2937 | 0.01 | -7.01535930 | 4.79156320661528e-07 | ** |
| 3dSR-3dSL | novel_mir_295 | 0.01 | 4.0653 | 8.66721801 | 3.43519513015402e-21 | ** |
| 3dSR-3dSL | novel_mir_30 | 1.7641 | 0.01 | -7.46278853 | 2.38603195920321e-09 | ** |
| 3dSR-3dSL | novel_mir_316 | 0.01 | 2.4392 | 7.93026425 | 5.20156325251811e-13 | ** |
| 3dSR-3dSL | novel_mir_32 | 266.3261 | 64.1073 | -2.05463327 | 0 | ** |
| 3dSR-3dSL | novel_mir_389 | 2.1170 | 0.01 | -7.72587745 | 4.47275935483805e-11 | ** |
| 3dSR-3dSL | novel_mir_412 | 0.01 | 1.0632 | 6.73226920 | 4.34336918092615e-06 | ** |
| 3dSR-3dSL | novel_mir_42 | 6.8213 | 0.01 | -9.41390292 | 4.19339606287269e-34 | ** |
| 3dSR-3dSL | novel_mir_44 | 0.6469 | 61.3554 | 6.56750380 | 3.32058309771086e-287 | ** |
| 3dSR-3dSL | novel_mir_467 | 0.01 | 1.2509 | 6.96682265 | 4.94264634500382e-07 | ** |
| 3dSR-3dSL | novel_mir_468 | 0.01 | 1.0632 | 6.73226920 | 4.34336918092615e-06 | ** |
| 3dSR-3dSL | novel_mir_47 | 1.7641 | 1.4385 | -0.29436712 | 0.468289581858487 |  |
| 3dSR-3dSL | novel_mir_48 | 0.01 | 1.0007 | 6.64486572 | 8.96291232547411e-06 | ** |
| 3dSR-3dSL | novel_mir_483 | 1.1173 | 0.01 | -6.80387279 | 3.49967393733702e-06 | ** |
| 3dSR-3dSL | novel_mir_484 | 1.2937 | 0.01 | -7.01535930 | 4.79156320661528e-07 | ** |
| 3dSR-3dSL | novel_mir_487 | 1.1761 | 0.01 | -6.87786693 | 1.80375464562243e-06 | ** |
| 3dSR-3dSL | novel_mir_49 | 53.5710 | 580.7809 | 3.43846988 | 0 | ** |
| 3dSR-3dSL | novel_mir_51 | 379.7014 | 2761.0548 | 2.86228230 | 0 | ** |
| 3dSR-3dSL | novel_mir_52 | 0.5292 | 8.3809 | 3.98522021 | 1.0441359730671e-31 | ** |
| 3dSR-3dSL | novel_mir_53 | 28.0498 | 58.9162 | 1.07067390 | 3.70263113130766e-42 | ** |
| 3dSR-3dSL | novel_mir_55 | 54.1590 | 49.2219 | -0.13790075 | 0.0488927050269254 |  |
| 3dSR-3dSL | novel_mir_59 | 602.5709 | 132.5927 | -2.18412965 | 0 | ** |
| 3dSR-3dSL | novel_mir_6 | 2.7050 | 3.8152 | 0.49613009 | 0.077527998849881 |  |
| 3dSR-3dSL | novel_mir_62 | 3.9987 | 15.6359 | 1.96725931 | 2.51098689384195e-28 | ** |
| 3dSR-3dSL | novel_mir_63 | 3.5871 | 0.01 | -8.48667414 | 2.84657425516302e-18 | ** |
| 3dSR-3dSL | novel_mir_65 | 1.1173 | 1.3134 | 0.23328975 | 0.609847410751987 |  |
| 3dSR-3dSL | novel_mir_7 | 0.01 | 2.0639 | 7.68922926 | 4.01668986770336e-11 | ** |
| 3dSR-3dSL | novel_mir_70 | 0.01 | 2.3767 | 7.89281600 | 1.07338689035392e-12 | ** |
| 3dSR-3dSL | novel_mir_75 | 0.01 | 31.8973 | 11.63921859 | 3.38050956572825e-161 | ** |
| 3dSR-3dSL | novel_mir_8 | 20.4052 | 278.7573 | 3.77200083 | 0 | ** |
| 3dSR-3dSL | novel_mir_81 | 0.7057 | 6.5045 | 3.20431125 | 5.54960843389593e-21 | ** |
| 3dSR-3dSL | novel_mir_84 | 2.8814 | 0.01 | -8.17062612 | 8.10072640789687e-15 | ** |
| 3dSR-3dSL | novel_mir_85 | 0.01 | 3.1272 | 8.28872768 | 1.80006195613932e-16 | ** |
| 3dSR-3dSL | novel_mir_88 | 22.6986 | 29.6457 | 0.38521954 | 9.40596367325346e-05 |  |
| 3dSR-3dSL | novel_mir_89 | 4.5280 | 7.1925 | 0.66761936 | 0.00152121103852017 |  |
| 3dSR-3dSL | novel_mir_90 | 0.01 | 2.4392 | 7.93026425 | 5.20156325251811e-13 | ** |
| 3dSR-3dSL | novel_mir_95 | 1.8229 | 0.01 | -7.51009162 | 1.22977634719053e-09 | ** |
| 3dSR-3dSL | novel_mir_96 | 1.9406 | 16.5115 | 3.08889651 | 2.46607162706003e-49 | ** |
| 3dSR-3dSL | novel_mir_99 | 29.5199 | 306.0264 | 3.37389638 | 0 | ** |
